# A Meta-Assembly of Selection Signatures in Cattle

**DOI:** 10.1371/journal.pone.0153013

**Published:** 2016-04-05

**Authors:** Imtiaz A. S. Randhawa, Mehar S. Khatkar, Peter C. Thomson, Herman W. Raadsma

**Affiliations:** Reprogen - Animal Bioscience Group, Faculty of Veterinary Science, The University of Sydney, 425 Werombi Road, Camden, 2570, NSW, Australia; CSIRO, AUSTRALIA

## Abstract

Since domestication, significant genetic improvement has been achieved for many traits of commercial importance in cattle, including adaptation, appearance and production. In response to such intense selection pressures, the bovine genome has undergone changes at the underlying regions of functional genetic variants, which are termed “selection signatures”. This article reviews 64 recent (2009–2015) investigations testing genomic diversity for departure from neutrality in worldwide cattle populations. In particular, we constructed a meta-assembly of 16,158 selection signatures for individual breeds and their archetype groups (European, African, Zebu and composite) from 56 genome-wide scans representing 70,743 animals of 90 pure and crossbred cattle breeds. Meta-selection-scores (MSS) were computed by combining published results at every given locus, within a sliding window span. MSS were adjusted for common samples across studies and were weighted for significance thresholds across and within studies. Published selection signatures show extensive coverage across the bovine genome, however, the meta-assembly provides a consensus profile of 263 genomic regions of which 141 were unique (113 were breed-specific) and 122 were shared across cattle archetypes. The most prominent peaks of MSS represent regions under selection across multiple populations and harboured genes of known major effects (coat color, polledness and muscle hypertrophy) and genes known to influence polygenic traits (stature, adaptation, feed efficiency, immunity, behaviour, reproduction, beef and dairy production). As the first meta-assembly of selection signatures, it offers novel insights about the hotspots of selective sweeps in the bovine genome, and this method could equally be applied to other species.

## Introduction

Modern domestic species are a result of selective breeding for many traits of economic and adaptive importance since domestication [1–4]. The footprints of selective breeding on genomic architecture can now be characterized with the development of molecular genomic and advanced computational biology tools [5–7]. Although the contribution of natural (adaptation) and artificial (domestication and subsequent directional selection) remains challenging to differentiate, the rapid expansion of genomic data generated from global sequencing and genotyping projects are providing greater insights of selection on genomes of domestic species [8].

History of genetic changes in the bovine genome to adapt to new circumstances dates to about 10,000 years ago. Domestication followed by spatial dispersion due to human migration, has resulted in the population of many breeds of cattle across the world, exposing animals to new environments and husbandry practices [2, 4]. Therefore, long-term selection pressures have operated on the genomic regions that control traits for adaptive fitness. More recently, selection for various unique morphological traits during the development of specialized breeds (for example; coat colors, presence of horns, etc.) have left their selective signatures in the genome.

Modern cattle breeds are the most dominant and well-developed domesticated ruminant. They are raised for production of dairy, meat, leather and as draft animals [9, 10]. Dairying has underpinned the human cultural revolution as an important food source [11] along with influencing the human genome for selective adaptation to tolerate lactose in response to domestication of ruminants [12–15]. During the past 50 years the worldwide cattle population has increased approximately 50%, whereas, the production (milk, meat and hides) of this population has increased up to 100% (S1 Fig). This represents a remarkable improvement in both genetic value and husbandry practices. As such, the bovine genome has been exposed to intense selective forces for commercially important traits in many breeds.

Through different civilizations, humans have controlled breeding in cattle around the world that resulted in emergence of many contemporary breeds [1, 2, 4, 16–19]. It is thought that the progenitors (aurochs: *Bos primigenius*) of modern cattle were initially domesticated in the Euphrates Valley during the Neolithic era and then, within the next 2,000 years, they spread in the Fertile Crescent and further dispersed in the Mediterranean Basin [18]. It is presumed that subspecies of aurochs developed into various cattle types, and that the spatial distributions of multiple independent domestication events included different regions of the Fertile Crescent and Indus Valley and, possibly in Africa [2, 20–22]. Consequently, there are three distinct domestic cattle lineages, i.e., European *Bos taurus*, African *Bos taurus* and *Bos indicus* (Zebu), which represent all contemporary cattle breeds.

Recent investigations concluded that the taurine and indicine cattle diverged long before domestication, and an early separation in the ancestry of European, East Asian and African cattle breeds has resulted in widespread geographical distributions of taurine breeds [23–26]. Moreover, ancient and recent admixture between African taurine and indicine cattle has also been recorded [24, 25, 27, 28]. Present cattle breeds are believed to be remnants of a much larger cohort of ancient populations that once existed in isolation across various parts of the world. Bottlenecks due to domestication—breed formation and recent selection—have resulted in reduced effective population size of various geographically isolated populations [23].

Access to bovine genome sequence assemblies (S2 Fig) and high-density genotyping panels (S3 Fig) have provided researchers with remarkable resources to study the effects of domestication and selection on the architecture of the bovine genome. Subsequent investigations have revolutionized our understanding of mammalian evolution, domestication and devised strategies for enhancing genetic improvement for dairy and beef production [23, 29]. Organized breeding systems that have large well characterized cattle populations also provide pivotal resources for the discovery of genes contributing to complex traits such as milk production, fertility, muscle formation, energy partitioning and disease resistance [10, 12, 30, 31]. Approaches that integrate quantitative trait loci (QTLs) [32] mapping studies, genome wide association studies (GWAS) and selection signatures have led the way to fine-map and localize functional mutations of many genes contributing to phenotypic diversity in cattle [3].

Accurate identification of chromosomal regions that contain selection signatures is important in our understanding of the underlying genetic variation contributing to phenotypic diversity [3, 33]. Cattle have been a model animal for such studies with almost 1,000 breeds [34] reflecting genetic diversity underlying domestication, adaptation, appearance and production. This study reviews a set of 64 studies that have investigated selection signatures in worldwide populations of cattle breeds. We constructed a meta-assembly of the 56 genome-wide scans to highlight the hotspots of positive selection within cattle, and discuss the historical selection events and the role of underlying genes controlling the economically important traits. We also highlight the limitations of the available bovine genomic resources and implications of using different methodologies.

## Methods

### Meta-assembly of selection signatures in cattle

Many genome-wide scans have investigated unique patterns of genetic polymorphisms in the cattle genome to identify selection signatures (Table 1, S1 Table). These published studies used different SNP genotype datasets and various tests to detect such selection signatures (Table 2) across demographically diverse populations (S2 Table). These investigations provide insights about the historical selection in cattle but provide little information on consensus of selection signatures across the cattle genome. Recently, Gutiérrez-Gil et al. [35] reviewed and compared the genomic regions under selection across European cattle breeds, published in 21 studies. However, this study is the first attempt to comprehend the published results of 56 studies on selection signatures within each cattle type (African, European, Zebu and Composite) by developing a novel meta-assembly approach extending on the method used in Khatkar et al. [30], Khatkar et al. [31]. Construction of the meta-assembly was challenging given the heterogeneity of published investigations on selection signatures, hence several critical measures were adopted to minimize sources of potential bias in using available information.

**Table 1:** Summary of genome-wide scans on Bovine selection signatures.

No.	Study	SNPs (assay^1^)	Genome assembly^2^	Breeds (samples)^3^	Selection Tests^4^	Traits reported^5^
1	MacEachern et al. [48]	9,323 (10K)	Btau3.1	2 (713)	Fay & Wu's H, *F*_ST_, ΔDAF	Body composition, Carcass yield
2	Hayes et al. [49]	9,323 (10K)	Btau3.1	4 (774)	AFD, iHS	Milk and Meat production
3	Gibbs et al. [23]	37,470 (BHMC)	Btau3.1	19 (497)	*F*_ST_, iHS, CLR	Domestication traits: Behaviour, Immunity (MHC), Feed efficiency, Double Muscling, Milk yield and composition, Intramuscular fat content
4	Barendse et al. [121]	8,859 (10K) and 32,470 (BHMC)	Btau4.0	21 (385) and 19 (497)	*F*_ST_	Residual feed intake, Beef yield (Intramuscular fatness)
5	Gautier et al. [61]	36,320 (50K)	Btau4.0	11 (437)	*F*_ST_ (BF)	Adaptation (pathogens and climate), Trypanosomiasis tolerance, Immune response, Nervous system, Skin and hair properties
6	Flori et al. [106]	41,777 (50K)	Btau4.0	3 (2803)	*F*_ST_	Milk production, Reproduction, Coat color (Body coloration)
7	Chan et al. [60]	7,956 (10K)	Btau4.0	13 (317)	*F*_ST_, EHH	Tropical adaptation: Tick resistance, Heat resistance, Immune system
8	Stella et al. [85]	32,689 (BHMC)	Btau4.0	19 (497)	CLL	Polledness, Coat color (Black, Piebald), Dairy production, Reproduction
9	Qanbari et al. [122]	40,854 (50K)	Btau4.0	1 (810)	EHH, REHH	Milk yield and composition, Reproduction, Behaviour, Dairy quality
10	Gautier and Naves [46]	44,057 (50K)	Btau4.0	22 (725)	iHS, Rsb	Reproduction, Metabolism, Immunity
11	Noyes et al. [125]*	21,034 (BHMC)	Btau4.0	17 (86)	XP-EHH, Fay & Wu's H	Trypanosomiasis tolerance
12	Qanbari et al. [70]	~42,600 (50K)	Btau4.0	12 (3,876)	*F*_ST_, iHS	Reproduction (fertility), Muscle formation, Feed efficiency, Productive life
13	Glick et al. [156]	41,812 (50K)	Btau4.0	1 (912)	REHH	Milk (fat and protein) production and composition, Female fertility, Somatic cell score, Herd life
14	Hosokawa et al. [142]	40,635 (50K)	Btau4.0	2 (100)	SWAD	Carcass quality and yield, Milk (fat and protein) quality and yield
15	Larkin et al. [143]	52,150 (50K)	Btau4.0	1 (94)	AFD	Milk production, Fertility, Disease resistance
16	Schwarzenbacher et al. [157]*	34,851 (50K)	Btau4.0	1 (287)	iHS	Milk protein yield
17	Flori et al. [88]	47,365 (50K)	UMD3.1	19 (623)	*F*_ST_, iHS, Rsb	Polledness, Thermotolerance
18	Pan et al. [158]	40,130 (50K)	Btau4.0	1 (2106)	EHH, REHH	Milk production, Reproduction, Immune system, Growth
19	Pintus et al. [95]	42,514 (50K)	Btau4.0	2 (1,113)	*F*_ST_	Immune response, Production, Reproduction, Metabolism, Double muscles, Coat color
20	Boitard and Rocha [94]	35,554 (50K)	UMD3.1	1 (30)	HMM-SFS	Double Muscles, Body weight
21	Lee et al. [149]	15,125,420 (Sequencing)	UMD3.1	1 (12)	SFS (CLR)	Adaptive immunity, Domestication (BPV virus resistance)
22	Liao et al. [126]	9,990,733 (Sequencing)	UMD3.1	1 (14)	ZHp	Tropical adaptation: Resistance to hot temperature and tropical diseases (pathogens and parasites)
23	Lim et al. [159]	4522 (10K)	Btau3.1	1 (266)	iES	Beef production (intramuscular fat or marbling)
24	Utsunomiya et al. [141]	281,994 (800K)	UMD3.1	4 (136)	Meta-SS	Reproduction, Dairy and Meat production
25	Ramey et al. [62]	52,942 (50K) and 2,575,339 (AFFXB1P)	UMD3.1	15 (6,431)	Contiguously Low MAF	Behaviour, Polledness, Reproduction, Immune response, Ear floppiness, Beef production, Stature, Coat color
26	Druet et al. [86]	725,293 (800K)	UMD3.1	12 (674)	HMM-SFS	Polledness, Double muscle, Stature, Coat color
27	Porto-Neto et al. [103]	768,506 (800K)	UMD3.1	13 (505)	*F*_ST_	Divergent selection between Zebu and Taurine cattle (eg., Stature, Fertility, Production, Immunity)
28	Rothammer et al. [80]	47,651 (50K)	UMD3.1	10 (430)	XP-EHH	Polledness, Double muscle, Coat color, Dairy and beef production traits
29	Kim et al. [139]	41,951 (50K)	UMD3.1	1 (2,087)	iHS	Dairy production (milk, fat, protein yields)
30	Mancini et al. [104]	29,848 (50K)	Btau4.0	5 (2,797)	*F*_ST_	Reproduction, Lipid metabolism, Calving ease, Coat color, Stature, Dairy production
31	Edea et al. [138]	29,736 (50K)	UMD3.1	5 (47)	*F*_ST_	Metabolism, Hypoxia response, Heat stress
32	Fan et al. [69]	39,094 (50K)	UMD3.1	1 (942)	EHH, REHH	Beef production and meat quality (carcass traits)
33	Randhawa et al. [50]	38,610 (50K)	UMD3.1	21 (375)	CSS	Polledness, Double muscle, Stature, Coat colors, Reproduction, Immunity
34	Porto-Neto et al. [28]	680,000 (800K)	UMD3.1	9 (1,842)	*F*_ST_	Immunity, Reproduction, Metabolism
35	Kemper et al. [123]	610,123 (7K, 50K, 800K)	UMD3.1	8 (23,641)	*F*_ST_, iHS, HAPH	Polledness, Coat colors, Stature, Milk production, Double muscle
36	Perez OBrien et al. [140]	~ 510,320 (800K)	UMD3.1	4 (235)	VarLD	Adaptation, Dairy and Beef production, Reproduction (Male fertility), Ear morphology
37	Qanbari et al. [105]	15,182,131 (Sequencing)	UMD3.1	1 (43)	iHS, CLR	Domestication traits: Coat colors, Neuro-behaviour, Immune system, Sensory perception
38	Lee et al. [128]	17,666,906 (Sequencing)	UMD3.1	2 (21)	XP-EHH, XP-CLR	Milk proteins and production, Coat colors
39	Kim and Rothschild [160]	Not given (50K)	UMD3.1	1 (162)	iHS	Adaptation
40	Ryu and Lee [161]	11,799 (50K)	Btau4.2	1 (71)	CLL	Meat quality, Marbling score
41	Somavilla et al. [162]	560,565 (800K)	UMD3.1	1 (789)	REHH	Growth, Feed efficiency, Meat quality, Fatty acid profiles, Reproductive traits, Immunity
42	Xu et al. [163]	710,681 (800K)	UMD3.1	5 (169)	*di* (*F*_ST_)	Milk yield and composition, Body size (Stature), Coat colors, Development, Disease resistance, Skeletal muscle formation, Mammary gland, Immune response, Nervous system
43	Bomba et al. [164]	44,271 (50K)	Btau4.0	5 (2813)	REHH	Milk and meat production
44	Gurgul et al. [165]	43,315(50K)	UMD3.1	2 (708)	SWAD	Milk production, Developmental process, Immune system, Fertility, Growth, Coat colors
45	Kim et al. [166]	37,154 (50K)	UMD3.1	1 (1602)	iHS, Rsb	Dairy production, Disease resistance, Immune response, Metabolism
46	Randhawa et al. [51]	38,033 (50K)	UMD3.1	28 (494)	CSS	Stature, Growth rate
47	Flori et al. [167]	38,100 (50K)	UMD3.1	1 (203)	iHS, Rsb	Adaptive admixture, Coat colors,
48	Bahbahani et al. [168]	46,171 (50K)	UMD3.1	1 (425)	*F*_ST_, iHS, Rsb	Immunity, Reproduction, Development, Heat tolerance
49	Choi et al. [169]	17,936,399 (Sequencing)	UMD3.1	2 (20)	ZHp	Meat production (Intra-muscular fat)
50	Gurgul et al. [170]	40,378 (50K)	UMD3.1	1 (201)	REHH	Skeletal muscle growth, Meat quality
51	Kasarda et al. [171]	30,538 (50K)	UMD3.1	1 (40)	*F*_ST_, iHS	Feed efficiency, Coat colors, Growth
52	Kim et al. [172]	45,632 (50K)	UMD3.1	1 (586)	iHS	MHC, Gastrointestinal nematode resistance
53	Li and Kim [173]	35,968 (50K)	Btau4.0	1 (547)	REHH	Meat and carcass production
54	Makina et al. [174]	21,290–45,657 (50K)	UMD3.1	6 (247)	*F*_ST_, Contiguously Low MAF	Tropical adaptation, Nervous system, Immune response, Production, Reproductive performance
55	Sorbolini et al. [175]	43,009 (50K)	UMD3.1	2 (774)	*F*_ST_, VarLD	Double muscling, Immunity, Behaviour, Reproduction, Metabolism, Bone morphogenesis
56	Zhao et al. [176]	705,234 (800K)	UMD3.1	7 (3122)	*F*_ST_, iHS	Milk production, Reproduction, Body size, Muscle formation, Coat colors

^**1**^ Genotyping SNPChip assays; **10K**: MegAllele^™^ Genotyping Bovine 10K SNP Panel, features almost 10,000 SNPs randomly (not very evenly spaced) spanning the bovine genome. **BHMC**: The International Bovine HapMap Consortium (genome-wide panel of 37,470 SNPs. **50K**: Illumina BovineSNP50 Genotyping BeadChip, features more than 54,000 (version 1: 54,001 and version 2: 54,609) evenly spaced SNP probes (~51.5kb) spanning the bovine genome. **800K**: Illumina BovineHD BeadChip, features 786,799 SNPs that uniformly span the entire bovine genome. **AFFXB1P**: Affymetrix Axiom^(TM)^ Genome-Wide BOS 1 Array Plate, comprise 648,855 SNPs representing genetic diversity of ~3 (out of 46) million SNPs across breeds that uniformly locate after each 1 kb along the bovine genome and 2.5 million (2,575,339) SNPs from the Affymetrix SNP validation data were used in the reported study. Chromosome-wise SNP density of each genotyping assay is shown in S2 Fig.

^**2**^ Bovine Genome assemblies that were according released as; **Btau3.1** in August 2006, **Btau4.0** in October 2007, **UMD3.1** in December 2009.

^**3**^ Breed names and breed-wise sampling and genotyping information is provided in S2 Table.

^**4**^ Complete names and description of selection tests are provided in Table 2.

^**5**^ Summary of major findings related to various traits of economic importance from each study.

* Two studies have not provided complete (extended) list of significant regions, however, they performed genome-wide scans, therefore, the significant regions from these studies have been included in the computation of meta-scores. Additional studies that used targeted sequencing, microsatellites and parse genotypes on few chromosomes are provided in S1 Table.

**Table 2:** A list of selection tests used in published studies on Bovine selection signatures.

Test	Description	Bovine references
Fay & Wu's H	**Fay and Wu's H test**: Detects an excess of high (compared to intermediate) frequency variants that are likely to have been influenced by positive selection [177].	[48, 125, 178]
AFD	**Allele Frequency Difference**: Detects positive selection as the difference between allele frequencies of two populations, specifically used as Sliding Window Average Difference (**SWAD**) for a set of adjacent SNPs by averaging the absolute values of AFDs calculated along the genome.	[49, 142]
*F*_ST_	**Fixation Index** (Population differentiations): Detects both newly arising and pre-existing variation under selection by measuring the allelic diversity between populations versus within population [179–182].	[23, 28, 48, 50, 60, 70, 87, 95, 103, 104, 106, 121, 123, 138, 168, 171, 174–176]
*di*	**Divergence** (locus specific divergence in breed *i*): Detects high levels of population structure in loci of breed *i* by standardizing ***F***_**ST**_ between breed *i* and other *j* breeds, by using all pairwise combinations of genome-wide ***F***_**ST**_ [183].	[163]
BF	**Bays Factor**: Detects divergence selection from Bayesian binomial frameworks for loci that show concordant differences in allele frequencies across populations (such as *F*_ST_) with respect to specific aspects of the selective pressures [184, 185].	[61]
CLR	**Composite Likelihood Ratios**: Detects selective sweeps by modelling the spatial (chromosome-wise) distributions of allele frequency under the selection versus neutrality within a population, in addition, taking care for ascertainment bias, recombination rate and demography [186]. Recently, XP-CLR is used for across population analyses.	[23, 105, 128]
CLL	**Composite Log Likelihood**: Detects positively selected regions of the genome by comparing the frequencies of major (common) alleles for a set of contiguous loci between the samples of a unique sub-population and a larger panel of diverse populations.	[85]
HMM-SFS	**Hidden Morkov Model—Site Frequency Spectrum**: Detects positive selection within a population at regions of reduced heterozygosity by modeling the correlation structure between linked sites that uses site frequency spectrum and the spatial pattern of diversity among the sequence or polymorphism [56].	[86, 94]
Low MAF	**Low Minor Allele Frequency**: Detects complete selective sweeps within the population considering the cluster of adjoining loci carrying very low MAF (< 0.01).	[62, 174]
ΔDAF	**Change in Derived Allele Frequency**: Detects positively selected new causal mutations as the difference in the derived (non-ancestral) allele frequencies between populations [150].	[48, 50, 141]
ZHp	**Z-transformed Heterozygosity Value**: Detects selective sweeps by counting alleles in a sliding window centered on a candidate SNP, then calculates heterozygosity scores (Hp) from the pool of samples from within a population (breed) and extreme (negative) Z-transformed Hp values represent reduction in heterozygosity in the candidate regions [187].	[126, 141, 169]
VarLD	**Variation in Linkage Disequilibrium**: Detects candidate regions under positive selection by comparing genome-wide LD variation between populations [188] and it is implemented in the varLD program [189].	[140, 175]
HAPH	**Haplotype Homozygosity**: Detects strong positive selection (hard sweeps) by comparing the frequency of the core haplotype against the total number of haplotypes observed within the breed (population) that implements the neutrality tests based on the distribution of haplotypes under an infinite-site model [64, 65, 103, 140, 155, 190].	[123]
EHH	**Extended Haplotype Homozygosity**: Detects positively selected regions carrying frequent haplotypes with unusually high long-range LD patterns within a population [191, 192].	[60, 69, 93, 122, 193]
REHH	**Relative EHH**: Detects evidence of recent selection on relatively high frequency haplotypes within a population by comparing the **EHH** of the tested core haplotype to that of other core haplotypes present at a locus to correct for local variation in recombination rates [191].	[69, 122, 156, 170, 173]
XP-EHH	**Across Population EHH**: Detects selective sweeps by comparing **EHH** across populations in which selected alleles (at core haplotypes) have risen to near fixation in one but not all populations [194].	[50, 80, 125, 128]
iHS	**Integrated Haplotype-homozygosity Score**: Detects evidence of recent positive selection at a locus based on the differential levels of LD surrounding a positively selected (derived) allele (at intermediate frequencies) compared to the background (ancestral) allele at the same position within a population [73].	[23, 46, 49, 70, 88, 105, 123, 139, 141, 157, 167, 168, 171, 172, 176, 195]
iES	**Integrated extended haplotype homozygosity at SNP site**: Detects recent positive selection by finding lower levels of EHH decay, by estimating locus-wise (overall rather than a particular allele) haplotypic homozygosity over a two-way distance each SNP site, using a counting algorithm implemented to genotypic (non-phased) data at within a population [196].	[159]
Rsb	**Across Population iES**: Detects recent selection on completely or nearly fixed selective sweeps by comparing the single locus **iES** associated with the same site and genomic region across populations [196]. Rsb and XP-EHH are based on similar assumptions to target haplotype decay, so they can be substituted.	[46, 88, 141, 167, 168]
Meta-SS	**Meta-analysis of Selection Signals**: Detects evidence of recent positive selection in common variations by combining P-values obtained from Gaussian cumulative distribution function of **ΔDAF**, **Rsb**, **iHS** and **ZHp** tests.	[141]
CSS	**Composite Selection Signal**: Detects positively selected genomics regions carrying highly differentiated loci and underlying variants hauling excess haplotype in the samples of a target population versus phenotypically contrasting populations using the rank distribution approach to unify the multiple pieces of selection evidence from ***F***_**ST**_, **ΔDAF** and **XP-EHH** tests.	[50, 51]

### Construction of meta-assembly

#### Data collection

A total of 64 publications were identified which reported selection signatures in cattle, out of these, 56 genome-wide scans of selection signatures in cattle (Table 1) were compiled for the computation of meta-scores. The remaining eight studies (S1 Table) investigated selective sweeps on targeted regions of a few chromosomes or used other genomic markers (e.g., microsatellites), and were not included in the analyses to avoid any bias from single locus results [36] and could have overestimated the strength of selection in some regions, therefore distorting the meta-scores.

Many different approaches were used in the genome-wide studies to test the departure from neutrality. Most were based on estimates of population allele differentiation and haplotype homozygosity (Table 2). For the purpose of this review, selection tests were considered independent of each other and no adjustments were made for the perceived relationships across various selection tests. This position was taken considering that all tests within a study were mostly independent as presumed by those authors. Additional parameters that may cause inaccuracies or bias in the meta-assembly, such as differing bovine genome-maps, shared relationships among sample population, and significance threshold of reported signatures were included in the adjustment of the meta-assembly scores.

#### Mapping results onto UMD3.1 bovine assembly

Published results have been presented on different bovine genome assemblies, including Btau 3.1, Btau 4.0 and UMD3.1 (Table 1), which have variable genome coverage (S2 Fig). In order to find alignments of detected selection signatures across studies, results from Btau 3.1 and Btau 4.0 were mapped to the latest UMD3.1 bovine genome assembly. Studies which used different SNP genotyping panels (Table 1, S3 Fig) were updated using the common or nearest marker position for the start, end and middle (or top score location, if given) of a selection signature using common reference markers across panels.

#### Breeds and groups of breed-types

All breeds and crossbred cattle from the study population can be divided into four breed-type groups, according to their geographical origins, namely; European (*Bos taurus*), African (*Bos taurus*), Zebu (*Bos indicus*) and composite (*Bos taurus* × *Bos indicus*) and are detailed in S2 Table. Since the historical events of selection in these groups have been unique, the data from these four groups were organized and analysed separately. Further information on the origin of breeds, sample size, SNP density, bovine genome assembly, selection tests are also shown in S2 Table.

The populations listed in S2 Table are considered input datasets and the output (results) have been presented according to populations detailed in S3 Table. Based on the input datasets, the populations of 86 breeds have been used either as breed-wise, group-wise (grouped with other breeds) or as a reference panel (S3 Table). For example, Holstein has been investigated in 33 studies, where 22 studies presented results for the breed, seven studies presented Holstein results grouped with other populations and four studies have used Holstein samples as a reference panel to investigate selection signatures in other breeds (S3 Table). Overall, breed-wise results were available from 53 cattle breeds representing 1 to 22 studies. Moreover, 72 breeds have been presented as group-wise results consisted of 1 to 7 studies, whereas 33 breeds were used as various reference panels consisted of 1 to 4 studies (S3 Table).

The published results complied as breed-wise and group-wise selection signatures were used to compute meta-scores. For each position of published selection signature, two adjustment factors were computed: 1) to adjust for the use of repeated DNA samples across studies and 2) to account for the significance thresholds used for the top SNPs within each study. These adjustment factors are termed as “DNA score” and “SNP score”, respectively, and are described in detail below.

#### Common usage of DNA samples across studies

It was noted that several breed-wise genotyping datasets were re-analyzed across different studies using different analytical tools. For example, DNA samples (breeds) from the Bovine Hap Map Consortium (BHMC) and subsequently genotyped with bovine Illumina BovineSNP50 (50K) and BovineHD (800K) BeadChip assays, have been used repeatedly in several studies (S2 Table). Furthermore, several studies have used new samples combined with samples used previously. Therefore, for every breed, which was investigated in multiple studies, the proportion of common individuals between each pair of *i* and *j* study was computed to account for common usage of DNA samples.

If *n*_*i*_, *n*_*j*_ are the total number of DNA samples and *n*_*ij*_ is the number of common DNA samples between each pair of studies *i* and *j*, and there are *k* studies on a particular group/breed *b*, then the DNA score *d*_*i*_ can be computed for breed *b* of study *i* as follows:

$$d_{i}=\;\frac{1}{\sum_{j=1}^{k}\left(\frac{n_{ij}}{\left(n_{i}+\;n_{j}\right)-\;n_{ij}}\right)}$$

**Fig 1:**
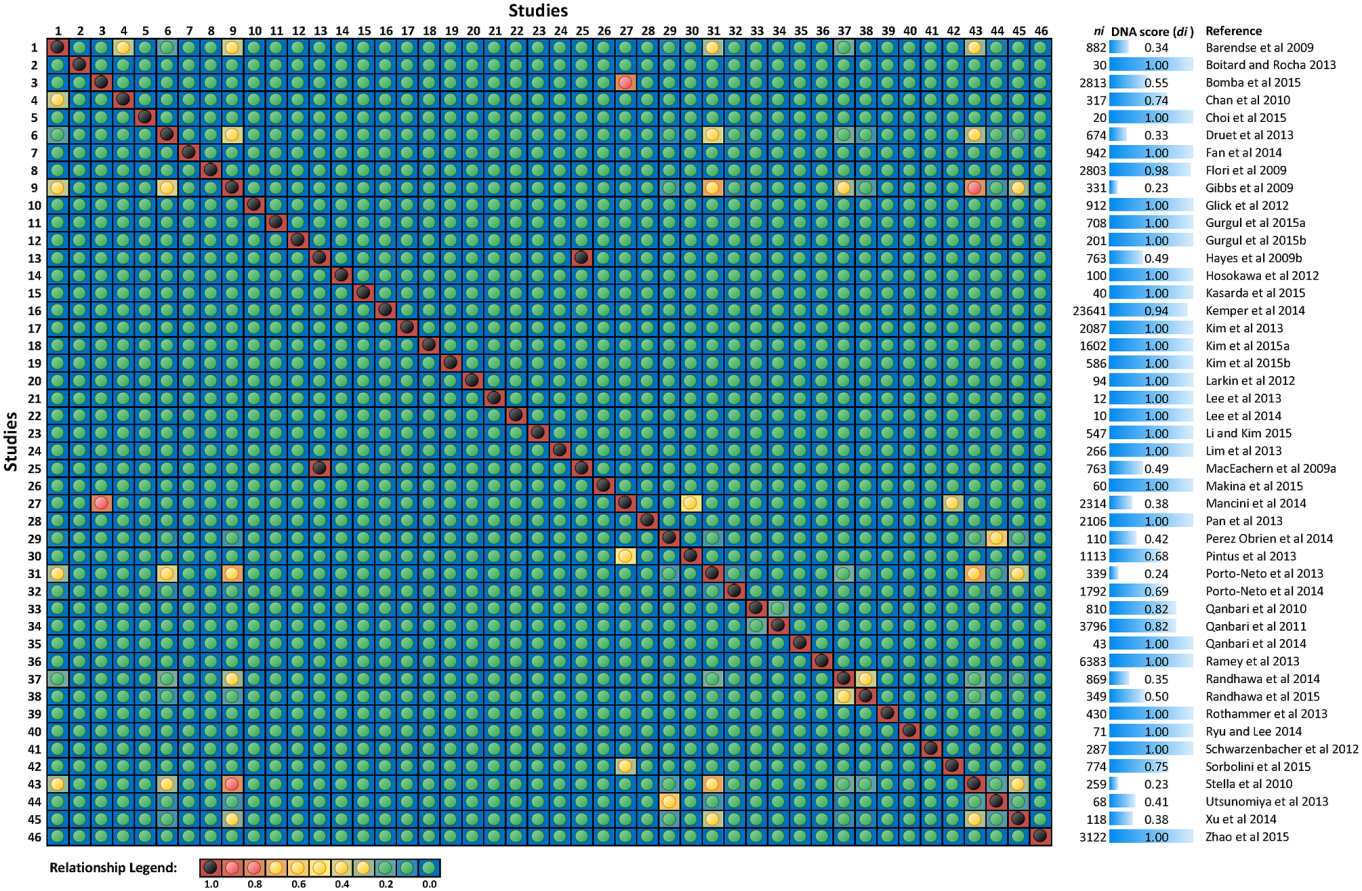
Plot of relationship matrix and DNA score (*d*_*i*_) weighting computed from 46 studies that published selection signature using European cattle. ***n***_***i***_ shows total number of DNA samples from European breed(s) from a particular study. The relationship score was computed as proportion of common samples between each pair of studies and it range between 0 and 1.

It is noteworthy that not all studies provided results for individual breeds; rather a selection signature was reported for a group of multiple breeds with a common defining attribute, e.g., polled, dairy, African or Zebu breeds. In those cases, common usage of the total number of DNA samples across multiple studies was computed as the DNA score using the above formula. Similarly, the DNA score for the subset of breeds represented in a particular group was computed e.g., a group represented by European breeds (Fig 1). Moreover, DNA scores *d*_*i*_ were re-computed for each analysis using the individual breed for which *k* > 1 published results of selection signatures were investigated.

#### SNP score (*s*)

The different significance thresholds used across studies to declare significant selection signatures (regions or SNPs under selection) could distort the signal under a meta-assembly. Hence, a SNP score *s*_*i*_, was assigned to provide an approximately equal contribution from each investigation (i.e., study *i*). Across all studies the range of significance thresholds vary from the top 5% to top the 0.1% using various distributions of results, such as, *p*-values, *q*-values, percentiles etc. A simplistic approach was implemented to define *s*-values, so that the set of published selection signatures within the top 5%, 1% and 0.1% thresholds were weighted as *s* = 1, *s* = 2 and *s* = 3, respectively.

#### Weighted selection signatures (*SS*)

The DNA score (*d*_*i*_) and SNP score (*s*_*i*_) values were used to assign a weighted selection signature (*SS*) score at each unique position. For instance, for each breed-wise or group-wise result from any study *i*, at a locus *l*, the $SS_{i_{l}}$ score was equal to *d*_*i*_ × *s*_*i*_.

#### Computation of meta-selection-score (MSS) across studies

Finally, the meta-score of selection signatures, termed as “meta-selection-score” (MSS) at each unique reported position, locus *l*, was computed as the sum of *SS* value within a sliding window (2 or 5 Mb) from multiple studies:

$$MSS_{l}=\sum_{i=1}^{k}SS_{i_{l}}$$

If a study has reported multiple positions within the window then only highest *SS* was included. It is noted that the span of the published selection signatures ranged from a single bp to 46.10 Mb (S4 Fig). For the selection signature reported as spans, the span overlapping with the sliding window were included in the computation of MSS.

A weighted *SS* from an individual study (for a breed or group) can contribute a maximum value of 3, when the DNA samples were completely independent (*d*_*i*_ = 1) and the significance threshold was in the top 0.1% (*s*_*i*_ = 3). Notably, multiple tests of selection within a study were standardized for repeat sampling relationship in *d*_*i*_ and for their individual thresholds of significance in *s*_*i*_. Hence, a MSS value above 3 represents a selection signature detected more than once (i.e., independently validated).

A higher magnitude of the MSS at a genomic position shows the consistency of a selection signature detected across studies within a breed or within a group. All peaks above the validation value of MSS > 3 were used to report the validated genomic regions under selection for each population, separately for the groups and breeds. The first and last positions of each peak were used to define the boundaries of putative regions. In general, all peaks were clearly separated from each other within the groups and breeds. However, because of the high density and large number of published signatures of selection for the European group and extensively studied breeds (Holstein and Simmental), several consecutive peaks were overlapping at MSS > 3. Therefore, the boundaries of genomic regions under selection in European, Holstein and Simmental meta-assemblies were defined where consecutive peaks intersect each other, i.e., the span of lowest MSS value(s) between two consecutive peaks.

#### Sliding-window spans for genome-wide MSS

S5 Fig compares a detailed analysis of the most important MSS peaks localizing classic selective sweeps in the European, Angus and Holstein meta-assembly by using 5 Mb and 2 Mb sliding window spans. The smaller (2 Mb) sliding window spans provides a narrow MSS peak around the candidate gene regions. However, the power of the meta-assembly analyses to fine map the hotspots of selection depends upon the number of available *k* studies. Hence, using a smaller window size can have profound effect at the genomic regions harbouring putative sweeps investigated in fewer studies. For example, out of the 25 cattle breeds analysed, the magnitude of MSS using 5 Mb spans in the genome-wide distribution of eight breeds have not been found above the minimum validation value (MSS = 3). Using the smaller window spans (1–2 Mb) can eliminate further breeds that have been investigated in fewer studies and show prominent (MSS > 3) peaks using larger (5 Mb) spans. Hence, the size of the sliding window for the computation of *MSS*_*l*_ was set to 5 Mb, i.e., ~ 2.5 Mb on each side of a locus *l*.

## Results

### MSS maps of cattle

MSS were computed for all 4 groups and 25 breeds where published results were available from multiple studies (*k* ≥ 2). Moreover, the selection signatures from an additional 28 breeds (S6 Fig), where only a single study (*k* = 1) was available (S3 Table), contributed to the group-wise MSS only. In total 16,158 (European = 13,640, Zebu = 1,246, African = 1,112 and Composite = 160) individual selection signature scores contributed to the meta-assembly.

#### MSS maps of cattle groups

**Fig 2:**
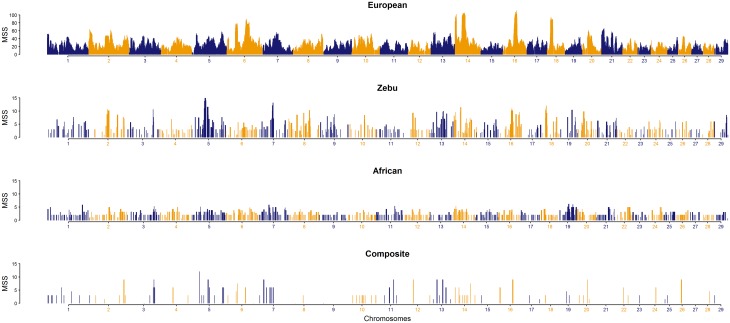
Meta-assembly of selection signatures in four groups within European, Zebu, African and Composite breeds of cattle.

Meta-assemblies of European, Zebu, African and Composite groups were constructed from 46, 12, 9 and 8 studies, respectively (Fig 2). A comparison of MSS, gene density and individual published signatures of selection (showing their origin from studies, breeds, selection tests, SNP panels and significance thresholds) is presented for each chromosome of European *Bos taurus* group (S7 Fig). Overall peaks with extreme MSS in a meta-assembly map identify the hotspots of positive selection within cattle groups (Fig 2).

In the European group, the meta-assembly shows that significant signatures of selection have been detected across the whole genome. To define genome-wide coverage of published signatures of selection, the bovine genome was divided into 1 Mb non-overlapping windows. More than 90% of the 1 Mb windows contained a published signature of selection, and a maximum gap of 4.47 Mb between the consecutive genomic regions under selection was observed on bovine autosome (BTA) 1. The top 5% of MSS (MSS ≥ 75) of the meta-assembly were located on BTA-6, BTA-14, BTA-16 and BTA-18. In addition, most of the chromosomes harbour prominent peaks within the top 25% (MSS ≥ 35), except BTA-15, BTA-23 and BTA-28, which have their top peaks above 50% (MSS ≥ 25).

On the other hand, because of limited number of investigations and breeds of Zebu, African and composite cattle, the genome coverage in these groups was limited relative to European *Bos taurus* investigations. In the Zebu group, major MSS peaks in the top 5% (MSS ≥ 11) were located on BTA-5, BTA-7, BTA-14, BTA-16 and BTA-18. In the African group, the MSS peaks in the top 5% (MSS ≥ 5.2) were located on BTA-1, BTA-3, BTA-7, BTA-11, BTA-14 and BTA-19. In the composite group, the MSS peaks in the top 5% (MSS ≥ 9.0) were located on BTA-2, BTA-3, BTA-5, BTA-7, BTA-11, BTA-12, BTA-13, BTA-16, BTA-20 and BTA-28.

In total, 439 prominent peaks of validated genomic regions (i.e., clusters of MSS > 3) were detected genome-wide for the four archetype groups (S4 Table), distributed across European = 173, African = 116, Zebu = 120 and Composite = 30 respectively (Table A in S1 File). Out of those, 141 validated genomic regions under selection were found putatively unique for each group viz. European = 64, African = 37, Zebu = 36 and Composite = 4. On the other hand, 298 peaks from the four archetype groups (European = 109, African = 79, Zebu = 84 and Composite = 26) shared at 122 genomic regions (Table A in S1 File). The 298 co-aligning peaks (122 shared regions) were common between at least two groups, such that; 36 peaks were shared at nine regions between all four groups, 108 peaks at 36 regions were shared between any three groups and 154 peaks at 77 regions were shared between any two groups (Table B in S1 File). Overall, there were 263 hotspots of positive selection at prominent peak scores found within (*n* = 141) or shared across (*n* = 122) the four cattle archetypes ranging in size from 0.1 to 12.34 Mb.

#### MSS maps of cattle breeds

**Fig 3:**
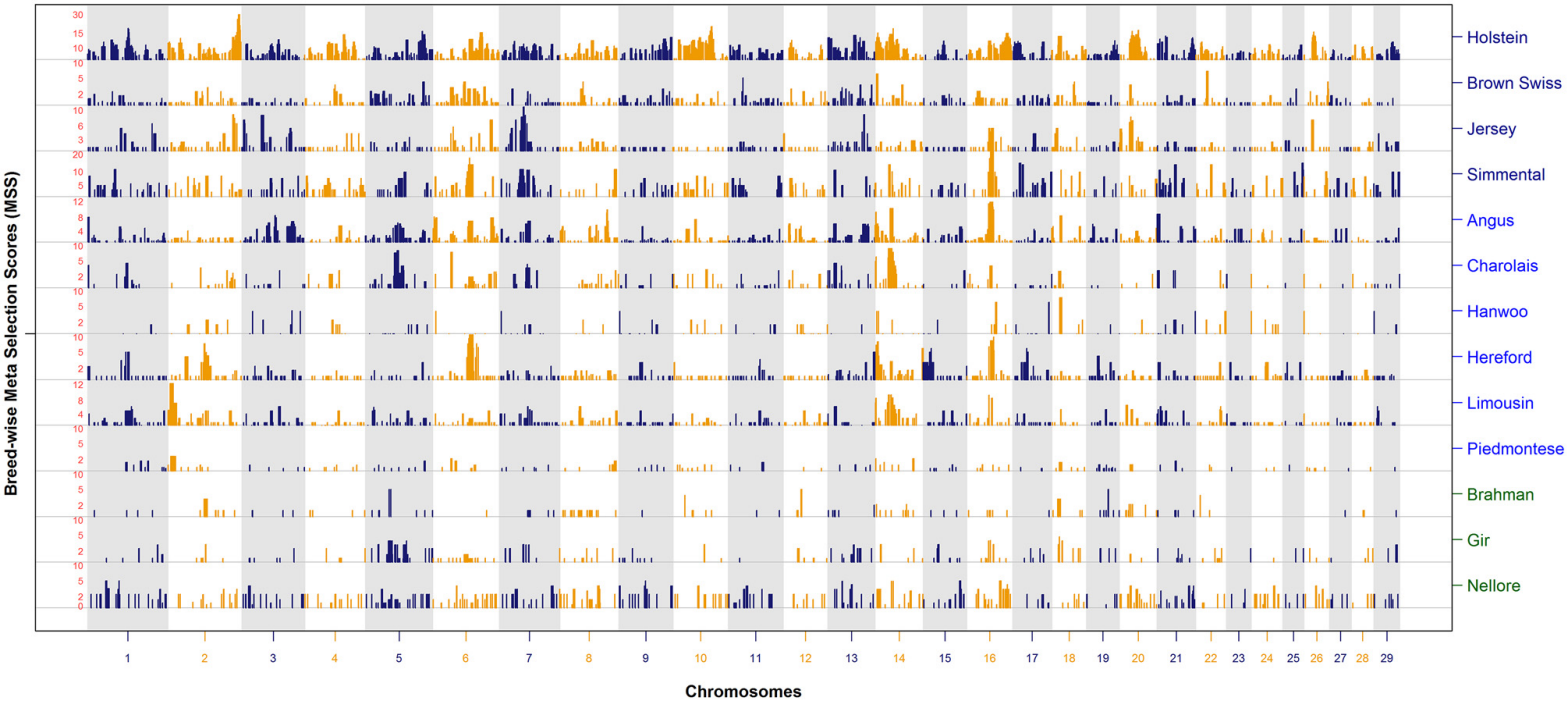
Meta-assembly of selection signatures of Holstein, Brown Swiss, Jersey, Simmental (Fleckvieh), Angus, Charolais, Hanwoo, Hereford, Limousin, Piedmontese, Brahman, Gir and Nellore cattle.

**Fig 4:**
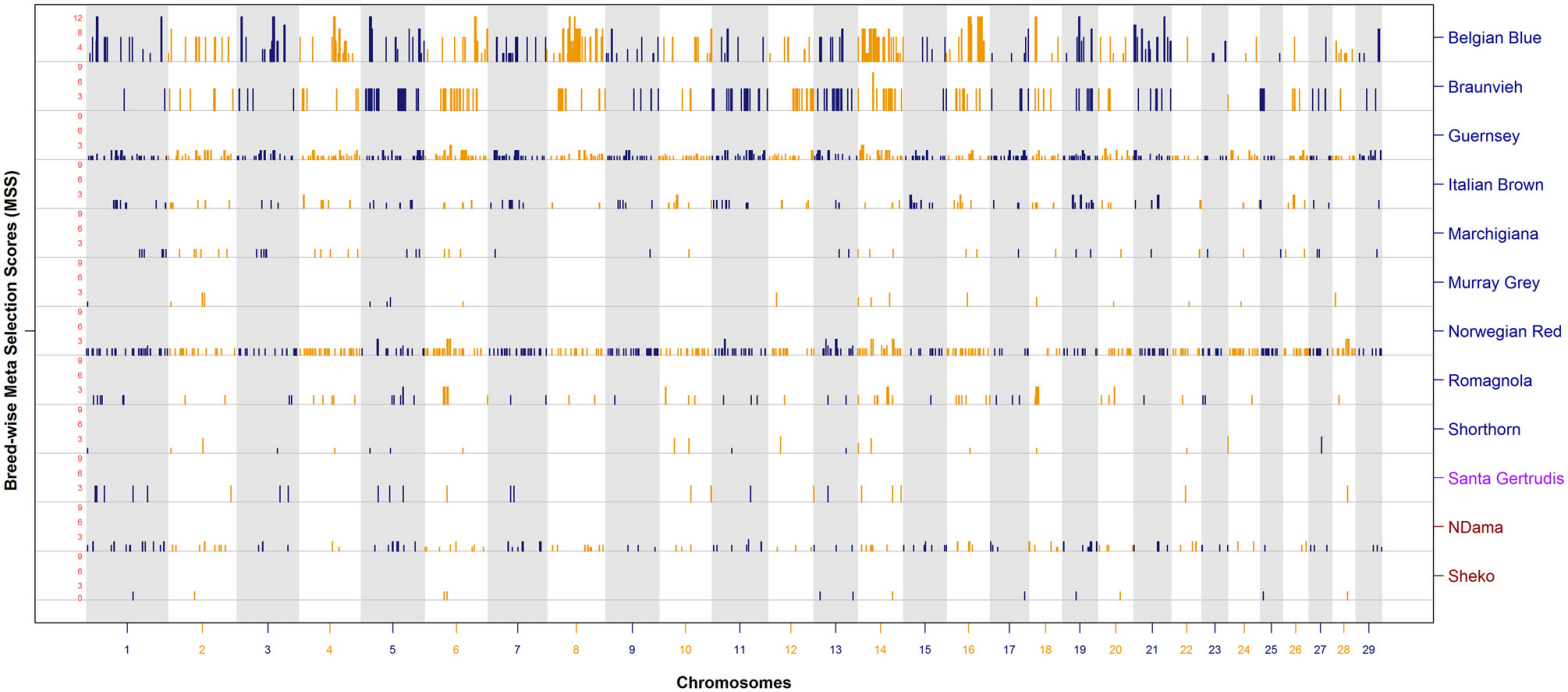
Meta-assembly of selection signatures of Belgian Blue, Braunvieh, Guernsey, Italian Brown, Marchigiana, Murray Grey, Norwegian Red, Romagnola, Shorthorn, Santa Gertrudis, NDama and Sheko.

Fig 3 shows the meta-assembly of selection signatures across 13 breeds, each constructed from at least *k* = 5 studies, where as MSS for a second cohort of 12 breeds which were represented in less than five (*k* = 2 to 4) are shown in Fig 4. The meta-assemblies of these 25 breeds have been constructed using signatures of selection from 2 to 22 studies (S3 Table).

The breed-specific meta-assemblies show that several genomic regions have been consistently detected within and across breeds. However, only 16 of the 25 breeds showed validation (MSS > 3) of selection signatures across independent studies. Lack of consistency was seen in the breeds with limited studies (*k ≤* 3); for example, Guernsey, Italian Brown, Murray Grey, NDama, Norwegian Red, Santa Gertrudis, Sheko and Shorthorn (S3 Table). There are some exceptions for Belgian Blue (*k* = 4), Braunvieh (*k* = 3) and Romagnola (*k* = 2). Several signatures of selection at the lower levels of the significance threshold did not achieve validation (MSS > 3) in some breeds (Fig 4).

Overall, 854 prominent peaks of validated MSS were detected for 16 breeds. The complete list of validated MSS peaks, their location and the genes underlying each region is provided in S5 Table, and their unique or shared localization is summarized in Tables A and B in S2 File. In summary, out of the 854 peaks located at 341 hotspots under selection, 113 peaks were found unique within 15 breeds and 741 MSS peaks were shared across 2 to 12 breeds at 228 hotspots (Table A in S2 File). Out of the 228 shared regions, 170 (494 peaks) were found across multiple breeds within European archetype, whereas, 58 (247 peaks) were found shared between breeds of the European and Zebu archetypes (Table B in S2 File). No unique or shared validated MSS peaks were found in African or composite breeds. A direct comparison with the subset of European breeds reviewed earlier [35] was not practical as we report validated MSS and earlier study presented unprocessed list of selection signatures as core selective sweep regions.

#### Genomic regions under selection across groups and breeds

**Fig 5:**
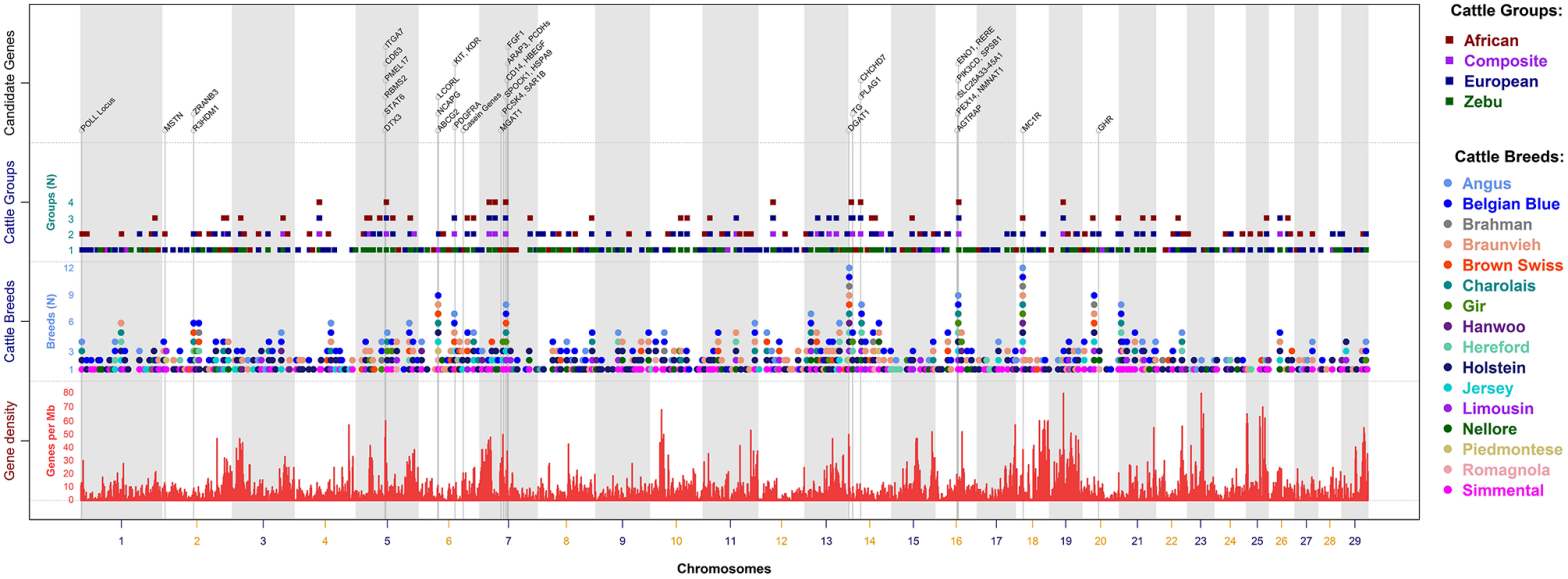
Map of selection signature hotspots captured in the meta-assembly of cattle breeds and groups. Middle Panels labelled as “Cattle Breeds” and “Cattle Groups”, show the location of prominent regions in the cattle breeds and groups, respectively represented with the unique colours as shown in the legends. The clustered dots, within a locus, located on top of each other represent shared selection signatures across the breeds and groups, each of which has been validated in multiple investigations. Lower Panel labelled as “Gene density” shows distribution of bovine genes on each chromosome that ranges 0–80 genes/Mb (S7 Fig shows high-resolution comparison of MSS and genes per Mb in European). Top Panel labelled as "Candidate Genes" shows genomic locations of a few major genes underlying the outstanding peaks representing classic selective sweep regions in the meta-assemblies. Complete list of prominent regions, localized top MSS and underlying genes within the groups and breeds of cattle are respectively shown in S4 and S5 Tables.

Fig 5 illustrates the comparison between the prominent regions across the 4 cattle archetypes and 16 cattle breeds. In cattle groups, the nine shared validated MSS peaks across all four archetypes were located on BTA-4, BTA-5, BTA-7, BTA-12, BTA-14, BTA-16 and BTA-19. In cattle breeds, most of the shared signatures of selection, in four or more breeds, were located on all but BTA-23, BTA-24, BTA-25 and BTA-28. Common genomic regions across various populations reveal historical selection shared between those cattle breeds, most likely due to ancestral (within archetypes), geographical and/or commercial similarities. Unique genomic regions under selection, which are private to a single breed, can be responsible in shaping particular characteristics of the population resulting in the origin and maintenance of that breed. Several of the important genes known for their role in some major traits have been labelled (Fig 5) and complete list of genes underlying each region within groups and breeds are provided in S4 and S5 Tables, respectively. Nevertheless, the number of genes within each hotspot of selection varied between 0 to 382 due to the variable span of MSS and gene density in the bovine genome (Fig 5).

#### Comparison of meta-assembly and gene density

To align the prominent regions with gene density, the number of genes within 1 Mb spans were counted along the genome. On average, bovine genome contains approximately 9 genes per Mb and 7.5% of all 1 Mb regions are without any genes. A chromosome-wise comparison of meta-assembly and gene density for the European (S7 Fig) shows that signatures of selection have been detected in gene-dense as well as gene-poor regions. Using the spans of prominent peaks, we also compared genome-wide average gene density against the number of genes in each prominent region (S4 and S5 Tables). Many hotspots of selection localized in genomic regions with medium-to-high gene density. However, some prominent peaks within low-to-medium density genic regions indicate that selection targeted a few or single genes of major effects, for example, *MSTN* (BTA-2), *ABCG2* (BTA-6), *NCAPG-LCORL* (BTA-6), *PLAG1-CHCHD7* (BTA-14), and *GHR* (BTA-20). Inferring the candidate genes underlying signatures of selection at high gene-density regions is challenging. Patterns of genetic diversity implicating strong selective sweeps may have been generated by a cumulative effect of selection acting on multiple genes, for example, the regions identified in multiple populations at chromosome 5, 7 and 16 (Fig 5).

## Discussion

### Insights from meta-assembly

This study implements a novel approach to infer hotspots of positive selection in the bovine genome by using diverse set of published selection signatures across worldwide cattle breeds. The approach of computing meta-scores and construction of a meta-assembly map of selection signatures can be widely used where conventional meta-analysis methods cannot be applied in scenarios similar to this study. The meta-assembly highlights several genomic regions of variable gene density where positive selection has been replicated within and across breed-populations. In general, MSS across multiple breeds show that selection has acted on candidate genes; however, the span of selection is generally wide because of LD and hitchhiking in the target regions. The hotspots of selection could be linked to various biological functions enriched across those breeds, especially related to adaptation (disease, climate, feed resources), appearance (polledness, coat colors) and production (milk, meat, fertility) traits, each of which have economic importance in various environments and production systems. Several regions harbouring candidate genes controlling major traits, e.g., coat color (*MC1R*, *KIT*), stature (*NCAPG*, *LCORL*, *PLAG1*) and milk production (*ABCG2*, *DGAT1*, *GHR*) are consistently identified under strong selection in multiple breeds of cattle (Fig 5). There are also clearly strong selection signatures at some genomic regions, e.g., chromosome 7 and 16, where the associations for QTLs and functional variants of multiple traits have been localized. Overall, the genomic regions underlying prominent peaks of meta-scores provide the evidence of QTLs for the traits being constrained in cattle populations. Limited efforts for gene-networks and functional analyses for the selectively differentiated regions across cattle breeds warrant extensive resources in future.

Several pitfalls in construction of the meta-assembly originate from the limitation of individual studies and tests of selection. Ascertainment bias and introgression in the Zebu and African breeds resulted in lack of Zebu and African specific selection signatures. Using *F*_ST_ or similar tests of selection, which lack directionality to point the population under selection, can lead to misleading interpretations about selection signatures. Another common reason of finding coinciding genomic regions under selection relates to the use of reference populations across the European, African and Zebu breeds. Moreover, most of the breeds tested for selection in non-European cattle, for example Zebu, are not representatives of overall breed-type. The investigated breeds (Brahman, Nellore, Gir etc.) might have experienced bottlenecks because the samples used in selection signature scans are from imported herds into non-native countries that started from a few hundred founder animals, rather than representatives of the natural populations. To date, limited investigations on African and Zebu breeds are available; therefore, conclusions are limited since these studies are mostly underpowered for these two archetypes as compared to the European breeds.

The tests of selection, which were implemented in the cattle studies, have been established and validated with human genomic data. Therefore, some of the intrinsic problems can relate to the differences in genetic architecture and demographic history of the two species. Establishing (fine-tuning) these tests considering the demographic history of cattle, can fine scale the regions under selection [37, 38]. Nonetheless, there are several common phenomena of detecting selection signature between cattle and human. The span of the regions under selection is generally wide; however, most of the tests of selection, based on the extensively used outlier approaches, detect only a section of the candidate region. As there can be many proxy SNPs around the causal variants, the overlap between various genome-wide selection scans that are in common is rare in both species. Low replication rate may point to high incidence of false positives, hence, validation data sets are required to exclude the rate of false positives, along with accounting for all the challenges causing confounding effects that generate false positive results. Notably, the bovine genome appears to be completely covered with selection signatures especially in the group of European breeds.

Implementation of new approaches, such as a meta-assembly, can provide the insights to resolve the false and true positive regions under selection. The magnitude of the meta-score depends upon the span of sliding windows and the number of studies reporting the co-located signals. Hence, given sufficiently available studies, the larger and smaller window spans, respectively, can elucidate the overall broad and fine-mapped functional regions for each selection signature. The meta-assembly approach can potentially be improved by incorporating additional measures, such the effects of sample size, as they become available from future research.

### Challenges in identification of selection signatures

Critical factors that may influence the accuracy of individual studies and consequently the meta-assembly, are confounding effects of genomic structure within each breed (genetic drift, demographic effects, recombination rate, and age and type of selection) and methodological biases (sample size, ascertainment schemes, density and distribution of SNPs, power of the tests of selection, null distribution for significance threshold and selection models). Besides selection pressure, different demographic (non-selective) forces such as population size and gene flow can generate the patterns of population’s genetic diversity [16]. Moreover, distinguishing the effect for selection and demography of a population is challenging [39]. Hence, the population history of current breeds may incur convolution to the investigation of genomic history, i.e., patterns of genetic diversity [40–42]. In human, different demographic scenarios mimicked identity of hitchhiking patterns, which shows the importance of estimating underlying demographic model to accurately implicate the genomic regions for a selection event [43]. In cattle, geographically isolated evolution and domestication, introgression, co-ancestry, admixture, migration, bottlenecks, inbreeding, and variable population sizes intricate the insights of cattle history [18, 23–25, 27, 44–49]. Incorporating this knowledge into selection models could help differentiate the true positive selection from that of confounding patterns of genetic drift arising from non-selective forces of primeval demography. For example, combining different populations/breeds based on similar phenotype can help to detect trait specific signatures of selection [50, 51].

The quality of genetic polymorphism data also affects the population genetic inferences [52–57]. In cattle, ascertainment bias has occurred for the African and Zebu cattle because the development of various current genotyping assays (e.g., Illumina SNP50 BeadChip) primarily based on European breeds [58]. In addition to under-representation of rare variants in genotyped samples of the non-European cattle breeds [59], the consequences of ascertainment bias include limited SNP density. Multiple studies have reported limited set of polymorphic SNPs in the African and Zebu breeds as compared to the European data [24, 25, 46, 48, 50, 60–62]. Genome-wide low density and inconsistent distribution of genotypic panels may produce false results and can have a strong influence on the population genetic procedures being used to estimate selection signatures. Recently, Heslot et al. [63] suggests that increasing the marker density may reduce the effects of ascertainment bias and increase the accuracy of genetic diversity estimates. Hence, appropriate approaches should be implemented for the individual study data to correct the ascertainment bias.

In general, parameters of selection are inferred using estimates based on site frequency spectrum, population differentiation and haplotype length (extend of LD) [43, 64, 65]. Several approaches implement these estimators by comparing genetic diversity within or across population and across species to detect various types (modes) and age (time-frame) of selection [7]. Two major modes of selection (positive and balancing) can be easily distinguished by most of the basic estimates of selection [66, 67], which have been extensively implemented in cattle (Table 2). Nevertheless, there are several challenges related to various tests [36, 68], and use of inappropriate approaches provide misleading conclusions [67]. Notably, because of the varying levels of statistical power, consistency of results across various tests of selection using common datasets is limited [43, 69]. For example, the genotype (*F*_ST_) and haplotypes (iHS) based estimates detected selection signatures at different genomic regions in cattle [70]. The single marker (genotype) based estimates, in general, are constrained being sensitive to ascertainment bias and have several limitations [71]. On the other hand, haplotypes are based on patterns at multiple SNPs and, therefore, haplotype based estimates of selection are less sensitive to ascertainment schemes of SNP discovery for the genome-wide panels.

Tests of selection are generally criticized for their limitation of sample size and approaches to declare significant (outlier) loci. Adequacy of the sample size can be established by the effective population size and SNP density that can capture maximum genetic diversity for the population under investigation. Various investigations have provided the range of sample size to be sufficient to detect regions under positive selection for beneficial traits being carried out by applying various measures including allele and haplotype frequencies and extent of LD [72, 73]. These studies suggest that some of the commonly used selection tests (*F*_ST_, XP-EHH) have reasonable power for the available genetic resources of cattle. However, the breeds genotyped by low-density SNP panels are likely to provide less reliable results. Recently available high-density genotypic data, albeit on limited sample size, enable high power to the selection tests, such as *F*_ST_ [74], however, substantial decline in power still occurs for rare genetic variants [75, 76]. Approaches of combining different tests into a single index, such as CSS [50], can overcome some of the limitations of individual tests.

Failure to detect truly positive signatures of selection often relates to the approaches used to declare the significance level, especially in the absence of null distributions for the selection tests. Generally, estimates of across-population differences in allele and haplotype frequency imply selection using the so-called outlier loci using an arbitrary cut-off [42, 77], however, the lack of empirical evidence supporting this assumption spawn criticism [36, 78]. The genomic regions experiencing mild selective pressure are not detectable by the outlier approaches [68]. On the other hand, demographic effects and the frequent occurrence of cold spots of recombination in the genome can dominate the outlier distribution and confound selection [65, 79]. The complex demography and evolutionary diversity of cattle still pose a challenge to be dealt to control false positive selection signatures [80].

### Challenges related to meta-assembly

The biases associated with the challenges of individual selection signature investigation have direct impact upon the computation of meta-scores. For example, several common regions identified between the European, African and Zebu breeds, shown at prominent peaks in the breed-type groups (Fig 2), indicate the impact of reference populations being used. It appears that most of these common regions have originated from the European breeds, however, because of non-directional presentation of the results from selection tests, such as *F*_ST_, have declared those regions significant in non-European breeds as well. The results from such tests of selection should be interpreted with care and within the context by requiring the evidence of direction of selection. Moreover, the selection tests (*F*_ST_, VarLD etc.) that lack the direction of selection should be implemented within the breed-types and analyses performed using breeds across the breed-types should be interpreted as demographic rather than selection signatures. The potential sources of additional bias may be due to factors including; across study variation in the sample sizes, SNP chip assay, data quality control filters, SNP density, selection tests, post-analyses transformation of result (smoothing, clustering etc.), lack of the magnitude of individual selection scores, and provision of full or partial lists of significant regions. With the available information about published selection signatures, it is not possible to account for these additional factors. Nevertheless, this meta-assembly provides a framework for alleviating some of the limitations of individual study and provides consensus regions under selection pressure. Future investigations would account for the pitfalls outlined above and provided the required information, subsequent methodological improvements to the computation of meta-scores are possible.

### Signatures of historical selection in cattle

This article, to our knowledge, is the first meta-assembly detailing a consensus profile of genomic regions influenced by the historical selection events. The meta-assembly shows some very strong and congruent signatures of selection in multiple breeds for historically selected traits, such as polledness, double muscling, coat colors and bovine stature. Several additional genomic regions harbour genes for traits with complex selection history, such as adaptation, reproduction, growth, and high performance of dairy and beef production. Interestingly, some genomic regions have been implicated for selective constraints on multiple genes of different traits. The candidate genes underlying strong selective sweeps, which are depicted by prominent peaks in the meta-assembly maps, are annotated in Fig 5. This section presents an overview of the traits and genes that have experienced long-term selective breeding.

#### Genes and traits linked to strong selective sweeps in cattle

The *POLL* locus at the high gene density proximal end of BTA-1 is associated with horn development [81–84]. The strong positive selection in multiple breeds for naturally polled animals have swept the functional mutation in the region to fixation in several European breeds [50, 58, 62, 80, 83, 85–88]. In Zebu, the complex nature of inheritance suggests additional loci may be involved in horn development, however, a diagnostic marker at the *POLL* locus shows its dominance effect for polledness [84, 89].

The bovine myostatin (*MSTN*, AKA *GDF8* i.e., growth differentiation factor 8) gene on BTA-2 controls the muscular hypertrophy (double muscle) phenotype and strong selective pressure has escalated the allele frequencies of functional mutations because of its economical imporatnce in beef breeds [16, 90–93]. Hence, classic selective sweeps are extensviely found harbouring the *MSTN* gene in several genome-wide scans in beef catlle [23, 50, 62, 86, 93–95].

The *KIT* (on BTA-6) and *MC1R* (on BTA-18) genes stipulate breed-specific colors in a polygenic inheritance pattern in cattle [96–98] and other species [3, 99–102]. The *KIT* locus has been under strong selection to control white-spotting in multiple cattle breeds [62, 80, 85, 86, 103–105]. Whereas, the *MC1R* locus strongly selected for coat color patterns that mediates the dark (black or brown) and light (yellow or red) shades via melanogenesis in cattle [62, 80, 85, 86, 95, 103, 106, 107]. The *KIT* neighbouring (*PDGFRA* and *KDR*) genes have also been considered under selection for their role in reproduction [46, 95, 106].

The *NCAPG-LCORL* (BTA-6) and *PLAG1-CHCHD7* (BTA-14) gene regions harbour functional variant for polygenic trait of stature, which have very high heritability [51, 108, 109]. Historical evidence shows that bovine ancesters (aurochs) had very large stature, and that initial selection decreased the height during the Middle Ages [18], and then reverse selection occured after the 17th century to increase bovine stature [2]. Several variants at *NCAPG*-*LCORL* and *PLAG1*-*CHCHD7* loci have been inferred as candidates for skeletal, carcase, growth and height related traits in cattle [110–116] and other mammal species [112, 117–120]. Selection signatures have been consistently localized at the *NCAPG-LCORL* region [23, 49, 50, 60, 80, 85, 86, 95, 103, 104, 106, 121–123].

#### Genes underlying traits with complex selection histories

Signatures of selection for adaptation are mainly attributed to tolerance in new climates, feed resources and resistance to different disease agents in various cattle breeds. The changes in genetic aspects of behavioural control for new adaptations, from survive to thrive, and using available resources have also been detected under positive selection in several populations of African and European taurines [23, 61, 62, 85, 122]. Hence, the low gene density genomic regions harbouring genes (e.g., *R3HDMI*, *ZRANB3*; BTA-2) for feed efficiency have shown the impact of selective forces [23, 46, 70, 95, 121]. Additional unique regions were also found under selection for controlling several physiological functions related to tropical climatic and pathogenic adaption in African and Zebu breeds. For example, the *slick* hair coat locus on BTA-20 [124] for thermotolerance in Senepol [88], Trypanotolerance related trait in African [47, 61, 125] and, tick and heat resistance genes in Zebu [60, 126].

A strong selective sweep at a region on BTA-14 harbouring *PLAG1* gene, associated with stature, is also associated with fertility traits [31, 103]. Similarly, breeds selected for high beef production also highlight the underlying selection for fertility traits, such as gamete generation, embryo development, and spermatogenesis [70]. Such selective sweeps are results of commercialization of farm animals in the recent past by implementation of genetic improvement programs for the core production traits to achieve maximum performance and to minimize the non-productive life spans that generally relates to reproductive inefficiencies.

Candidate genes on BTA-6 (*ABCG2*, Casein cluster), BTA-7 (*SAR1B*, *HBEGF*), BTA-14 (*DGAT1*), BTA-16 (*AGTRAP*, *KIF1B*) and BTA-20 (*GHR*) have been consistently localized under strong selective sweeps in multiple dairy breeds (Fig 5). Identified selection signatures in cattle show that selective forces operated on genetic architecture controlling the physiological and anatomical structure of mammary glands and quantity and quality of various milk components. Dairy production has been attributed to the cultural revolution providing an important source of human food [11] along with influencing the human genome for selective adaptation for tolerance to lactose in response to domestication of cattle [12–14, 127]. The selective pressures on these genes vary depending upon their functional importance in cattle. For instance, the casein genes (*CSN1S1*, *CSN2*, *CSN1S2* and *CSN3*) perform major role in milk protein quality, however, only limited genetic evidence for positive selection has been presented [122, 128]. On the other hand, *ABCG2*, *DGAT1* and *GHR* consistently found under selection signatures and investigated in further details. The *ABCG2* has been found involved in milk yield and composition [129–132] and a differential expression found it as lactation regulator [133]. Similarly, for dairy production, the *DGAT1* [134, 135] and *GHR* [80, 106, 136] are also strong candidate genes with major effect on milk yield and composition and the regional QTLs and strong selection signatures have been found coincided in multiple cattle breeds [30, 31].

*TG* (BTA-14) gene (Fig 5)–responsible for meat tenderness or intra-muscular fat distribution [137]–has been captured underlying the signatures of selection in multiple breeds and groups of cattle [49, 61, 69, 80, 86, 103, 126, 138–140]. Signatures of selection of some beef-specific traits—vital in shaping the beef breeds—include intra-muscular fat content [23, 80, 94, 121], muscle formation [62, 70, 86, 93–95], body composition and carcass yield [48, 80, 141, 142]. Feed efficiency (as discussed above) has been associated with intensive selective breeding for beef production and signatures of population differentiation have been detected in several breeds [23, 46, 70, 95, 121].

#### Genomic regions selected for multiple traits

The color (*KIT*) and dairy (*ABCG2*) associated genes co-locate with other genes related to reproduction and bovine stature, respectively on BTA-6 [123]. In addition, there are gene-rich regions on BTA-7 (41–55 Mb) and BTA-16 (40–50 Mb) harbouring several tightly clustered MSS for multiple traits, thus provide important information about the bovine genome. In these regions, the significant signatures have been implicated for different traits and explained to arise from multiple events of selective events.

On BTA-7, the region comprise of several candidate genes between *MGAT1* and *FGF1* (Fig 5). Both of these genes implicated with reproduction traits, due to their role in fertilization and subsequent embryonic development and growth [70]. Additional genes, *PCSK4* [140] and *SPOCK1* [23, 103] perform key functions in fertility, also considered important candidates of reproduction traits. On the other hand, *SAR1B* [143] and *HBEGF* [85], underlying a selective sweep located in the centre of the region identified in high producing dairy breeds (Fig 5), and the gene functions include milk production and disease resistance. A set of additional genes, including *HSPA9*, *CD14*, *ARAP3* (*CENTD3*) and multiple members of *PCDH*, in this region are also implicated in multiple investigations for their range of functions related to immune response [46, 103, 140, 141].

On BTA-16, the *AGTRAP* gene is involved in the functioning of mammary glands and has been implicated for dairy production [85]. Similarly, at the nearby location, the *KIF1B* gene was identified under strong selection in dairy Holstein cattle [106]. At the closest flanking region, *NMNAT1* [70] and *RERE* [62] genes were localized as candidates of positive selection for embryonic growth and reproductive development. In addition, highly differentiated loci and extended haplotype homozygosity underlain *SLC25A33* and *SLC45A1* genes in the region were characterized for their important role in immunity related to tropical adaptation [60]. Similarly, selective sweeps localizing *PIK3CD* and *SPSB1* genes were also implicated for recent selection pressures that underlies immune response and immune regulation, respectively [62].

### Prospects for future studies

Building on genome-wide selection scans needs to address the confounding factors related to demographic and sampling effects. Controlling for potential confounding factors in the data acquisition techniques and statistical designs are necessary for making accurate interpretations and implications. The ascertainment bias can be overcome with dense and uniform distributed SNPs available in the recently developed high-density SNP-chip assays. Utility of the several current datasets can be further extended by combining them [144]. In addition, imputation from lower to higher density SNP panels can be performed by using several approaches [145–147], as implemented by Kemper et al. [123] to increase sample size and SNP density. More recently, the genome sequencing and ultra-high density SNP panels available at affordable cost provide opportunities to perform extensive scans for genome-wide selection signatures [105, 126, 148, 149], and collection of these data will be useful for across breed investigations. Genome-wide scans of selection signatures in additional breeds of African and Zebu archetypes are required to characterize the archetype specific genomic regions due to their unique selection history and diverse geographical background.

New selection tests and robust approaches are emerging to deal with potential challenges, hence, care should be invested while choosing applicable statistical tests with good power under the given selection models. A very recent attempt includes combing several (semi) independent estimates that increase power and resolution [50, 141, 150]. To that end, additional cattle genomic resources, e.g., availability of ancestral and derived allelic phasing from out-groups and available datasets [25, 58, 151], further enable the robust estimates. Moreover, some of the across-breed estimates of selection can also be estimated better by using a reference panel from a pool of multiple breeds to generate a set of neutral genomes. Establishment of approaches to define significance thresholds and null distribution of the statistical tests being used are required to minimize the false positives. In addition, development of selection tests to detect genomic regions underlying complex traits (such as Randhawa et al. [51]) is required for identification of classic selective sweeps in cattle.

Recent investigations of the structural variation in cattle genome suggest that selective forces, in addition to the genotypic and haplotypic patterns, operate on the copy number variation (CNV) in candidate genes and can be helpful to characterize the effects of domestication, breed formation and artificial selection [152–155]. Hence, exploration of additional genomic features, such as CNVs as alternative targets of selection, can further help elucidate the prevalence of selection in the cattle genome [64, 65, 103, 140, 155]. Nonetheless, the role of other types of selection (e.g., standing, balancing) is also warranted to quantify the role of evolutionary forces in the genetic and phenotypic diversity of cattle. In addition, comparative analyses of meta-assembly of selection signatures against QTL and GWAS database can further highlight the genomic regions under selection for traits of economic and biological importance in cattle.

The ultimate objectives of genomic scans for evolving patterns of genetic diversity are to detect causative variants and its functional relevance to particular traits. Use of the high-throughput assays for generating dense genotypes and genomic sequences will aid the fine mapping of candidate variants. Finally, functional analysis of the detected variants in the regions and genes under hotspots of positive selection will be an active area of future research to understand the biological significance of molecular variations in adaptation, appearance and production in cattle.

## Supporting Information

S1 Table A summary of published studies using different polymorphism panels and partial scans on Bovine selection signatures. (PDF)

S2 Table Breed-wise data information about breed type, land of breed origin, country of sampling, DNA samples, SNPchip, SNPs, bovine assembly and selection tests for each published study. Breeds shown in **bold** have been used across multiple studies.
(PDF)

S3 Table List of cattle breeds categorized for the number of available study-wise results from genome-wide scans of selection signatures. (PDF)

S4 Table List of regions under selection, validated across multiple studies, based on Meta-assembly of four groups of cattle. (XLSX)

S5 Table List of regions under selection, validated across multiple studies, based on Meta-assembly of 16 breeds of cattle. (XLSX)

S1 File **Summaries of** S4 Table, such that; Table A: Summary of 439 validated meta-selection-score (MSS) peaks found unique within and shared across the four groups of cattle. Table B: Summary of 298 validated regions shared across multiple archetypes at 122 hotspots of selection regions.
(XLSX)

S2 File **Summaries of** S5 Table, such that; Table A: Summary of 854 validated meta-selection-score (MSS) peaks found unique within and shared across the 16 breeds of cattle. Table B: Summary of 741 validated regions shared across multiple breeds (within and/or across archetypes) at 228 hotspots of selection regions.
(XLSX)

S1 Fig Bovine population (A), milk production (B), beef production (C) and hide production (D) in various top 20 countries of the World in 2010–11 (pie charts) and population trends in the past 50 years (trend lines).
(PDF)

S2 Fig Chromosome-wise length (million base-pairs or Mbp) of bovine genome assemblies (Btau3.1, Btau4.0, Btau4.6 and UMD3.1). (PDF)

S3 Fig Chromosome-wise (x-axis) SNP density (y-axis) of various genotyping Bovine SNPchip assays; **A**: 10K, **B**: BHMC (mapped on Btau 3.1 and Btau 4.0), **C**: 50K (from two version, v1 and v2) and **D**: Illumina’s 800K and Affymetrix AFFXB1P (~700K but features for ~2.5 million SNPs).
(PDF)

S4 Fig Distribution of the size (Mb) of selection signature regions of cattle published in 56 studies. (TIFF)

S5 Fig Meta-selection-scores (MSS) in European group, Holstein and Angus highlighting classic selective sweeps on various chromosomes. The left (A, C, E, G) and right (B, D, F, H) panels show chromosome-wise MSS using 5 Mb and 2 Mb sliding window spans, respectively. **A-B**: Chromosome 6 of European group highlighted at *ABCG2*, *LAP3*, *NCAPG* and *LCORL* gene located between 37.95–39.00 Mbp (blue bar) and at *PDGFRA* and *KIT* genes located between 71.37–71.42 Mbp (pink bar). **C-D**: Chromosome 14 of European group highlighted at *DGAT1* (1.69–1.96 Mbp; blue bar), *TG* (9.26–9.51 Mbp; green bar) and *PLAG1*-*CHCHD7* (25.00–25.06 Mbp; pink bar) region. **E-F**: Chromosome 16 of Angus highlighting span (44.45–45.88 Mbp) between *NMNAT1* to *RERE* genes (blue bar). **G-H**: Chromosome 20 of Holstein highlighting *GHR* region at 31.89–32.07 Mb (blue bar).
(PNG)

S6 Fig Genome-wide distribution of selection signatures of 28 breeds (Anatolian Black, Belmont Red, Blonde dAquitaine, Finnish Ayrshire, Franken Gelbvieh, Galloway, Illyrian Mountain Busa, Japanese Black, Korean, Murnau-Werdenfelser, Normande, Pinzgauer, Polish Red, Red Angus, Salers, Wagyu, Yanbian, Afrikaner, Bonsmara, Borgou, Drakensberger, Nguni, East African Shorthorn Zebu, Guzera, Beef Master, Creole, Kenyan crossbred, Senepol), each of which have results available from a single published study. Hence, Meta-score cannot be computed for these breeds, however, these results have been used accordingly to the particular breed-type in various group-wise Meta-assemblies of European, Zebu, African and Composite breeds.
(TIFF)

S7 Fig Comparison of Meta-assembly and signatures of selection regions on 29 bovine autosomes (BTA) identified across 46 studies in European breeds of cattle. Top panel shows Meta-selection-scores (MSS) computed for the group of all European breeds and provides a comparison of MSS and gene density (genes per Mb) distribution on each chromosome. Lower panel shows location of published selection signature regions. Symbol and colour of each dot point represent selection test (symbols in the bottom legend) and candidate breed (European breed coloured legend on the left), respectively. In lower panel, the central or top (if given) score region is shown with a dot point and spans of extended regions are shown with a horizontal solid-line. Labels on right-hand side of lower panel shows the SNP genotyping panel used in the particular study and the red, orange and pink colours of each SNP panel represent study-wise threshold of top 0.1%, 1% and 5%, respectively.
(PDF)
