# Supplementary material for: A Meta-Assembly of Selection Signatures in Cattle
Source: PLoS One. 2016 Apr 5;11(4):e0153013. doi: 10.1371/journal.pone.0153013 (PMC4821596; doi:10.1371/journal.pone.0153013)
Supplement: S1 Table — (PDF) [file pone.0153013.s001.pdf]

S1 Table. A summary of published studies using different polymorphism panels and partial scans on Bovine selection signatures.

Study	SNPs and markers (assay)	Genome assembly	Breeds (samples)	Selection Tests[1]	General findings
**(Freeman et al. 2008)**	Sequencing (6 genes)	Btau3.1	16 (39)	Tajima's D, Fu & Li's D, Fu & Li's F, Fu's FS, Fay & Wu's H	Immunity
**(Hayes et al. 2008)**	403 (BTA-6)	Btau3.1	1 (716)	iHS, dN/dS	Milk production
**(Prasad et al. 2008)**	BTA-19, BTA-29	Btau3.1	2	EHH, RAAF	Beef and dairy production
**(Dayo et al. 2009)**	92 Microsatellites	Btau3.1	14 (509)	lnRV, lnRH	Trypanotolerance resistance (adaptation)
**(Liu et al. 2009)**	6,769 SNPs (10K)<br>294 Microsatellites and<br>3 Haplotypes	Btau3.1	1 (800)	QTL mapping, association	Pigmentation
**(MacEachern et al. 2009b)**	9,323 (10K)	Btau3.1	11 (3)	dN/dS, MKT	Gene evolution
**(Wiener and Gutierrez-Gil 2009)**	16 SNPs,<br>16 Microsatellites (BTA-2)	Btau4.0	12 (571)	EHH, LD	Double Muscling
**(Li et al. 2010)**	51 Microsatellites	Btau4.0	10 (366)	FST, Ewens-Watterson	Polled and horned, Milk yield, Body composition

[1]The list of selection tests used in these studies is provided on next page. Other selection tests are described in Table 2.

Description of selection tests:

Test	Description	Reference
Tajima's D	**Tajima's D:** Distinguish between randomly and non-randomly evolving DNA sequences (Tajima 1989).	(Freeman et al. 2008)
Fu & Li's D; Fu & Li's F	**Fu & Li's D test and Fu & Li's F test:** These tests detect background selection based on coalescent by using genetic polymorphism from within and out-group species (Fu and Li 1993).	(Freeman et al. 2008)
Fu's FS	**Fu's FS test:** Detects population growth and genetic hitchhiking (Fu 1997).	(Freeman et al. 2008)
RAAF	**Rolling Average Allele Frequency: T**he frequency of the allele with the lowest frequency averaged over two participating breeds/populations, specifically using a (n=5) multi-loci sliding window for each chromosomes and for each pair of averages; the mean of first population allele frequency are subtracted from that for second population to get the mean allele frequency differences at the central locus.	Prasad et al. (2008)
dN/dS	**dN/dS Ratio:** Ratio of the rate of non-synonymous substitutions (dN) to the rate of synonymous substitutions (dS), which can be used as an indicator of selective pressure acting on a protein-coding gene (Nei and Gojobori 1986; Hughes and Nei 1988; Yang and Nielsen 2000).	(Hayes et al. 2008; MacEachern et al. 2009b)
MKT	**McDonald-Kreitman Test:** Estimate the proportion of substitutions that are due to adaptive evolution using the numbers of silent and non-silent polymorphisms and substitutions (McDonald and Kreitman 1991).	(MacEachern et al. 2009b)
Ewens-Watterson	**Ewens-Watterson test:** Detects positive selection from the estimates of population mutation rate.	(Li et al. 2010)
