# Supplementary material for: A Meta-Assembly of Selection Signatures in Cattle
Source: PLoS One. 2016 Apr 5;11(4):e0153013. doi: 10.1371/journal.pone.0153013 (PMC4821596; doi:10.1371/journal.pone.0153013)
Supplement: S2 Table — Breeds shown in bold have been used across multiple studies. (PDF) [file pone.0153013.s002.pdf]

**S2 Table.** Breed-wise data information about breed type, land of breed origin, country of sampling, DNA samples, SNPchip, SNPs, bovine assembly and selection tests for each published study. Breeds shown in **bold** have been used across multiple studies.

| Breed name | Code | Breed type | Land of Origin | Country of sampling | Samples | SNPchip | SNPs | Assembly | Selection tests | References |
|---|---|---|---|---|---|---|---|---|---|---|
| Abondance (Simmental) | ABD | European | France | France | 22 | 50K | 38,610 | UMD 3.1 | CSS | Randhawa et al 2014 |
| Afrikaner | AFN | African | South Africa | South Africa | 42 | 50K | 45,657 | UMD_3.1 | FST, Low MAF | Makina et al 2015 |
| Anatolian Black | ANB | European | Turkey | Germany | 43 | 50K | 47,651 | UMD 3.1 | XP-EHH | Rothammer et al 2013 |
| **Angus** | **ANG** | **European** | **Scotland** | Australia | 2510 | 800K | 692,527 | UMD 3.1 | $F_{ST}$, iHS, HAPS | Kemper et al 2014 |
| | | | | Australia | 379 | 10K | 9,323 | Btau 3.1 | iHS, AFD | Hayes et al 2009 |
| | | | | Australia | 379 | 10K | 9,323 | Btau 3.1 | H, DAFS | MacEachern et al 2009 |
| | | | | Australia | 232 | 50K | 44,938 | Btau 4.0 | $F_{ST}$, iHS | Qanbari et al 2011 |
| | | | | Australia | 42 | 10K | 7,956 | Btau 4.0 | $F_{ST}$, EHH | Chan et al 2010 |
| | | | | Australia | 41 | 10K | 8,859 | Btau 4.0 | $F_{ST}$ | Barendse et al 2009 |
| | | | | Austria | 31 | 800K | 575,082 | UMD 3.1 | VarLD | Perez Obrien et al 2014 |
| | | | | Canada | 103 | 50K | 40,595 | Btau 4.0 | $F_{ST}$, iHS | Qanbari et al 2011 |
| | | | | New Zealand and USA | 81 | 800K | 680,000 | UMD 3.1 | $F_{ST}$ | Porto-Neto et al 2014 |
| | | | | New Zealand and USA | 44 | 800K | 768,506 | UMD 3.1 | $F_{ST}$ | Porto-Neto et al 2013 |
| | | | | New Zealand and USA | 44 | 50K | 38,610 | UMD 3.1 | CSS | Randhawa et al 2014 |
| | | | | New Zealand and USA | 42 | 800K | 725,293 | UMD 3.1 | HMM-SFS | Druet et al 2013 |
| | | | | New Zealand and USA | 27 | BHMC | 7298 | Btau 4.0 | $F_{ST}$ | Barendse et al 2009 |
| | | | | New Zealand and USA | 27 | BHMC | 37,470 | Btau 3.1 | $F_{ST}$, iHS, CLR | Gibbs et al 2009 |
| | | | | New Zealand and USA | 27 | BHMC | 32,689 | Btau 4.0 | CLL | Stella et al 2010 |
| | | | | New Zealand and USA | 24 | 50K | 47,365 | UMD 3.1 | iHS, Rsb | Flori et al 2012 |
| | | | | New Zealand and USA | 24 | 50K | 44,057 | Btau 4.0 | $F_{ST}$, iHS | Gautier and Naves 2011 |
| | | | | New Zealand and USA | 24 | 800K | 281,994 | UMD 3.1 | Meta-SS | Utsunomiya et al 2013 |
| | | | | New Zealand and USA | 39 | 800K | 581,820 | UMD 3.1 | *di* ($F_{ST}$) | Xu et al 2014 |
| | | | | Scotland | 2918 | 50K | 52,942 | UMD 3.1 | Low MAF | Ramey et al 2013 |
| | | | | Scotland | 23 | AFFXB1P | 2,575,339 | UMD 3.1 | Low MAF | Ramey et al 2013 |
| | | | | USA | 586 | 50K | 45,632 | UMD_3.1 | iHS | Kim et al 2015b |
| | | | | South Africa | 31 | 50K | 45,657 | UMD_3.1 | FST, Low MAF | Makina et al 2015 |
| | | | | Ireland | 269 | 800K | 705,234 | UMD_3.1 | FST, iHS | Zhao et al 2015 |
| Angus-Hereford-Limousin | AHL | European | - | USA (Parallele) | 42 | 10K | 9,323 | Btau 3.1 | iHS, AFD | Hayes et al 2009 |

## S2 Table

| **Breed name** | **Code** | **Breed type** | **Land of Origin** | **Country of sampling** | **Samples** | **SNPchip** | **SNPs** | **Assembly** | **Selection tests** | **References** |
|---|---|---|---|---|---|---|---|---|---|---|
| Arsi, Ambo and Horro | AAH | African | Ethiopia | Ethiopia | 21 | 50K | 29,736 | UMD 3.1 | $F_{ST}$ | Edea et al 2014 |
| **Aubrac** | **AUB** | **European** | **France** | France | 22 | 50K | 44,057 | Btau 4.0 | $F_{ST}$, iHS | Gautier and Naves 2011 |
| | | | | France | 22 | 50K | 38,610 | UMD 3.1 | CSS | Randhawa et al 2014 |
| | | | | France | 20 | 50K | 35,320 | Btau 4.0 | BF | Gautier et al 2009 |
| Australian Friesian Sahiwal | AFS | Composite | Australia | Australia | 5 | 10K | 8,859 | Btau 4.0 | $F_{ST}$ | Barendse et al 2009 |
| **Australian Red** | **AUR** | **European** | **Australia** | Australia | 54 | 10K | 8,859 | Btau 4.0 | $F_{ST}$ | Barendse et al 2009 |
| | | | | Australia | 7 | 10K | 7,956 | Btau 4.0 | $F_{ST}$, EHH | Chan et al 2010 |
| **Baoule** | **BOL** | **African** | **Burkina Faso** | Burkina Faso | 29 | 50K | 38,610 | UMD 3.1 | CSS | Randhawa et al 2014 |
| | | | | Burkina Faso | 29 | 50K | 47,365 | UMD 3.1 | iHS, Rsb | Flori et al 2012 |
| | | | | Burkina Faso | 29 | 50K | 44,057 | Btau 4.0 | $F_{ST}$, iHS | Gautier and Naves 2011 |
| | | | | Burkina Faso | 29 | 50K | 35,320 | Btau 4.0 | BF | Gautier et al 2009 |
| | | | | Burkina Faso | 29 | 50K | 33,024 | UMD 3.1 | CSS | Randhawa et al 2015 |
| **Beefmaster** | **BFM** | **Composite** | **USA** | USA | 24 | BHMC | 32,470 | Btau 4.0 | $F_{ST}$ | Barendse et al 2009 |
| | | | | USA | 24 | BHMC | 37,470 | Btau 3.1 | $F_{ST}$, iHS, CLR | Gibbs et al 2009 |
| | | | | USA | 24 | BHMC | 32,689 | Btau 4.0 | CLL | Stella et al 2010 |
| **Belgian Blue** | **BLB** | **European** | **Belgium** | USA | 4 | 50K | 38,610 | UMD 3.1 | CSS | Randhawa et al 2014 |
| (Blanc-Bleu Belge) | | | | Belgium | 275 | 800K | 725,293 | UMD 3.1 | HMM-SFS | Druet et al 2013 |
| | | | | BHMC | 52 | 800K | 725,293 | UMD 3.1 | HMM-SFS | Druet et al 2013 |
| | | | | Germany | 31 | 50K | 47,651 | UMD 3.1 | XP-EHH | Rothammer et al 2013 |
| | | | | Ireland | 196 | 800K | 705,234 | UMD_3.1 | FST, iHS | Zhao et al 2015 |
| **Belmont Red** | **BLR** | **European** | **Australia** | Australia | 166 | 50K | 47,416 | Btau 4.0 | $F_{ST}$, iHS | Qanbari et al 2011 |
| | | | | Australia | 30 | 10K | 7,956 | Btau 4.0 | $F_{ST}$, EHH | Chan et al 2010 |
| | | | | Australia | 24 | 10K | 8,859 | Btau 4.0 | $F_{ST}$ | Barendse et al 2009 |
| Belted Galloway | BLG | European | Scotland | UK | 4 | 50K | 38,610 | UMD 3.1 | CSS | Randhawa et al 2014 |
| | | | | UK | 4 | 50K | 37,218 | UMD 3.1 | CSS | Randhawa et al 2015 |
| **Blonde dAquitaine** | **BAQ** | **European** | **France** | France | 30 | 50K | 35,554 | UMD 3.1 | HMM-SFS | Boitard and Rocha 2013 |
| | | | | USA | 5 | 50K | 38,610 | UMD 3.1 | CSS | Randhawa et al 2014 |
| | | | | USA | 5 | 50K | 37,218 | UMD 3.1 | CSS | Randhawa et al 2015 |
| Borana and Danakil | BAD | African | Ethiopia | Ethiopia | 26 | 50K | 29,736 | UMD 3.1 | $F_{ST}$ | Edea et al 2014 |
| **Borgou** (Ketuku) | **BGU** | **African** | **Benin** | Benin | 30 | 50K | 47,365 | UMD 3.1 | iHS, Rsb | Flori et al 2012 |
| | | | | Benin | 30 | 50K | 44,057 | Btau 4.0 | $F_{ST}$, iHS | Gautier and Naves 2011 |

**S2 Table**

| Breed name | Code | Breed type | Land of Origin | Country of sampling | Samples | SNPchip | SNPs | Assembly | Selection tests | References |
|---|---|---|---|---|---|---|---|---|---|---|
| | | | | Benin | 30 | 50K | 38,610 | UMD 3.1 | CSS | Randhawa et al 2014 |
| | | | | Parakou (Benin) | 45 | 50K | 35,320 | Btau 4.0 | BF | Gautier et al 2009 |
| | | | | Benin | 203 | 50K | 38,100 | UMD 3.1 | iHS, Rsb | Flori et al 2014 |
| **Brahman** | **BRM** | **Zebu** | **India** | Australia | 80 | 50K | 45,173 | Btau 4.0 | $F_{ST}$, iHS | Qanbari et al 2011 |
| | | | | Australia | 70 | 10K | 7,956 | Btau 4.0 | $F_{ST}$, EHH | Chan et al 2010 |
| | | | | Australia | 21 | 10K | 8,859 | Btau 4.0 | $F_{ST}$ | Barendse et al 2009 |
| | | | | Australia and USA | 25 | BHMC | 32,470 | Btau 4.0 | $F_{ST}$ | Barendse et al 2009 |
| | | | | Australia and USA | 25 | 50K | 47,365 | UMD 3.1 | iHS, Rsb | Flori et al 2012 |
| | | | | Australia and USA | 25 | BHMC | 37,470 | Btau 3.1 | $F_{ST}$, iHS, CLR | Gibbs et al 2009 |
| | | | | Australia and USA | 25 | BHMC | 32,689 | Btau 4.0 | CLL | Stella et al 2010 |
| | | | | Australia and USA | 25 | 50K | 44,057 | Btau 4.0 | $F_{ST}$, iHS | Gautier and Naves 2011 |
| | | | | Martinique | 25 | 50K | 44,057 | Btau 4.0 | $F_{ST}$, iHS | Gautier and Naves 2011 |
| | | | | USA | 99 | 50K | 52,942 | UMD 3.1 | Low MAF | Ramey et al 2013 |
| | | | | USA | 8 | AFFXB1P | 2,575,339 | UMD 3.1 | Low MAF | Ramey et al 2013 |
| | | | | USA | 30 | 800K | 581,820 | UMD 3.1 | *di* ($F_{ST}$) | Xu et al 2014 |
| **Braunvieh and** | **BNV** | **European** | **South Germany and** | Germany | 50 | 50K | 47,651 | UMD 3.1 | XP-EHH | Rothammer et al 2013 |
| **Original Braunvieh** | | | **Switzerland** | Germany | 35 | 50K | 47,651 | UMD 3.1 | XP-EHH | Rothammer et al 2013 |
| | | | | Switzerland | 142 | 50K | 52,942 | UMD 3.1 | Low MAF | Ramey et al 2013 |
| Bretonne Black Pied | BBP | European | France | France | 18 | 50K | 38,610 | UMD 3.1 | CSS | Randhawa et al 2014 |
| | | | | France | 18 | 50K | 37,218 | UMD 3.1 | CSS | Randhawa et al 2015 |
| **Brown Swiss** | **BSW** | **European** | **Switzerland** | Australia | 4 | 10K | 8,859 | Btau 4.0 | $F_{ST}$ | Barendse et al 2009 |
| | | | | Australia | 4 | 10K | 7,956 | Btau 4.0 | $F_{ST}$, EHH | Chan et al 2010 |
| | | | | Austria | 287 | 50K | 34,851 | Btau 4.0 | iHS and GWAS | Schwarzenbacher et al 2012 |
| | | | | Austria | 79 | 800K | 550,837 | UMD 3.1 | VarLD | Perez Obrien et al 2014 |
| | | | | Germany | 277 | 50K | 40,595 | Btau 4.0 | $F_{ST}$, iHS | Qanbari et al 2011 |
| | | | | Switzerland | 74 | 50K | 52,942 | UMD 3.1 | Low MAF | Ramey et al 2013 |
| | | | | USA | 44 | 800K | 281,994 | UMD 3.1 | Meta-SS | Utsunomiya et al 2013 |
| | | | | USA | 24 | 800K | 768,506 | UMD 3.1 | $F_{ST}$ | Porto-Neto et al 2013 |
| | | | | USA | 22 | 800K | 725,293 | UMD 3.1 | HMM-SFS | Druet et al 2013 |
| | | | | USA | 41 | 50K | 38,610 | UMD 3.1 | CSS | Randhawa et al 2014 |
| | | | | USA | 24 | BHMC | 32,470 | Btau 4.0 | $F_{ST}$ | Barendse et al 2009 |

**S2 Table**

| **Breed name** | **Code** | **Breed type** | **Land of Origin** | **Country of sampling** | **Samples** | **SNPchip** | **SNPs** | **Assembly** | **Selection tests** | **References** |
|---|---|---|---|---|---|---|---|---|---|---|
| | | | | USA | 24 | BHMC | 37,470 | Btau 3.1 | $F_{ST}$, iHS, CLR | Gibbs et al 2009 |
| | | | | USA | 24 | BHMC | 32,689 | Btau 4.0 | CLL | Stella et al 2010 |
| Brown Swiss cross | BSX | European | Crossbred | Australia | 5 | 10K | 8,859 | Btau 4.0 | $F_{ST}$ | Barendse et al 2009 |
| Brown Swiss Holstein | BXH | European | Crossbred | Australia | 26 | 10K | 8,859 | Btau 4.0 | $F_{ST}$ | Barendse et al 2009 |
| **Charolais** | **CHL** | **European** | **France** | Australia | 463 | 800K | 692,527 | UMD 3.1 | $F_{ST}$, iHS, HAPS | Kemper et al 2014 |
| | | | | France | 44 | 50K | 52,942 | UMD 3.1 | Low MAF | Ramey et al 2013 |
| | | | | France | 20 | 50K | 44,057 | Btau 4.0 | $F_{ST}$, iHS | Gautier and Naves 2011 |
| | | | | USA | 37 | 800K | 725,293 | UMD 3.1 | HMM-SFS | Druet et al 2013 |
| | | | | USA | 37 | 800K | 768,506 | UMD 3.1 | $F_{ST}$ | Porto-Neto et al 2013 |
| | | | | USA, UK | 55 | 50K | 38,610 | UMD 3.1 | CSS | Randhawa et al 2014 |
| | | | | USA, UK | 55 | 50K | 37,218 | UMD 3.1 | CSS | Randhawa et al 2015 |
| | | | | USA | 35 | 800K | 581,820 | UMD 3.1 | *di* ($F_{ST}$) | Xu et al 2014 |
| | | | | USA | 24 | BHMC | 32,470 | Btau 4.0 | $F_{ST}$ | Barendse et al 2009 |
| | | | | USA | 24 | BHMC | 37,470 | Btau 3.1 | $F_{ST}$, iHS, CLR | Gibbs et al 2009 |
| | | | | USA | 24 | BHMC | 32,689 | Btau 4.0 | CLL | Stella et al 2010 |
| | | | | Ireland | 710 | 800K | 705,234 | UMD_3.1 | FST, iHS | Zhao et al 2015 |
| Chianina | CHN | European | Italy | USA | 8 | 50K | 38,610 | UMD 3.1 | CSS | Randhawa et al 2014 |
| | | | | USA | 8 | 50K | 37,218 | UMD 3.1 | CSS | Randhawa et al 2015 |
| Creole | CRL | Composite | New World | Guadeloupe Island | 140 | 50K | 44,057 | Btau 4.0 | $F_{ST}$, iHS | Gautier and Naves 2011 |
| Devon | DEV | European | England | UK | 4 | 50K | 38,610 | UMD 3.1 | CSS | Randhawa et al 2014 |
| | | | | UK | 4 | 50K | 37,218 | UMD 3.1 | CSS | Randhawa et al 2015 |
| Dexter | DTR | European | Ireland | UK | 4 | 50K | 38,610 | UMD 3.1 | CSS | Randhawa et al 2014 |
| | | | | UK | 4 | 50K | 37,218 | UMD 3.1 | CSS | Randhawa et al 2015 |
| Drakensberger | DKR | African | South Africa | South Africa | 47 | 50K | 45,657 | UMD_3.1 | $F_{ST}$, Low MAF | Makina et al 2015 |
| East African Shorthorn Zebu | EASZ | Zebu | Africa | Kenya | 425 | 50K | 46,171 | UMD_3.1 | $F_{ST}$, iHS, Rsb | Bahbahani et al 2015 |
| **Finnish Ayrshire** | **FAR** | **European** | **Finland** | Finland | 10 | 50K | 38,610 | UMD 3.1 | CSS | Randhawa et al 2014 |
| | | | | Finland | 10 | 50K | 37,218 | UMD 3.1 | CSS | Randhawa et al 2015 |
| | | | | Scotland | 599 | 50K | 52,942 | UMD 3.1 | Low MAF | Ramey et al 2013 |
| **Fleckvieh (Simmental)** | **FKV** | **European** | **South Germany** | Germany | 43 | Sequencing | 15,182,131 | UMD 3.1 | iHS, CLR | Qanbari et al 2014 |
| | | | | Germany | 50 | 50K | 47,651 | UMD 3.1 | XP-EHH | Rothammer et al 2013 |
| French Red Pied Lowland | FRP | European | France | France | 22 | 50K | 38,610 | UMD 3.1 | CSS | Randhawa et al 2014 |

## S2 Table

| Breed name | Code | Breed type | Land of Origin | Country of sampling | Samples | SNPchip | SNPs | Assembly | Selection tests | References |
|---|---|---|---|---|---|---|---|---|---|---|
| **Galloway** | **GAL** | **European** | **Scotland** | Germany | 32 | 50K | 47,651 | UMD 3.1 | XP-EHH | Rothammer et al 2013 |
| | | | | UK | 4 | 50K | 38,610 | UMD 3.1 | CSS | Randhawa et al 2014 |
| | | | | UK | 4 | 50K | 37,2185 | UMD 3.1 | CSS | Randhawa et al 2015 |
| **Gascon** | **GAS** | **European** | **South West France** | France | 22 | 50K | 38,610 | UMD 3.1 | CSS | Randhawa et al 2014 |
| | | | | France | 22 | 50K | 44,057 | Btau 4.0 | $F_{ST}$, iHS | Gautier and Naves 2011 |
| Franken Gelbvieh | FGV | European | Central Germany | Germany | 50 | 50K | 47,651 | UMD 3.1 | XP-EHH | Rothammer et al 2013 |
| Gelbvieh | GBV | European | Germany | USA | 8 | 50K | 38,610 | UMD 3.1 | CSS | Randhawa et al 2014 |
| **Gir** | **GIR** | **Zebu** | **India** | Austria | 25 | 800K | 466,953 | UMD 3.1 | VarLD | Perez Obrien et al 2014 |
| | | | | Brazil | 24 | BHMC | 32,470 | Btau 4.0 | $F_{ST}$ | Barendse et al 2009 |
| | | | | Brazil | 24 | 50K | 44,057 | Btau 4.0 | $F_{ST}$, iHS | Gautier and Naves 2011 |
| | | | | Brazil | 24 | BHMC | 37,470 | Btau 3.1 | $F_{ST}$, iHS, CLR | Gibbs et al 2009 |
| | | | | Brazil | 24 | BHMC | 32,689 | Btau 4.0 | CLL | Stella et al 2010 |
| | | | | Brazil | 14 | Sequencing | 9,990,733 | UMD 3.1 | ZHp | Liao et al 2013 |
| | | | | Brazil | 50 | 800K | 768,506 | UMD 3.1 | $F_{ST}$ | Porto-Neto et al 2013 |
| | | | | Brazil | 24 | 50K | 47,365 | UMD 3.1 | iHS, Rsb | Flori et al 2012 |
| | | | | Brazil | 23 | 800K | 281,994 | UMD 3.1 | Meta-SS | Utsunomiya et al 2013 |
| **Guernsey** | **GNS** | **European** | **Channel Islands** | Australia | 4 | 10K | 8,859 | Btau 4.0 | $F_{ST}$ | Barendse et al 2009 |
| | | | | Australia | 4 | 10K | 7,956 | Btau 4.0 | $F_{ST}$, EHH | Chan et al 2010 |
| | | | | USA and UK | 21 | 800K | 725,293 | UMD 3.1 | HMM-SFS | Druet et al 2013 |
| | | | | USA and UK | 21 | 800K | 768,506 | UMD 3.1 | $F_{ST}$ | Porto-Neto et al 2013 |
| | | | | USA and UK | 21 | 50K | 38,610 | UMD 3.1 | CSS | Randhawa et al 2014 |
| | | | | USA and UK | 21 | BHMC | 32,470 | Btau 4.0 | $F_{ST}$ | Barendse et al 2009 |
| | | | | USA and UK | 21 | BHMC | 37,470 | Btau 3.1 | $F_{ST}$, iHS, CLR | Gibbs et al 2009 |
| | | | | USA and UK | 21 | BHMC | 32,689 | Btau 4.0 | CLL | Stella et al 2010 |
| Guernsey Holstein | GXH | European | Crossbred | Australia | 2 | 10K | 8,859 | Btau 4.0 | $F_{ST}$ | Barendse et al 2009 |
| Guzera | GZR | Zebu | Brazil/India | Brazil | 25 | 800K | 768,506 | UMD 3.1 | $F_{ST}$ | Porto-Neto et al 2013 |
| **Hanwoo** | **HNW** | **European** | **Korea** | Korea | 266 | 10K | 8344/4522 | Btau 3.1 | iES | Lim et al 2013 |
| | | | | Korea | 48 | 50K | 52,942 | UMD 3.1 | Low MAF | Ramey et al 2013 |
| | | | | Korea | 12 | Sequencing | 15,125,420 | UMD 3.1 | SFS (CLR), LD | Lee et al 2013 |
| | | | | Korea | 11 | Sequencing | 17,666,906 | UMD 3.1 | XP-EHH, XP-CLR | Lee et al 2014 |
| | | | | Korea | 11 | AFFXB1P | 2,575,339 | UMD 3.1 | Low MAF | Ramey et al 2013 |

**S2 Table**

| Breed name | Code | Breed type | Land of Origin | Country of sampling | Samples | SNPchip | SNPs | Assembly | Selection tests | References |
|---|---|---|---|---|---|---|---|---|---|---|
| | | | | NEW | 217 | 800K | 680,000 | UMD 3.1 | $F_{ST}$ | Porto-Neto et al 2014 |
| | | | | South Korea | 7 | 50K | 37,218 | UMD 3.1 | CSS | Randhawa et al 2015 |
| | | | | Korea | 10 | Sequencing | 17,936,399 | UMD_3.1 | ZHp | Choi et al 2015 |
| | | | | Korea | 547 | 50K | 35968 | Btau_4.0 | REHH | Li and Kim 2015 |
| **Hereford** | **HFD** | **European** | **England** | Australia | 744 | 800K | 692,527 | UMD 3.1 | $F_{ST}$, iHS, HAPS | Kemper et al 2014 |
| | | | | Australia | 158 | 50K | 45,322 | Btau 4.0 | $F_{ST}$, iHS | Qanbari et al 2011 |
| | | | | Australia | 34 | 10K | 7,956 | Btau 4.0 | $F_{ST}$, EHH | Chan et al 2010 |
| | | | | Australia | 28 | 10K | 8,859 | Btau 4.0 | $F_{ST}$ | Barendse et al 2009 |
| | | | | New Zealand and USA | 36 | 800K | 768,506 | UMD 3.1 | $F_{ST}$ | Porto-Neto et al 2013 |
| | | | | New Zealand and USA | 35 | 800K | 725,293 | UMD 3.1 | HMM-SFS | Druet et al 2013 |
| | | | | New Zealand and USA | 27 | BHMC | 32,470 | Btau 4.0 | $F_{ST}$ | Barendse et al 2009 |
| | | | | New Zealand and USA | 27 | BHMC | 37,470 | Btau 3.1 | $F_{ST}$, iHS, CLR | Gibbs et al 2009 |
| | | | | New Zealand and USA | 27 | BHMC | 32,689 | Btau 4.0 | CLL | Stella et al 2010 |
| | | | | New Zealand and USA | 21 | 50K | 44,057 | Btau 4.0 | $F_{ST}$, iHS | Gautier and Naves 2011 |
| | | | | New Zealand and USA | 21 | 50K | 47,365 | UMD 3.1 | iHS, Rsb | Flori et al 2012 |
| | | | | New Zealand and USA | 55 | 800K | 680,000 | UMD 3.1 | $F_{ST}$ | Porto-Neto et al 2014 |
| | | | | New Zealand and USA | 31 | 50K | 38,610 | UMD 3.1 | CSS | Randhawa et al 2014 |
| | | | | UK | 812 | 50K | 52,942 | UMD 3.1 | Low MAF | Ramey et al 2013 |
| | | | | Ireland | 234 | 800K | 705,234 | UMD_3.1 | $F_{ST}$, iHS | Zhao et al 2015 |
| **Jersey** | **JER** | **European** | **Channel Islands** | Australia | 5240 | 800K | 616,350 | UMD 3.1 | $F_{ST}$, iHS, HAPS | Kemper et al 2014 |
| | | | | Australia | 10 | 10K | 8,859 | Btau 4.0 | $F_{ST}$ | Barendse et al 2009 |
| | | | | Australia | 10 | 10K | 7,956 | Btau 4.0 | $F_{ST}$, EHH | Chan et al 2010 |
| | | | | France, New Zealand and USA | 49 | 50K | 38,610 | UMD 3.1 | CSS | Randhawa et al 2014 |
| | | | | France, New Zealand and USA | 49 | 50K | 37,218 | UMD 3.1 | CSS | Randhawa et al 2015 |
| | | | | Jersey, USA | 78 | 50K | 52,942 | UMD 3.1 | Low MAF | Ramey et al 2013 |
| | | | | New Zealand and USA | 39 | 800K | 768,506 | UMD 3.1 | $F_{ST}$ | Porto-Neto et al 2013 |
| | | | | New Zealand and USA | 38 | 800K | 725,293 | UMD 3.1 | HMM-SFS | Druet et al 2013 |
| | | | | New Zealand and USA | 28 | BHMC | 32,470 | Btau 4.0 | $F_{ST}$ | Barendse et al 2009 |
| | | | | New Zealand and USA | 28 | BHMC | 37,470 | Btau 3.1 | $F_{ST}$, iHS, CLR | Gibbs et al 2009 |
| | | | | New Zealand and USA | 28 | BHMC | 32,689 | Btau 4.0 | CLL | Stella et al 2010 |
| | | | | New Zealand, USA and Australia | 610 | 800K | 680,000 | UMD 3.1 | $F_{ST}$ | Porto-Neto et al 2014 |

**S2 Table**

| **Breed name** | **Code** | **Breed type** | **Land of Origin** | **Country of sampling** | **Samples** | **SNPchip** | **SNPs** | **Assembly** | **Selection tests** | **References** |
|---|---|---|---|---|---|---|---|---|---|---|
| | | | | USA | 1602 | 50K | 37,154 | UMD 3.1 | iHS, Rsb | Kim et al 2015 |
| Jersey cross | JRX | European | Crossbred | Australia | 4 | 10K | 8,859 | Btau 4.0 | $F_{ST}$ | Barendse et al 2009 |
| **Holstein** | **HOL** | **European** | **Netherlands** | Australia | 13501 | 800K | 616,350 | UMD 3.1 | $F_{ST}$, iHS, HAPS | Kemper et al 2014 |
| | | | | Australia | 384 | 10K | 9,323 | Btau 3.1 | iHS, AFD | Hayes et al 2009 |
| | | | | Australia | 384 | 10K | 9,323 | Btau 3.1 | H, DAFS | Maceachern et al 2009 |
| | | | | Australia | 94 | 50K | 52,150 | Btau 4.0 | AFD | Larkin et al 2012 |
| | | | | Australia | 52 | 10K | 8,859 | Btau 4.0 | $F_{ST}$ | Barendse et al 2009 |
| | | | | Australia | 52 | 10K | 7,956 | Btau 4.0 | $F_{ST}$, EHH | Chan et al 2010 |
| | | | | France | 1578 | 50K | 41,777 | Btau 4.0 | $F_{ST}$ | Flori et al 2009 |
| | | | | France | 30 | 50K | 47,365 | UMD 3.1 | iHS, Rsb | Flori et al 2012 |
| | | | | France | 30 | 50K | 44,057 | Btau 4.0 | $F_{ST}$, iHS | Gautier and Naves 2011 |
| | | | | France, New Zealand and USA | 80 | 50K | 38,610 | UMD 3.1 | CSS | Randhawa et al 2014 |
| | | | | Germany | 2091 | 50K | 40,595 | Btau 4.0 | $F_{ST}$, iHS | Qanbari et al 2011 |
| | | | | Germany | 810 | 50K | 40,854 | Btau 4.0 | EHH, REHH | Qanbari et al 2010 |
| | | | | Israel | 912 | 50K | 41,812 | Btau 4.0 | REHH | Glick et al 2012 |
| | | | | Korea | 10 | Sequencing | 17,666,906 | UMD 3.1 | XP-EHH, XP-CLR | Lee et al 2014 |
| | | | | Netherlands | 995 | 50K | 52,942 | UMD 3.1 | Low MAF | Ramey et al 2013 |
| | | | | New Zealand and USA | 63 | 800K | 768,506 | UMD 3.1 | $F_{ST}$ | Porto-Neto et al 2013 |
| | | | | New Zealand and USA | 60 | 800K | 725,293 | UMD 3.1 | HMM-SFS | Druet et al 2013 |
| | | | | New Zealand and USA | 53 | BHMC | 32,470 | Btau 4.0 | $F_{ST}$ | Barendse et al 2009 |
| | | | | New Zealand and USA | 53 | BHMC | 37,470 | Btau 3.1 | $F_{ST}$, iHS, CLR | Gibbs et al 2009 |
| | | | | New Zealand and USA | 53 | BHMC | 32,689 | Btau 4.0 | CLL | Stella et al 2010 |
| | | | | New Zealand and USA | 748 | 800K | 680,000 | UMD 3.1 | $F_{ST}$ | Porto-Neto et al 2014 |
| | | | | New Zealand and USA | 44 | 800K | 581,820 | UMD 3.1 | *di* ($F_{ST}$) | Xu et al 2014 |
| | | | | USA | 2087 | 50K | 41,951 | UMD 3.1 | iHS | Kim et al 2013 |
| | | | | USA (Parallele) | 19 | 10K | 9,323 | Btau 3.1 | iHS, AFD | Hayes et al 2009 |
| | | | | China | 2106 | 50K | 40,130 | Btau 4.0 | EHH, REHH | Pan et al 2013 |
| | | | | Italy | 861 | 50K | 29,848 | Btau 4.0 | $F_{ST}$ | Mancini et al 2014 |
| | | | | Japan | 50 | 50K | 40,635 | Btau 4.0 | SWAD | Hosokawa et al 2012 |
| | | | | Poland | 416 | 50K | 43,315 | UMD 3.1 | SWAD | Gurgul et al 2015a |
| | | | | South Africa | 29 | 50K | 45,657 | UMD_3.1 | $F_{ST}$, Low MAF | Makina et al 2015 |

**S2 Table**

| Breed name | Code | Breed type | Land of Origin | Country of sampling | Samples | SNPchip | SNPs | Assembly | Selection tests | References |
|---|---|---|---|---|---|---|---|---|---|---|
| | | | | Ireland | 719 | 800K | 705,234 | UMD_3.1 | $F_{ST}$, iHS | Zhao et al 2015 |
| Italian Holstein | HOL | | | Italy | 1132 | 50K | 44,271 | Btau 4.0 | REHH | Bomba et al 2015 |
| Red Holstein | HOL | European | North Germany | Germany | 50 | 50K | 47,651 | UMD 3.1 | XP-EHH | Rothammer et al 2013 |
| Holstein cross | HOX | European | Crossbred | Australia | 3 | 10K | 8,859 | Btau 4.0 | $F_{ST}$ | Barendse et al 2009 |
| Holstein Jersey | HXJ | European | Crossbred | Australia | 9 | 10K | 8,859 | Btau 4.0 | $F_{ST}$ | Barendse et al 2009 |
| **Illawarra Shorthorn** | **ILW** | **European** | **Australia** | Australia | 8 | 10K | 8,859 | Btau 4.0 | $F_{ST}$ | Barendse et al 2009 |
| | | | | Australia | 8 | 10K | 7,956 | Btau 4.0 | $F_{ST}$, EHH | Chan et al 2010 |
| Illyrian Mountain Buša | IMB | European | Albania | Germany | 43 | 50K | 47,651 | UMD 3.1 | XP-EHH | Rothammer et al 2013 |
| **Italian Brown** | **ITB** | **European** | **Italy** | Italy | 755 | 50K | 29,848 | Btau 4.0 | $F_{ST}$ | Mancini et al 2014 |
| | | | | Italy | 749 | 50K | 42,514 | Btau4.0 | $F_{ST}$ | Pintus et al 2013 |
| | | | | Italy | 514 | 50K | 44,271 | Btau 4.0 | REHH | Bomba et al 2015 |
| Italian Pezzata Rossa | IPR | European | Italy | Italy | 483 | 50K | 29,848 | Btau 4.0 | $F_{ST}$ | Mancini et al 2014 |
| Japanese Black | JBL | European | Japan | Japan | 50 | 50K | 40,635 | Btau 4.0 | SWAD | Hosokawa et al 2012 |
| Kenyan crossbred | KNC | Hybrid | Kenya | Kenya | 162 | 50K | Not given | UMD 3.1 | iHS | Kim and Rothschild 2014 |
| Kerry | KRY | European | Ireland | UK | 3 | 50K | 38,610 | UMD 3.1 | CSS | Randhawa et al 2014 |
| | | | | UK | 3 | 50K | 37,218 | UMD 3.1 | CSS | Randhawa et al 2015 |
| Korean | KOR | European | Korea | Korea | 71 | 50K | 11,799 | Btau 4.2 | CLL | Ryu and Lee 2014 |
| **Kuri** | **KUR** | **African** | **Chad** | Chad | 30 | 50K | 47,365 | UMD 3.1 | iHS, Rsb | Flori et al 2012 |
| | | | | Chad | 30 | 50K | 33,024 | UMD 3.1 | CSS | Randhawa et al 2015 |
| | | | | Islands of Lake Chad around Bol | 47 | 50K | 35,320 | Btau 4.0 | BF | Gautier et al 2009 |
| **Lagune** | **LGU** | **African** | **Benin** | Benin | 30 | 50K | 47,365 | UMD 3.1 | iHS, Rsb | Flori et al 2012 |
| | | | | Benin | 30 | 50K | 44,057 | Btau 4.0 | $F_{ST}$, iHS | Gautier and Naves 2011 |
| | | | | Benin | 30 | 50K | 38,610 | UMD 3.1 | CSS | Randhawa et al 2014 |
| | | | | Benin | 30 | 50K | 33,024 | UMD 3.1 | CSS | Randhawa et al 2015 |
| | | | | Porto Novo district (Benin) | 44 | 50K | 35,320 | Btau 4.0 | BF | Gautier et al 2009 |
| **Limousin** | **LMS** | **European** | **France** | Australia | 61 | 800K | 692,527 | UMD 3.1 | $F_{ST}$, iHS, HAPS | Kemper et al 2014 |
| | | | | France | 261 | 50K | 52,942 | UMD 3.1 | Low MAF | Ramey et al 2013 |
| | | | | USA and France | 50 | 800K | 725,293 | UMD 3.1 | HMM-SFS | Druet et al 2013 |
| | | | | USA and France | 47 | 800K | 768,506 | UMD 3.1 | $F_{ST}$ | Porto-Neto et al 2013 |
| | | | | USA and France | 35 | 50K | 38,610 | UMD 3.1 | CSS | Randhawa et al 2014 |
| | | | | USA and France | 42 | BHMC | 32,470 | Btau 4.0 | $F_{ST}$ | Barendse et al 2009 |

## S2 Table

| Breed name | Code | Breed type | Land of Origin | Country of sampling | Samples | SNPchip | SNPs | Assembly | Selection tests | References |
|---|---|---|---|---|---|---|---|---|---|---|
| | | | | USA and France | 42 | BHMC | 37,470 | Btau 3.1 | $F_{ST}$, iHS, CLR | Gibbs et al 2009 |
| | | | | USA and France | 42 | BHMC | 32,689 | Btau 4.0 | CLL | Stella et al 2010 |
| | | | | USA and France | 25 | 50K | 47,365 | UMD 3.1 | iHS, Rsb | Flori et al 2012 |
| | | | | USA and France | 25 | 50K | 44,057 | Btau 4.0 | $F_{ST}$, iHS | Gautier and Naves 2011 |
| | | | | Poland | 201 | 50K | 40378 | UMD_3.1 | REHH | Gurgul et al 2015b |
| | | | | Ireland | 730 | 800K | 705,234 | UMD_3.1 | $F_{ST}$, iHS | Zhao et al 2015 |
| Lincoln Red | LNR | European | UK | USA | 9 | 50K | 38,610 | UMD 3.1 | CSS | Randhawa et al 2014 |
| Longhorn | LGH | European | England | UK | 3 | 50K | 38,610 | UMD 3.1 | CSS | Randhawa et al 2014 |
| Maine-Anjou (Rouge-des-Pres) | MAJ | European | France | USA | 21 | 50K | 38,610 | UMD 3.1 | CSS | Randhawa et al 2014 |
| | | | | USA | 21 | 50K | 37,218 | UMD 3.1 | CSS | Randhawa et al 2015 |
| **Marchigiana** | **MCG** | **European** | **Italy** | Italy | 381 | 50K | 29,848 | Btau 4.0 | $F_{ST}$ | Mancini et al 2014 |
| | | | | Italy | 410 | 50K | 44,271 | Btau 4.0 | REHH | Bomba et al 2015 |
| | | | | USA | 5 | 50K | 38,610 | UMD 3.1 | CSS | Randhawa et al 2014 |
| | | | | USA | 5 | 50K | 37,218 | UMD 3.1 | CSS | Randhawa et al 2015 |
| **Montbeliarde (Simmental)** | **MON** | **European** | **Eastern France** | France | 584 | 50K | 41,777 | Btau 4.0 | $F_{ST}$ | Flori et al 2009 |
| | | | | France | 35 | 50K | 38,610 | UMD 3.1 | CSS | Randhawa et al 2014 |
| | | | | France | 35 | 50K | 37,218 | UMD 3.1 | CSS | Randhawa et al 2015 |
| | | | | France | 30 | 50K | 47,365 | UMD 3.1 | iHS, Rsb | Flori et al 2012 |
| | | | | France | 30 | 50K | 44,057 | Btau 4.0 | $F_{ST}$, iHS | Gautier and Naves 2011 |
| Murnau-Werdenfelser | MWF | European | South Germany | Germany | 46 | 50K | 47,651 | UMD 3.1 | XP-EHH | Rothammer et al 2013 |
| **Murray Grey** | **MRG** | **European** | **Australia** | Australia | 254 | 800K | 692,527 | UMD 3.1 | $F_{ST}$, iHS, HAPS | Kemper et al 2014 |
| | | | | Australia | 57 | 50K | 41,369 | Btau 4.0 | $F_{ST}$, iHS | Qanbari et al 2011 |
| | | | | Australia | 20 | 10K | 8,859 | Btau 4.0 | $F_{ST}$ | Barendse et al 2009 |
| | | | | Australia | 14 | 10K | 7,956 | Btau 4.0 | $F_{ST}$, EHH | Chan et al 2010 |
| | | | | Australia | 27 | 800K | 680,000 | UMD 3.1 | $F_{ST}$ | Porto-Neto et al 2014 |
| | | | | USA | 5 | 50K | 38,610 | UMD 3.1 | CSS | Randhawa et al 2014 |
| | | | | USA | 5 | 50K | 37,218 | UMD 3.1 | CSS | Randhawa et al 2015 |
| **NDama** | **NDM** | **African** | **West Africa** | Burkina Faso | 14 | 50K | 35,320 | Btau 4.0 | BF | Gautier et al 2009 |
| | | | | Burkina Faso | 25 | 50K | 21,034 | Btau 4.0 | XP-EHH | Noyes et al 2011 |
| | | | | Burkina Faso | 25 | 50K | 47,365 | UMD 3.1 | iHS, Rsb | Flori et al 2012 |
| | | | | Burkina Faso (South East) | 14 | 50K | 44,057 | Btau 4.0 | $F_{ST}$, iHS | Gautier and Naves 2011 |

**S2 Table**

| Breed name | Code | Breed type | Land of Origin | Country of sampling | Samples | SNPchip | SNPs | Assembly | Selection tests | References |
|---|---|---|---|---|---|---|---|---|---|---|
| | | | | Burkina Faso (South West) | 17 | 50K | 44,057 | Btau 4.0 | $F_{ST}$, iHS | Gautier and Naves 2011 |
| | | | | Burkina Faso (Samandeni herd) | 17 | 50K | 35,320 | Btau 4.0 | BF | Gautier et al 2009 |
| | | | | Burkina Faso, Guinea and Gambia | 61 | 50K | 38,610 | UMD 3.1 | CSS | Randhawa et al 2014 |
| | | | | Guinea | 25 | BHMC | 32,470 | Btau 4.0 | $F_{ST}$ | Barendse et al 2009 |
| | | | | Guinea | 25 | 50K | 44,057 | Btau 4.0 | $F_{ST}$, iHS | Gautier and Naves 2011 |
| | | | | Guinea | 25 | BHMC | 37,470 | Btau 3.1 | $F_{ST}$, iHS, CLR | Gibbs et al 2009 |
| | | | | Guinea | 25 | BHMC | 32,689 | Btau 4.0 | CLL | Stella et al 2010 |
| | | | | Guinea | 21 | 800K | 680,000 | UMD 3.1 | $F_{ST}$ | Porto-Neto et al 2014 |
| | | | | Guinea | 21 | 800K | 581,820 | UMD 3.1 | *di* ($F_{ST}$) | Xu et al 2014 |
| Nguni | NGN | African | Africa | South Africa | 54 | 50K | 45,657 | UMD_3.1 | $F_{ST}$, Low MAF | Makina et al 2015 |
| **Normande** | **NOR** | **European** | **North West France** | France | 641 | 50K | 41,777 | Btau 4.0 | $F_{ST}$ | Flori et al 2009 |
| | | | | France | 30 | 50K | 44,057 | Btau 4.0 | $F_{ST}$, iHS | Gautier and Naves 2011 |
| | | | | France | 30 | 50K | 38,610 | UMD 3.1 | CSS | Randhawa et al 2014 |
| | | | | France | 31 | 50K | 37,218 | UMD 3.1 | CSS | Randhawa et al 2015 |
| **Nellore** | **NLR** | **Zebu** | **India** | Austria | 100 | 800K | 448,407 | UMD 3.1 | VarLD | Perez Obrien et al 2014 |
| | | | | Brazil | 24 | BHMC | 32,470 | Btau 4.0 | $F_{ST}$ | Barendse et al 2009 |
| | | | | Brazil | 24 | BHMC | 37,470 | Btau 3.1 | $F_{ST}$, iHS, CLR | Gibbs et al 2009 |
| | | | | Brazil | 24 | BHMC | 32,689 | Btau 4.0 | CLL | Stella et al 2010 |
| | | | | Brazil | 21 | 50K | 44,057 | Btau 4.0 | $F_{ST}$, iHS | Gautier and Naves 2011 |
| | | | | Brazil | 91 | 800K | 768,506 | UMD 3.1 | $F_{ST}$ | Porto-Neto et al 2013 |
| | | | | Brazil | 29 | 800K | 680,000 | UMD 3.1 | $F_{ST}$ | Porto-Neto et al 2014 |
| | | | | Brazil | 21 | 50K | 47,365 | UMD 3.1 | iHS, Rsb | Flori et al 2012 |
| | | | | Brazil | 45 | 800K | 281,994 | UMD 3.1 | Meta-SS | Utsunomiya et al 2013 |
| | | | | Brazil | 789 | 800K | 560,565 | UMD 3.1 | REHH | Somavilla et al 2014 |
| **Norwegian Red** | **NWR** | **European** | **Norway** | Norway | 17 | 800K | 768,506 | UMD 3.1 | $F_{ST}$ | Porto-Neto et al 2013 |
| | | | | Norway | 25 | BHMC | 32,470 | Btau 4.0 | $F_{ST}$ | Barendse et al 2009 |
| | | | | Norway | 25 | BHMC | 37,470 | Btau 3.1 | $F_{ST}$, iHS, CLR | Gibbs et al 2009 |
| | | | | Norway | 25 | BHMC | 32,689 | Btau 4.0 | CLL | Stella et al 2010 |
| | | | | Norway | 21 | 50K | 38,610 | UMD 3.1 | CSS | Randhawa et al 2014 |
| **Oulmes Zaer** | **OLZ** | **African** | **Morocco** | Morocco | 26 | 50K | 47,365 | UMD 3.1 | iHS, Rsb | Flori et al 2012 |
| | | | | Morocco | 26 | 50K | 44,057 | Btau 4.0 | $F_{ST}$, iHS | Gautier and Naves 2011 |

**S2 Table**

| **Breed name** | **Code** | **Breed type** | **Land of Origin** | **Country of sampling** | **Samples** | **SNPchip** | **SNPs** | **Assembly** | **Selection tests** | **References** |
|---|---|---|---|---|---|---|---|---|---|---|
| | | | | Morocco | 26 | 50K | 38,610 | UMD 3.1 | CSS | Randhawa et al 2014 |
| | | | | Morocco | 26 | 50K | 33,024 | UMD 3.1 | CSS | Randhawa et al 2015 |
| | | | | North of Morocco | 40 | 50K | 35,320 | Btau 4.0 | BF | Gautier et al 2009 |
| **Parthenais (Maraichine)** | **MAR** | **European** | **North West France** | France | 19 | 50K | 38,610 | UMD 3.1 | CSS | Randhawa et al 2014 |
| | | | | France | 19 | 50K | 44,057 | Btau 4.0 | $F_{ST}$, iHS | Gautier and Naves 2011 |
| **Piedmontese** | **PMT** | **European** | **Italy** | Canada | 43 | 50K | 40,595 | Btau 4.0 | $F_{ST}$, iHS | Qanbari et al 2011 |
| | | | | Italy | 364 | 50K | 42,514 | Btau4.0 | $F_{ST}$ | Pintus et al 2013 |
| | | | | Italy | 317 | 50K | 29,848 | Btau 4.0 | $F_{ST}$ | Mancini et al 2014 |
| | | | | Italy | 364 | 50K | 44,271 | Btau 4.0 | REHH | Bomba et al 2015 |
| | | | | Italy | 24 | BHMC | 32,470 | Btau 4.0 | $F_{ST}$ | Barendse et al 2009 |
| | | | | Italy | 24 | BHMC | 37,470 | Btau 3.1 | $F_{ST}$, iHS, CLR | Gibbs et al 2009 |
| | | | | Italy | 24 | BHMC | 32,689 | Btau 4.0 | CLL | Stella et al 2010 |
| | | | | Italy | 21 | 800K | 725,293 | UMD 3.1 | HMM-SFS | Druet et al 2013 |
| | | | | Italy and USA | 26 | 50K | 38,610 | UMD 3.1 | CSS | Randhawa et al 2014 |
| | | | | Italy and USA | 26 | 50K | 37,218 | UMD 3.1 | CSS | Randhawa et al 2015 |
| Pinzgauer | PNG | European | Austria | USA | 5 | 50K | 38,610 | UMD 3.1 | CSS | Randhawa et al 2014 |
| | | | | Austria and Slovak | 40 | 50K | 30,538 | UMD 3.1 | $F_{ST}$, iHS | Kasarda et al 2015 |
| Polish Red | POR | European | Poland | Poland | 292 | 50K | 43,315 | UMD 3.1 | SWAD | Gurgul et al 2015 |
| **Red Angus** | **ANR** | **European** | **Scotland** | USA and Canada | 11 | 800K | 768,506 | UMD 3.1 | $F_{ST}$ | Porto-Neto et al 2013 |
| | | | | USA and Canada | 12 | BHMC | 32,470 | Btau 4.0 | $F_{ST}$ | Barendse et al 2009 |
| | | | | USA and Canada | 12 | BHMC | 37,470 | Btau 3.1 | $F_{ST}$, iHS, CLR | Gibbs et al 2009 |
| | | | | USA and Canada | 12 | BHMC | 32,689 | Btau 4.0 | CLL | Stella et al 2010 |
| | | | | USA and Canada | 15 | 50K | 38,610 | UMD 3.1 | CSS | Randhawa et al 2014 |
| Red Poll | RPL | European | UK | USA | 5 | 50K | 38,610 | UMD 3.1 | CSS | Randhawa et al 2014 |
| **Romagnola** | **RMG** | **European** | **Italy** | Italy | 24 | BHMC | 32,470 | Btau 4.0 | $F_{ST}$ | Barendse et al 2009 |
| | | | | Italy | 24 | BHMC | 37,470 | Btau 3.1 | $F_{ST}$, iHS, CLR | Gibbs et al 2009 |
| | | | | Italy | 24 | 50K | 38,610 | UMD 3.1 | CSS | Randhawa et al 2014 |
| | | | | Italy | 24 | 50K | 37,218 | UMD 3.1 | CSS | Randhawa et al 2015 |
| | | | | Italy | 24 | BHMC | 32,689 | Btau 4.0 | CLL | Stella et al 2010 |
| | | | | Italy | 21 | 800K | 725,293 | UMD 3.1 | HMM-SFS | Druet et al 2013 |
| Romosinuano | RMS | European | New World Spanish | New World Spanish | 8 | 50K | 38,610 | UMD 3.1 | CSS | Randhawa et al 2014 |

**S2 Table**

| Breed name | Code | Breed type | Land of Origin | Country of sampling | Samples | SNPchip | SNPs | Assembly | Selection tests | References |
|---|---|---|---|---|---|---|---|---|---|---|
| | | | | New World Spanish | 8 | 50K | 37,218 | UMD 3.1 | CSS | Randhawa et al 2015 |
| **Salers** | **SAL** | **European** | **France** | France | 22 | 50K | 47,365 | UMD 3.1 | iHS, Rsb | Flori et al 2012 |
| | | | | France | 72 | 50K | 52,942 | UMD 3.1 | Low MAF | Ramey et al 2013 |
| | | | | France | 22 | 50K | 44,057 | Btau 4.0 | $F_{ST}$, iHS | Gautier and Naves 2011 |
| | | | | France and USA | 27 | 50K | 38,610 | UMD 3.1 | CSS | Randhawa et al 2014 |
| **Santa Gertrudis** | **SGT** | **Composite** | **USA** | Australia | 126 | 50K | 46,809 | Btau 4.0 | $F_{ST}$, iHS | Qanbari et al 2011 |
| | | | | Australia | 28 | 10K | 8,859 | Btau 4.0 | $F_{ST}$ | Barendse et al 2009 |
| | | | | Australia | 24 | 10K | 7,956 | Btau 4.0 | $F_{ST}$, EHH | Chan et al 2010 |
| | | | | Australia | 24 | 50K | 47,365 | UMD 3.1 | iHS, Rsb | Flori et al 2012 |
| | | | | USA | 24 | BHMC | 32,470 | Btau 4.0 | $F_{ST}$ | Barendse et al 2009 |
| | | | | USA | 24 | BHMC | 37,470 | Btau 3.1 | $F_{ST}$, iHS, CLR | Gibbs et al 2009 |
| | | | | USA | 24 | BHMC | 32,689 | Btau 4.0 | CLL | Stella et al 2010 |
| | | | | USA | 24 | 50K | 44,057 | Btau 4.0 | $F_{ST}$, iHS | Gautier and Naves 2011 |
| Scottish Highland | SCH | European | Scotland | UK and USA | 9 | 50K | 38,610 | UMD 3.1 | CSS | Randhawa et al 2014 |
| | | | | UK and USA | 9 | 50K | 37,218 | UMD 3.1 | CSS | Randhawa et al 2015 |
| Senepol | SEN | Composite | Caribbean Islands | Venezuela | 147 | 50K | 47,365 | UMD 3.1 | iHS, Rsb | Flori et al 2012 |
| **Sheko** | **SKO** | **African** | **Ethiopia** | Africa | 20 | 50K | 21,034 | Btau 4.0 | XP-EHH | Noyes et al 2011 |
| | | | | Ethiopia | 20 | BHMC | 32,470 | Btau 4.0 | $F_{ST}$ | Barendse et al 2009 |
| | | | | Ethiopia | 20 | BHMC | 37,470 | Btau 3.1 | $F_{ST}$, iHS, CLR | Gibbs et al 2009 |
| | | | | Ethiopia | 20 | 50K | 38,610 | UMD 3.1 | CSS | Randhawa et al 2014 |
| | | | | Ethiopia | 20 | BHMC | 32,689 | Btau 4.0 | CLL | Stella et al 2010 |
| **Shorthorn** | **SHN** | **European** | **England** | Australia | 868 | 800K | 692,527 | UMD 3.1 | $F_{ST}$, iHS, HAPS | Kemper et al 2014 |
| | | | | Australia | 81 | 50K | 42,280 | Btau 4.0 | $F_{ST}$, iHS | Qanbari et al 2011 |
| | | | | Australia | 54 | 800K | 680,000 | UMD 3.1 | $F_{ST}$ | Porto-Neto et al 2014 |
| | | | | Australia | 27 | 10K | 8,859 | Btau 4.0 | $F_{ST}$ | Barendse et al 2009 |
| | | | | Australia | 18 | 10K | 7,956 | Btau 4.0 | $F_{ST}$, EHH | Chan et al 2010 |
| | | | | UK | 108 | 50K | 52,942 | UMD 3.1 | Low MAF | Ramey et al 2013 |
| | | | | USA | 10 | 50K | 38,610 | UMD 3.1 | CSS | Randhawa et al 2014 |
| **Simmental (Pezzata Rossa)** | **SIM** | **European** | **Switzerland** | China | 942 | 50K | 39,094 | UMD 3.1 | EHH | Fan et al 2014 |
| | | | | Germany | 462 | 50K | 37,976; 40,595 | Btau 4.0 | $F_{ST}$, iHS | Qanbari et al 2011 |
| | | | | Switzerland | 123 | 50K | 52,942 | UMD 3.1 | Low MAF | Ramey et al 2013 |

**S2 Table**

| Breed name | Code | Breed type | Land of Origin | Country of sampling | Samples | SNPchip | SNPs | Assembly | Selection tests | References |
|---|---|---|---|---|---|---|---|---|---|---|
| | | | | Switzerland | 6 | AFFXB1P | 2,575,339 | UMD 3.1 | Low MAF | Ramey et al 2013 |
| | | | | USA | 10 | 50K | 38,610 | UMD 3.1 | CSS | Randhawa et al 2014 |
| | | | | USA | 10 | 50K | 37,218 | UMD 3.1 | CSS | Randhawa et al 2015 |
| | | | | Italy | 393 | 50K | 44,271 | Btau 4.0 | REHH | Bomba et al 2015 |
| | | | | Ireland | 264 | 800K | 705,234 | UMD_3.1 | $F_{ST}$, iHS | Zhao et al 2015 |
| **Somba** | **SMB** | **African** | **Togo** | Nadoba and Boukoumbe (Benin) | 44 | 50K | 35,320 | Btau 4.0 | BF | Gautier et al 2009 |
| | | | | Togo | 30 | 50K | 47,365 | UMD 3.1 | iHS, Rsb | Flori et al 2012 |
| | | | | Togo | 30 | 50K | 44,057 | Btau 4.0 | $F_{ST}$, iHS | Gautier and Naves 2011 |
| | | | | Togo | 30 | 50K | 38,610 | UMD 3.1 | CSS | Randhawa et al 2014 |
| | | | | Togo | 30 | 50K | 33,024 | UMD 3.1 | CSS | Randhawa et al 2015 |
| South Devon | SDV | European | England | UK | 4 | 50K | 38,610 | UMD 3.1 | CSS | Randhawa et al 2014 |
| | | | | UK | 4 | 50K | 37,218 | UMD 3.1 | CSS | Randhawa et al 2015 |
| Sussex | SSX | European | England | UK | 4 | 50K | 38,610 | UMD 3.1 | CSS | Randhawa et al 2014 |
| Tarine (Tarentaise) | TRN | European | France | France and USA | 23 | 50K | 38,610 | UMD 3.1 | CSS | Randhawa et al 2014 |
| Texas Longhorn | TLH | European | New World Spanish | New World Spanish | 10 | 50K | 38,610 | UMD 3.1 | CSS | Randhawa et al 2014 |
| Vosgienne (Vosges) | VSG | European | France | France | 19 | 50K | 38,610 | UMD 3.1 | CSS | Randhawa et al 2014 |
| Wagyu | WAG | European | Japan | Japan | 10 | AFFXB1P | 2,575,339 | UMD 3.1 | Low MAF | Ramey et al 2013 |
| Welsh Black | WLB | European | England | UK | 2 | 50K | 38,610 | UMD 3.1 | CSS | Randhawa et al 2014 |
| White Fulani Zebu /Suanese | WFZ | African | Benin | Malanville (Benin) | 43 | 50K | 35,320 | Btau 4.0 | BF | Gautier et al 2009 |
| White Park | WPK | European | England | UK | 4 | 50K | 38,610 | UMD 3.1 | CSS | Randhawa et al 2014 |
| Yanbian | YBN | European | Korea | Korea | 10 | Sequencing | 17,936,399 | UMD_3.1 | ZHp | Choi et al 2015 |
| Zebu Choa | ZCO | African | Chad | Bol (Chad) | 59 | 50K | 35,320 | Btau 4.0 | BF | Gautier et al 2009 |
| **Zebu Madagascar** | **ZMG** | **African** | **Madagascar** | Madagascar | 30 | 50K | 47,365 | UMD 3.1 | iHS, Rsb | Flori et al 2012 |
| | | | | Madagascar Island | 35 | 50K | 35,320 | Btau 4.0 | BF | Gautier et al 2009 |
| Repeats / crossbred | XXX | European | Many breeds | Australia | 10 | 10K | 8,859 | Btau 4.0 | $F_{ST}$ | Barendse et al 2009 |
