# Supplementary material for: A Meta-Assembly of Selection Signatures in Cattle
Source: PLoS One. 2016 Apr 5;11(4):e0153013. doi: 10.1371/journal.pone.0153013 (PMC4821596; doi:10.1371/journal.pone.0153013)
Supplement: S3 Table — (PDF) [file pone.0153013.s003.pdf]

S3 Table. List of cattle breeds categorized for the number of available results from genome-wide scans of selection signatures across different studies. The results for the breeds are available for individual breed-wise and group-wise (multiple breeds' results combined) in various studies. Some of the breeds were used in the reference panel only. Number of (N) selection signatures was compiled from studies available with Breed-wise results only.

**No.**	**Breed name**	**N selection signatures**	**Breed-wise studies**	**Group-wise studies**	**Used as reference**	**Total Studies**
1	Holstein	2090	**22**	7	4	**33**
2	Angus	986	**14**	7	2	**23**
3	Simmental	964	**9**	3	3	**15**
4	Brown Swiss	719	**8**	5	0	**13**
5	Hereford	676	**7**	6	2	**15**
6	Charolais	373	**7**	3	2	**12**
7	Limousin	840	**7**	3	2	**12**
8	Hanwoo	283	**7**	1	1	**9**
9	Jersey	624	**6**	7	1	**14**
10	Nellore	476	**6**	2	3	**11**
11	Brahman	138	**5**	5	2	**12**
12	Piedmontese	320	**5**	5	1	**11**
13	Gir	459	**5**	2	2	**9**
14	Belgian Blue	641	**4**	1	0	**5**
15	NDama	183	**3**	7	5	**15**
16	Guernsey	584	**3**	5	0	**8**
17	Shorthorn	38	**3**	4	0	**7**
18	Braunvieh	517	**3**	0	0	**3**

S3 Table

No.	Breed name	N selection signatures	Breed-wise studies	Group-wise studies	Used as reference	Total Studies
19	Murray Grey	41	**2**	5	0	**7**
20	Santa Gertrudis	27	**2**	3	2	**7**
21	Romagnola	82	**2**	3	1	**6**
22	Marchigiana	137	2	3	0	**5**
23	Norwegian Red	473	**2**	3	0	**5**
24	Sheko	12	**2**	2	1	**5**
25	Italian Brown	294	**2**	1	0	**3**
26	Red Angus	12	1	4	0	**5**
27	Borgou	49	1	3	1	**5**
28	Normande	24	1	2	1	**4**
29	Belmont Red	9	1	2	0	**3**
30	Blonde dAquitaine	3	1	2	0	**3**
31	Finnish Ayrshire	1	1	2	0	**3**
32	Galloway	290	1	2	0	**3**
33	Salers	4	1	1	2	**4**
34	Beef Master	15	1	1	1	**3**
35	Pinzgauer	13	1	1	0	**2**
36	Franken Gelbvieh	268	1	1	0	**2**
37	Anatolian Black	83	1	0	0	**1**
38	Creole	31	1	0	0	**1**
39	Guzera	79	1	0	0	**1**
40	Illyrian Mountain Busa	53	1	0	0	**1**

S3 Table

No.	Breed name	N selection signatures	Breed-wise studies	Group-wise studies	Used as reference	Total Studies
41	Japanese Black	11	1	0	0	**1**
42	Murnau-Werdenfelser	395	1	0	0	**1**
43	Senepol	7	1	0	0	**1**
44	Wagyu	34	1	0	0	**1**
45	Kenyan crossbred	7	1	0	0	**1**
46	Korean	28	1	0	0	**1**
47	Polish Red	19	1	0	0	**1**
48	Afrikaner	13	1	0	0	**1**
49	Bonsmara	12	1	0	0	**1**
50	Drakensberger	10	1	0	0	**1**
51	East African Shorthorn Zebu	21	1	0	0	**1**
52	Nguni	13	1	0	0	**1**
53	Yanbian	174	1	0	0	**1**
54	Baoule	-	0	4	2	**6**
55	Lagune	-	0	4	2	**6**
56	Somba	-	0	4	2	**6**
57	Oulmes Zaer	-	0	4	1	**5**
58	Kuri	-	0	2	1	**3**
59	Australian Red	-	0	2	0	**2**
60	Belted Galloway	-	0	2	0	**2**
61	Bretonne Black Pied	-	0	2	0	**2**
62	Chianina	-	0	2	0	**2**

S3 Table

No.	Breed name	N selection signatures	Breed-wise studies	Group-wise studies	Used as reference	Total Studies
63	Devon	-	0	2	0	**2**
64	Illawarra Shorthorn	-	0	2	0	**2**
65	Kerry	-	0	2	0	**2**
66	Maine-Anjou	-	0	2	0	**2**
67	Romosinuano	-	0	2	0	**2**
68	Scottish Highland	-	0	2	0	**2**
69	South Devon	-	0	2	0	**2**
70	Zebu Madagascar	-	0	1	2	**3**
71	Aubrac	-	0	1	2	**3**
72	White Fulani	-	0	1	1	**2**
73	Gascon	-	0	1	1	**2**
74	Maraichine	-	0	1	1	**2**
75	Ambo	-	0	1	0	**1**
76	Arsi	-	0	1	0	**1**
77	Australian Friesian Sahiwal	-	0	1	0	**1**
78	Dexter	-	0	1	0	**1**
79	French Red Pied Lowland	-	0	1	0	**1**
80	Horro	-	0	1	0	**1**
81	Lincoln Red	-	0	1	0	**1**
82	Longhorn	-	0	1	0	**1**
83	Red Poll	-	0	1	0	**1**
84	Sussex	-	0	1	0	**1**

S3 Table

No.	Breed name	N selection signatures	Breed-wise studies	Group-wise studies	Used as reference	Total Studies
85	Tarentaise	-	0	1	0	**1**
86	Texas Longhorn	-	0	1	0	**1**
87	Vosgienne	-	0	1	0	**1**
88	Welsh Black	-	0	1	0	**1**
89	White Park	-	0	1	0	**1**
90	Zebu Choa	-	0	1	0	**1**
91	Borana	-	0	0	1	**1**
92	Bororo Zebu	-	0	0	1	**1**
93	Danakil	-	0	0	1	**1**
