# Supplementary material for: A Meta-Assembly of Selection Signatures in Cattle
Source: PLoS One. 2016 Apr 5;11(4):e0153013. doi: 10.1371/journal.pone.0153013 (PMC4821596; doi:10.1371/journal.pone.0153013)
Supplement: S1 Fig — Bovine population (A), milk production (B), beef production (C) and hide production (D) in various top 20 countries of the World in 2010–11 (pie charts) and population trends in the past 50 years (trend lines). (PDF) [file pone.0153013.s008.pdf]

S1 Fig

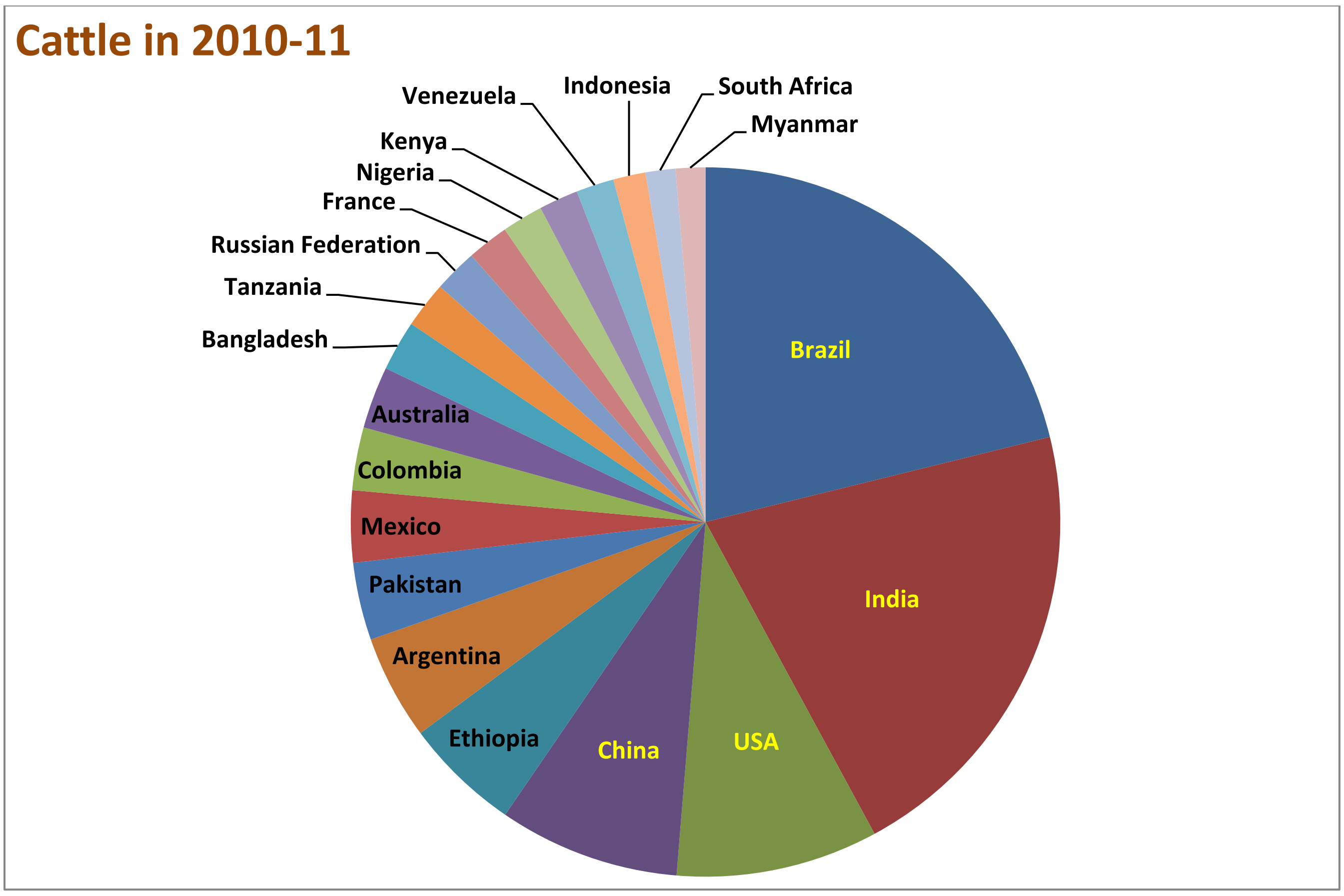

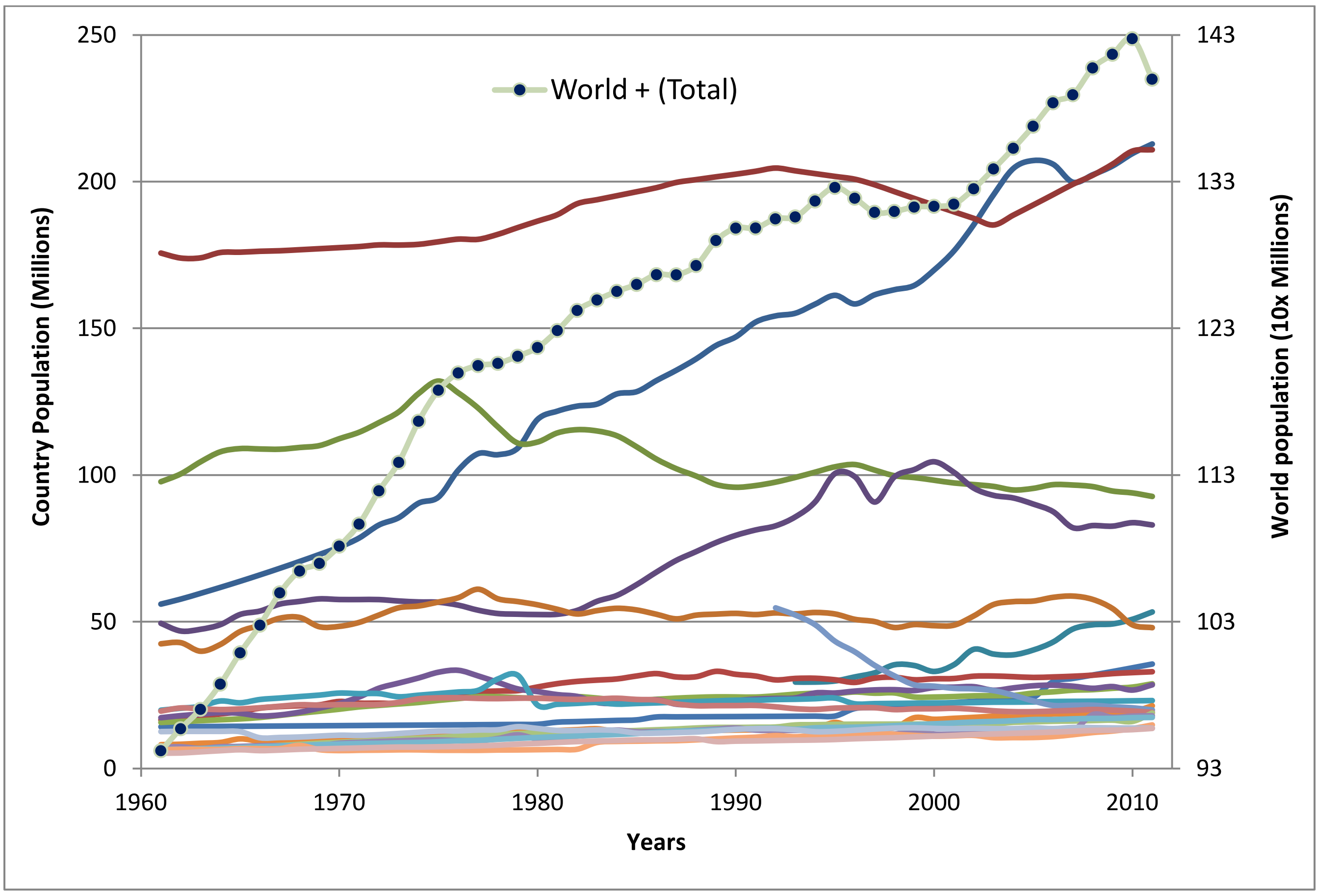

A. Bovine population in top 20 countries (Brazil to Myanmar) of the World in 2010-11 (pie chart) and population trends in the past 50 years (trend lines). Worldwide cattle population has increased from 930 to 1430 million heads (~ 50%) during the past 5 decades.

(Source: FAOSTAT (http://faostat.fao.org/site/573/DesktopDefault.aspx?PageID=573#ancor Updated: 16 January 2013, Accessed: 08 July 2013)

S1 Fig

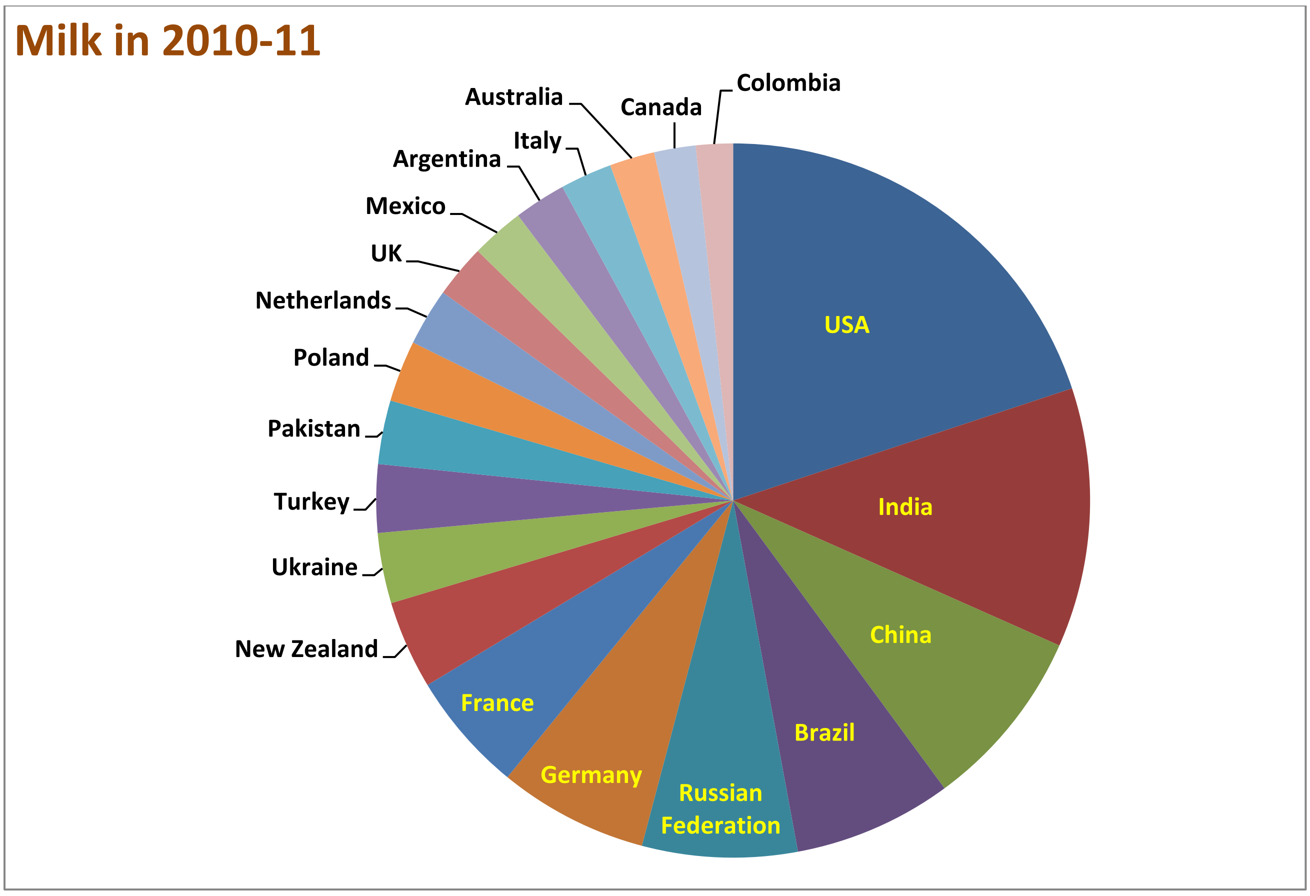

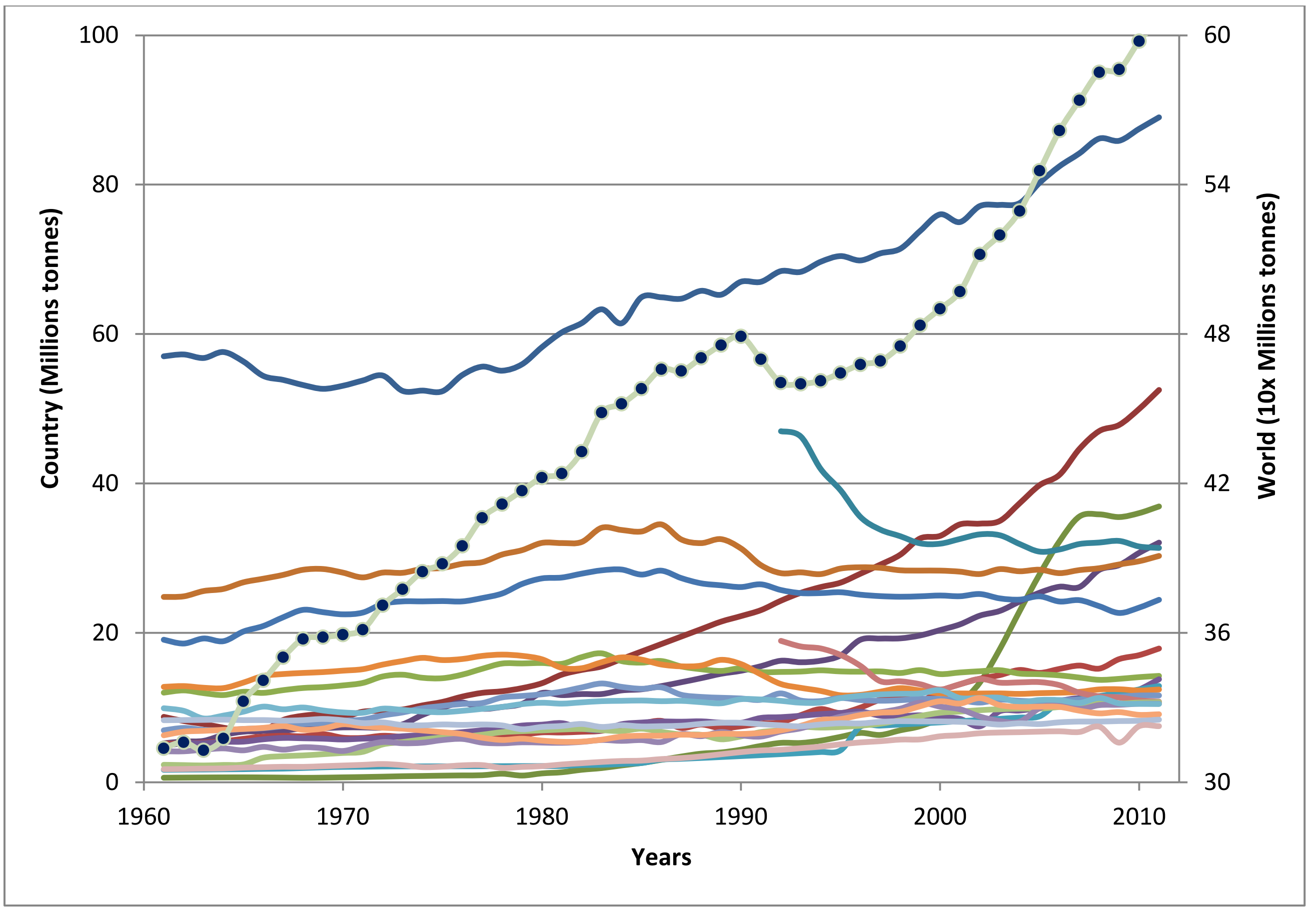

B. Bovine milk production in top 20 countries (USA to Colombia) of the World in 2010-11 (pie chart) and population trends in the past 50 years (trend lines). Worldwide cattle milk production has increased from ~ 300 to 600 million tonnes (~ 100%) during the past 5 decades.

(Source: **FAOSTAT** (http://faostat.fao.org/site/569/DesktopDefault.aspx?PageID=569#ancor Updated: 16 January 2013, Accessed: 08 July 2013)

S1 Fig

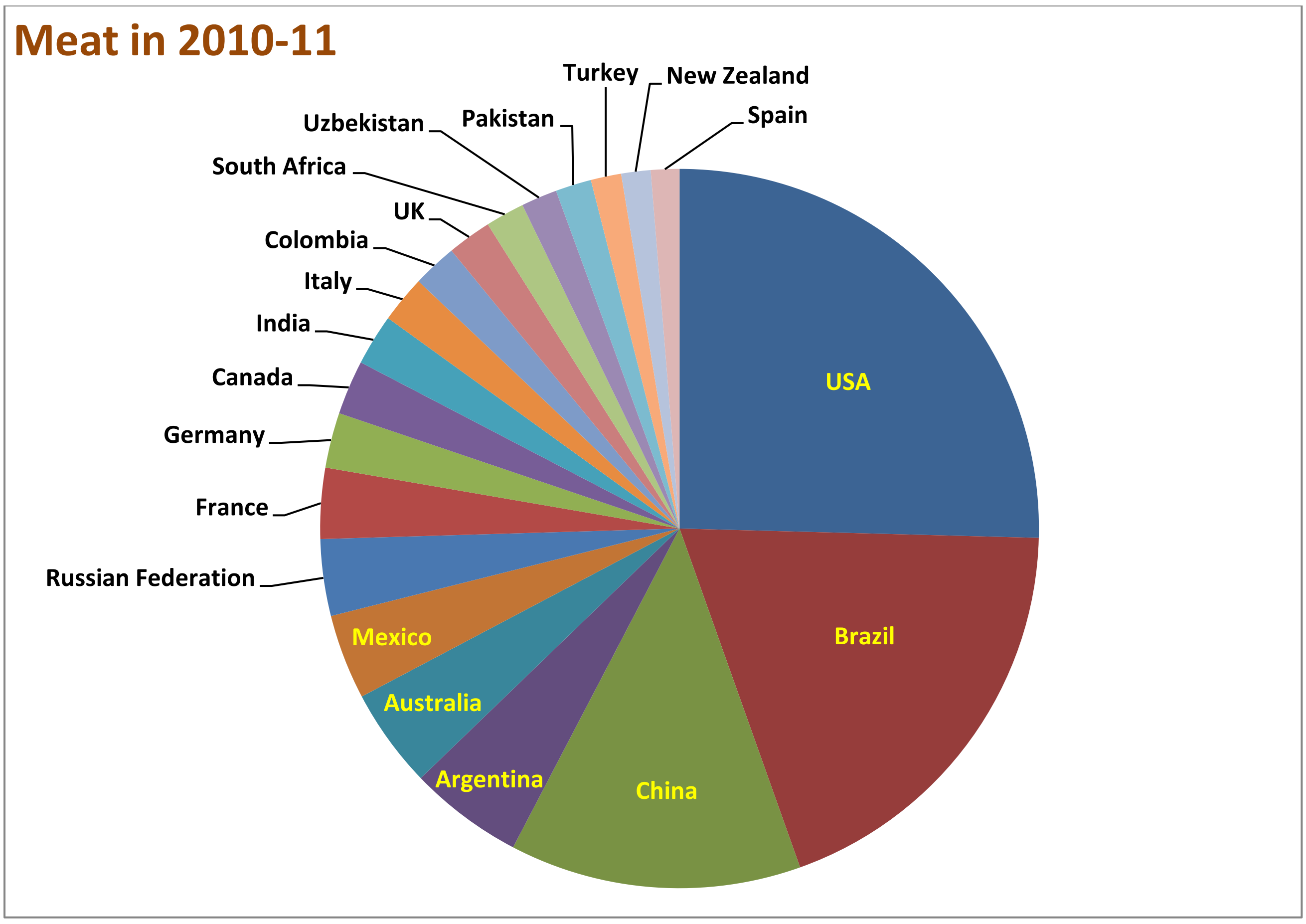

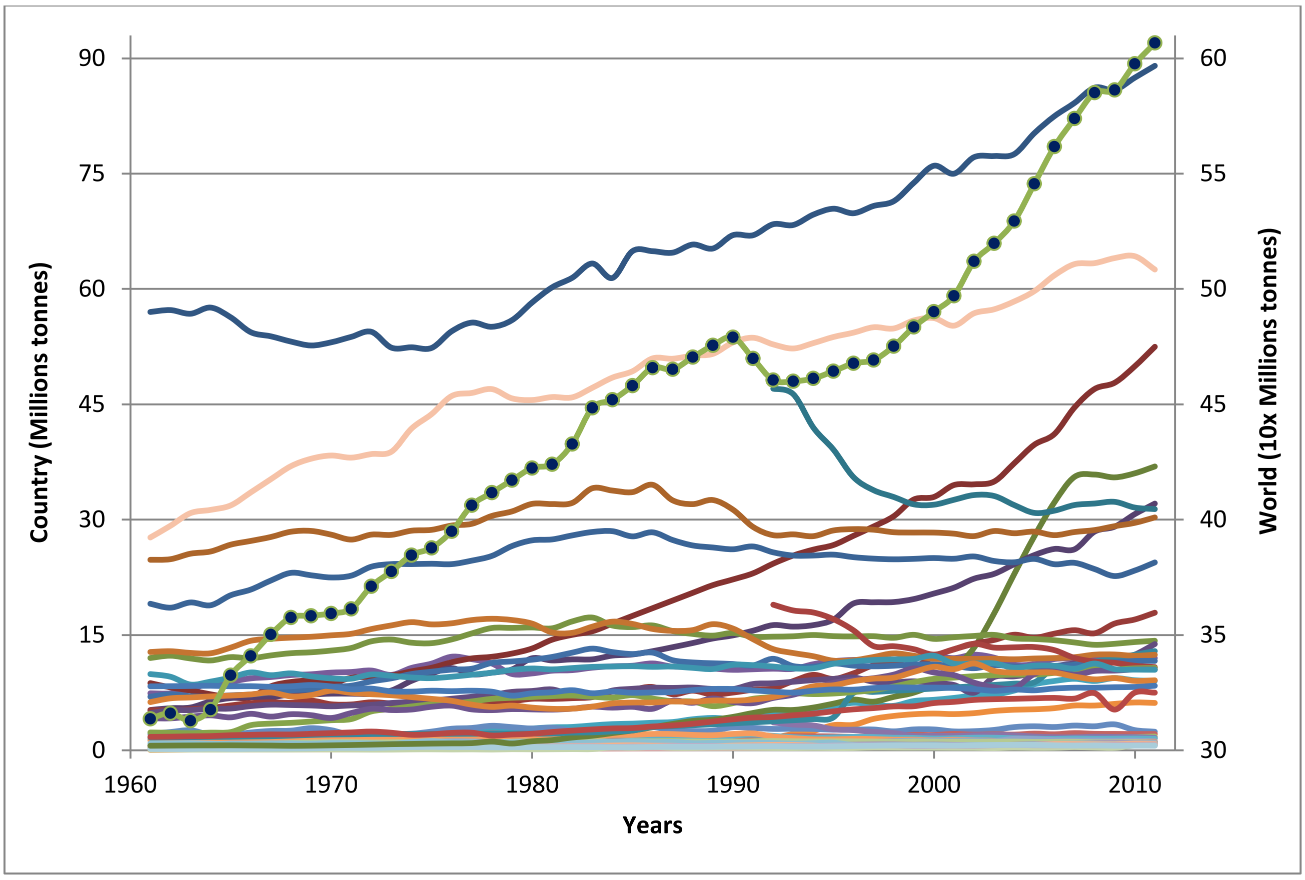

C. Bovine beef production in top 20 countries (USA to Spain) of the World in 2010-11 (pie chart) and population trends in the past 50 years (trend lines). Worldwide cattle meat (beef) production has increased from ~ 300 to 600 million tonnes (~ 100%) during the past 5 decades.

(Source: **FAOSTAT** (http://faostat.fao.org/site/569/DesktopDefault.aspx?PageID=569#ancor Updated: 16 January 2013, Accessed: 08 July 2013)

S1 Fig

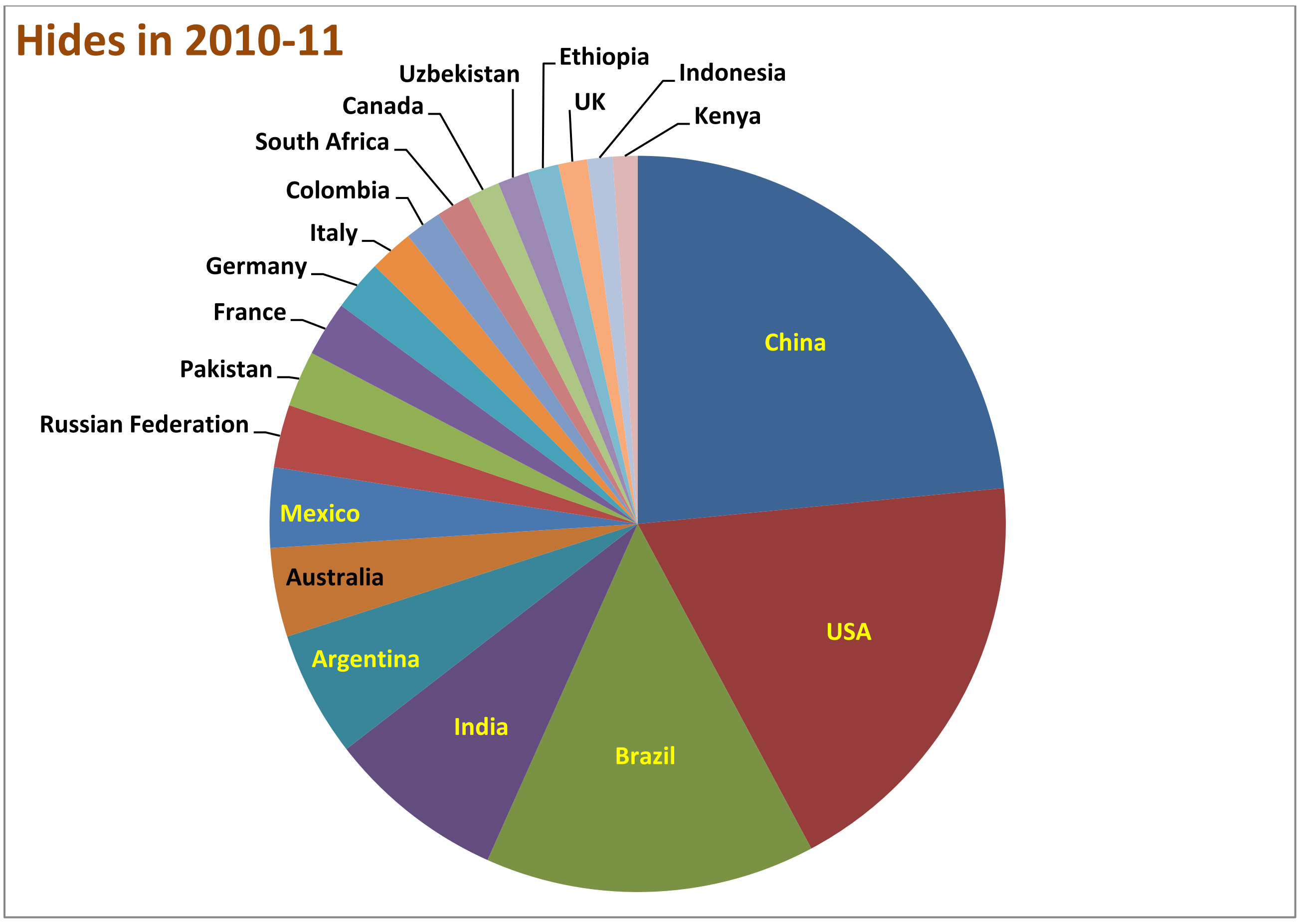

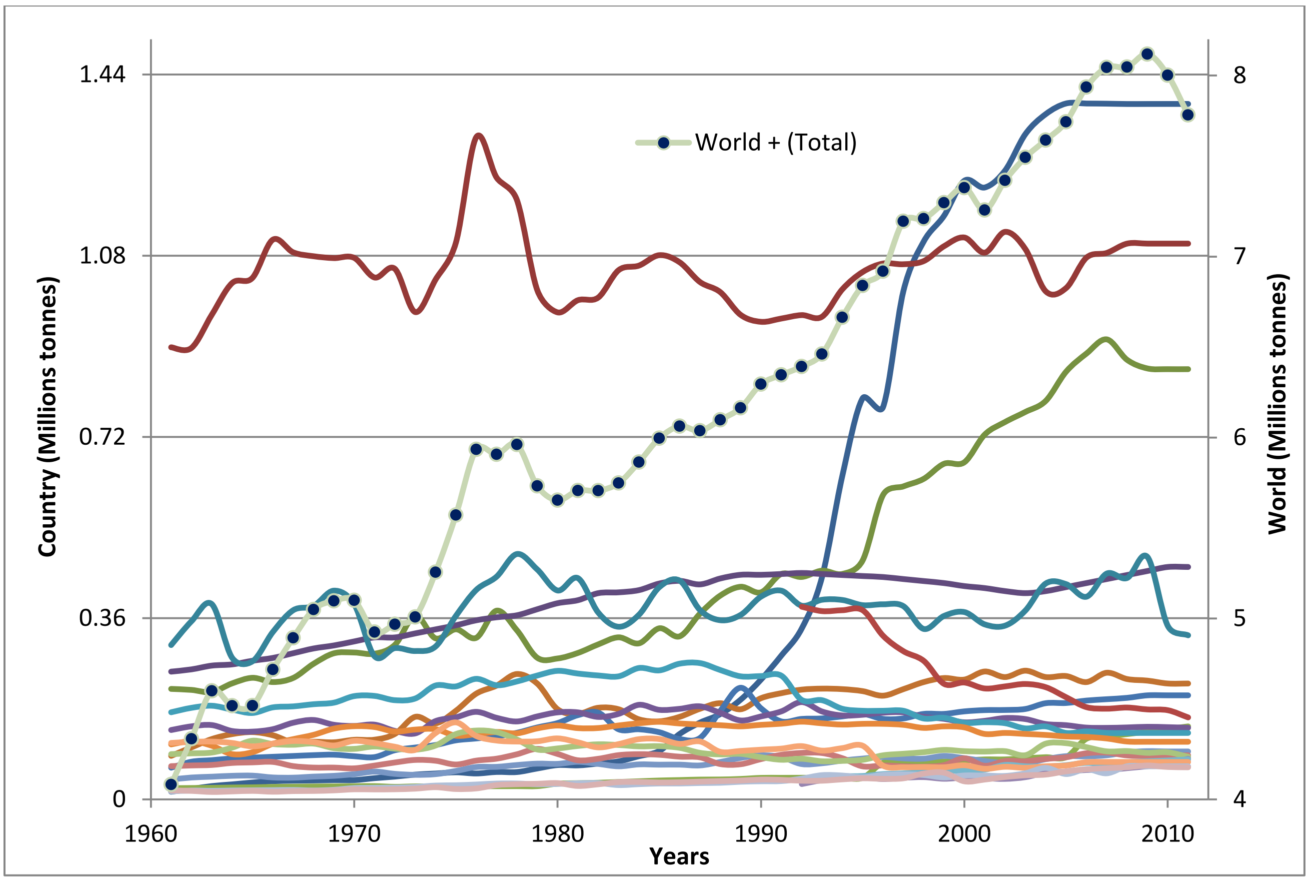

D. Bovine Hide Production (Tonnes) in top 20 countries (China to Kenya) of the World in 2010-11 (pie chart) and population trends in the past 50 years (trend lines). Worldwide cattle hide production has increased from ~ 4 to ~8 million tonnes (~ 100%) during the past 5 decades.

(Source: **FAOSTAT** (http://faostat.fao.org/site/569/DesktopDefault.aspx?PageID=569#ancor Updated: 16 January 2013, Accessed: 08 July 2013)
