# Supplementary material for: A Meta-Assembly of Selection Signatures in Cattle
Source: PLoS One. 2016 Apr 5;11(4):e0153013. doi: 10.1371/journal.pone.0153013 (PMC4821596; doi:10.1371/journal.pone.0153013)
Supplement: S2 Fig — (PDF) [file pone.0153013.s009.pdf]

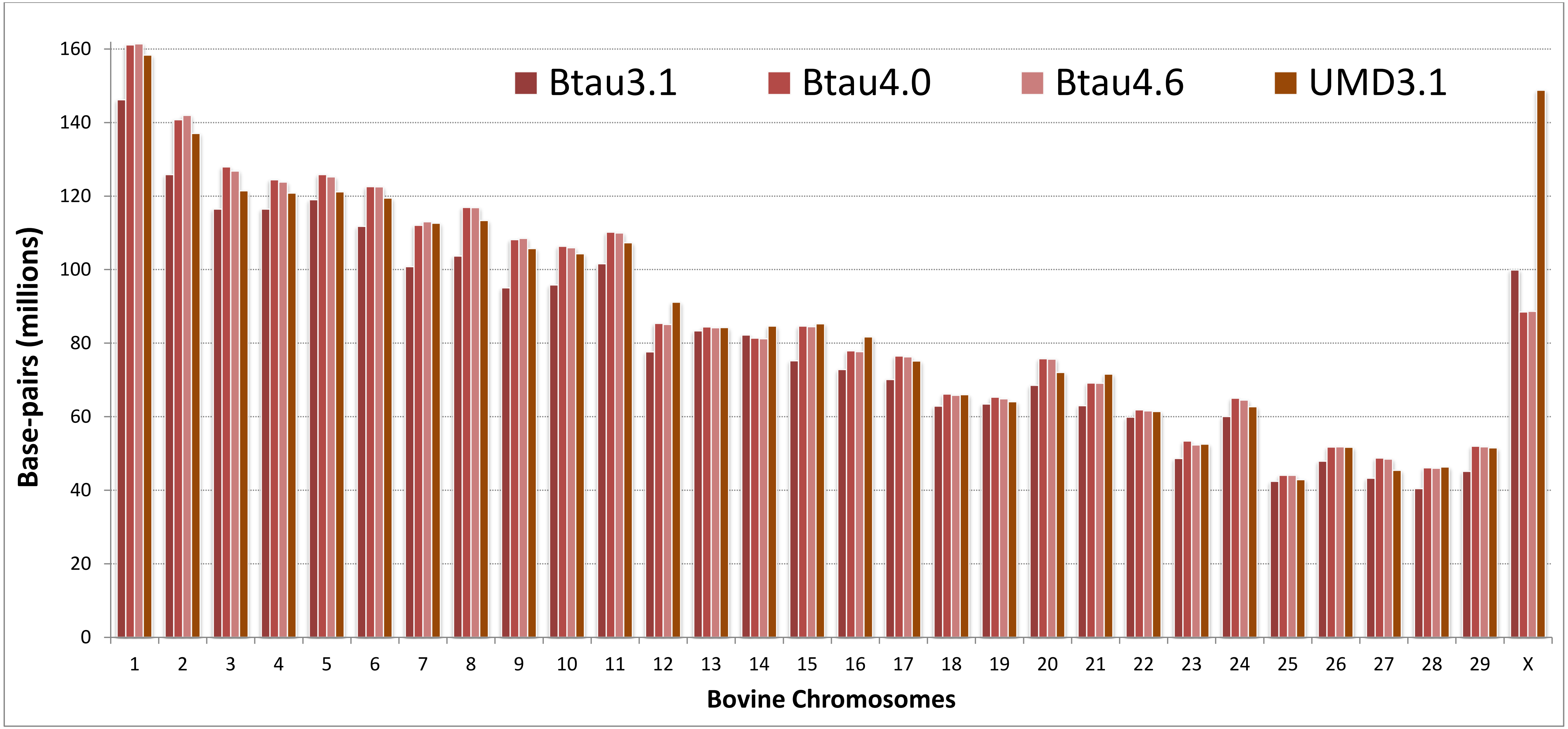

S2 Fig. Chromosome-wise length (million base-pairs or Mbp) of bovine genome assemblies (Btau3.1, Btau4.0, Btau4.6 and UMD3.1)
