# Supplementary material for: A Meta-Assembly of Selection Signatures in Cattle
Source: PLoS One. 2016 Apr 5;11(4):e0153013. doi: 10.1371/journal.pone.0153013 (PMC4821596; doi:10.1371/journal.pone.0153013)
Supplement: S3 Fig — Chromosome-wise (x-axis) SNP density (y-axis) of various genotyping Bovine SNPchip assays; A: 10K, B: BHMC (mapped on Btau 3.1 and Btau 4.0), C: 50K (from two version, v1 and v2) and D: Illumina’s 800K and Affymetrix AFFXB1P (~700K but features for ~2.5 million SNPs). (PDF) [file pone.0153013.s010.pdf]

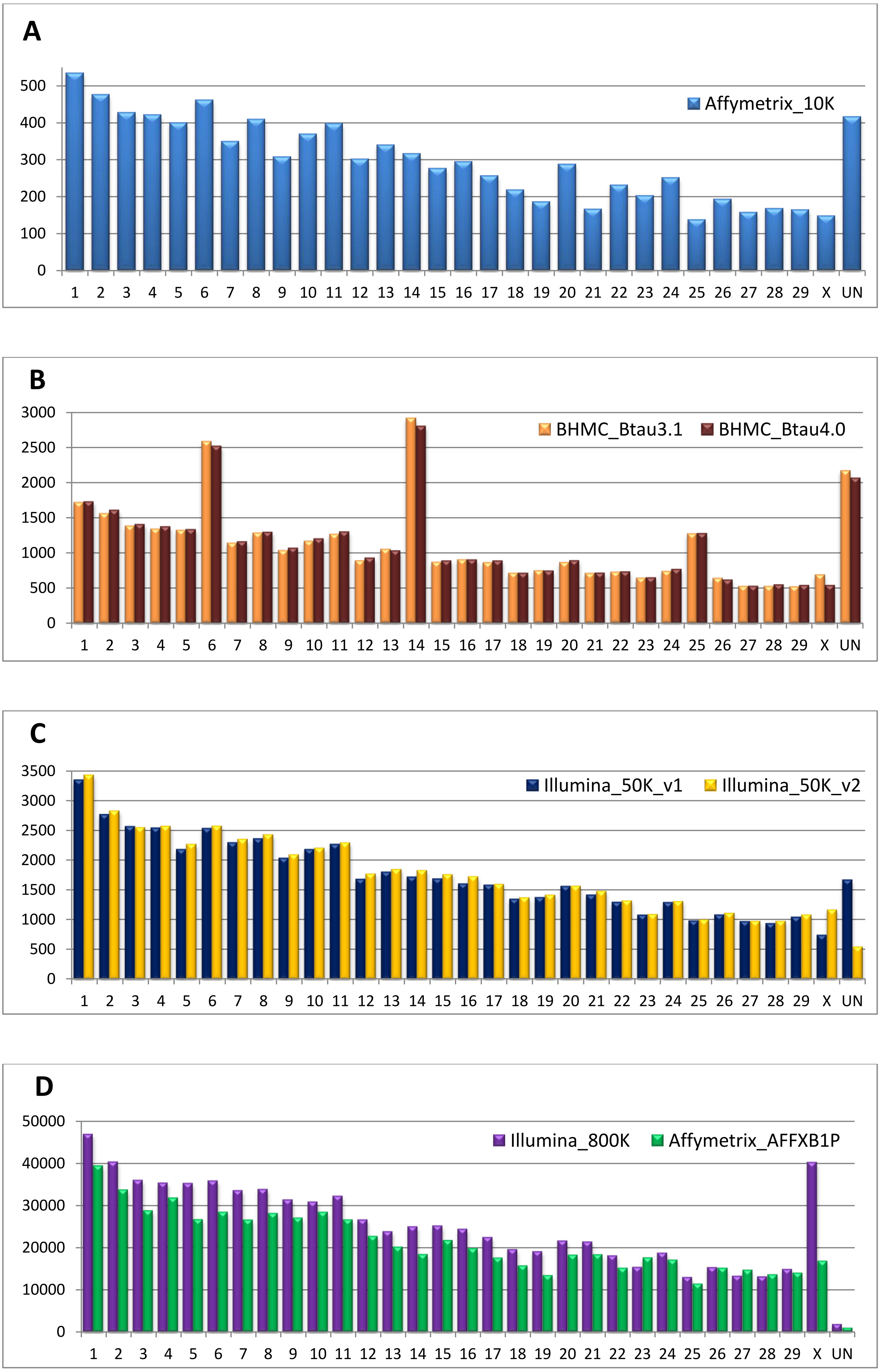

S3 Fig. Chromosome-wise (x-axis) SNP density (y-axis) of various genotyping Bovine SNPchip assays; **A**: 10K, **B**: BHMC (mapped on Btau 3.1 and Btau 4.0), **C**: 50K (from two version, v1 and v2) and **D**: Illumina's 800K and Affymetrix AFFXB1P (~700K but features for ~2.5 million SNPs).
