# Supplementary figures and images for: A Meta-Assembly of Selection Signatures in Cattle
Source: PLoS One. 2016 Apr 5;11(4):e0153013. doi: 10.1371/journal.pone.0153013 (PMC4821596; doi:10.1371/journal.pone.0153013)

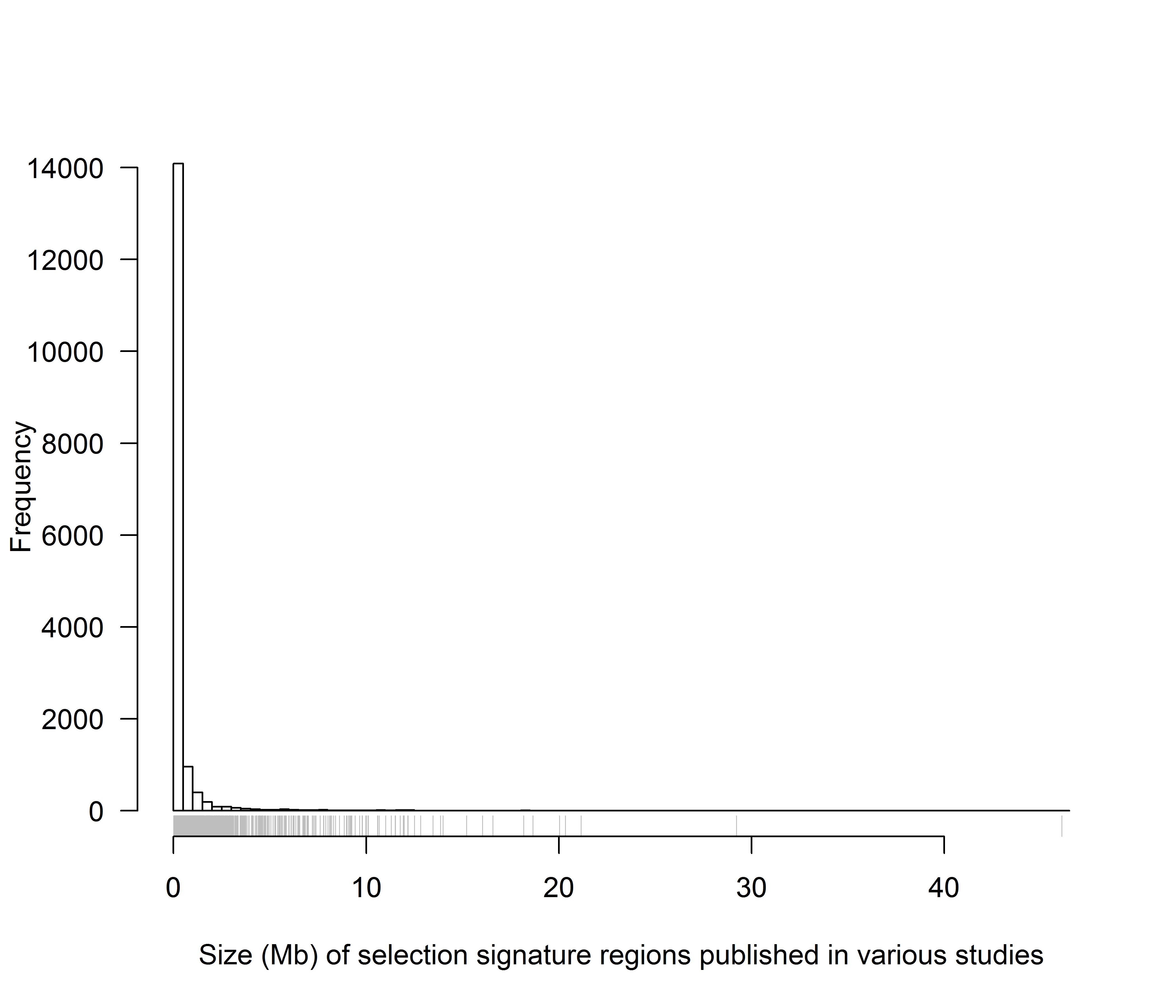

Supplement: S4 Fig — (TIFF) [file pone.0153013.s011.tiff]

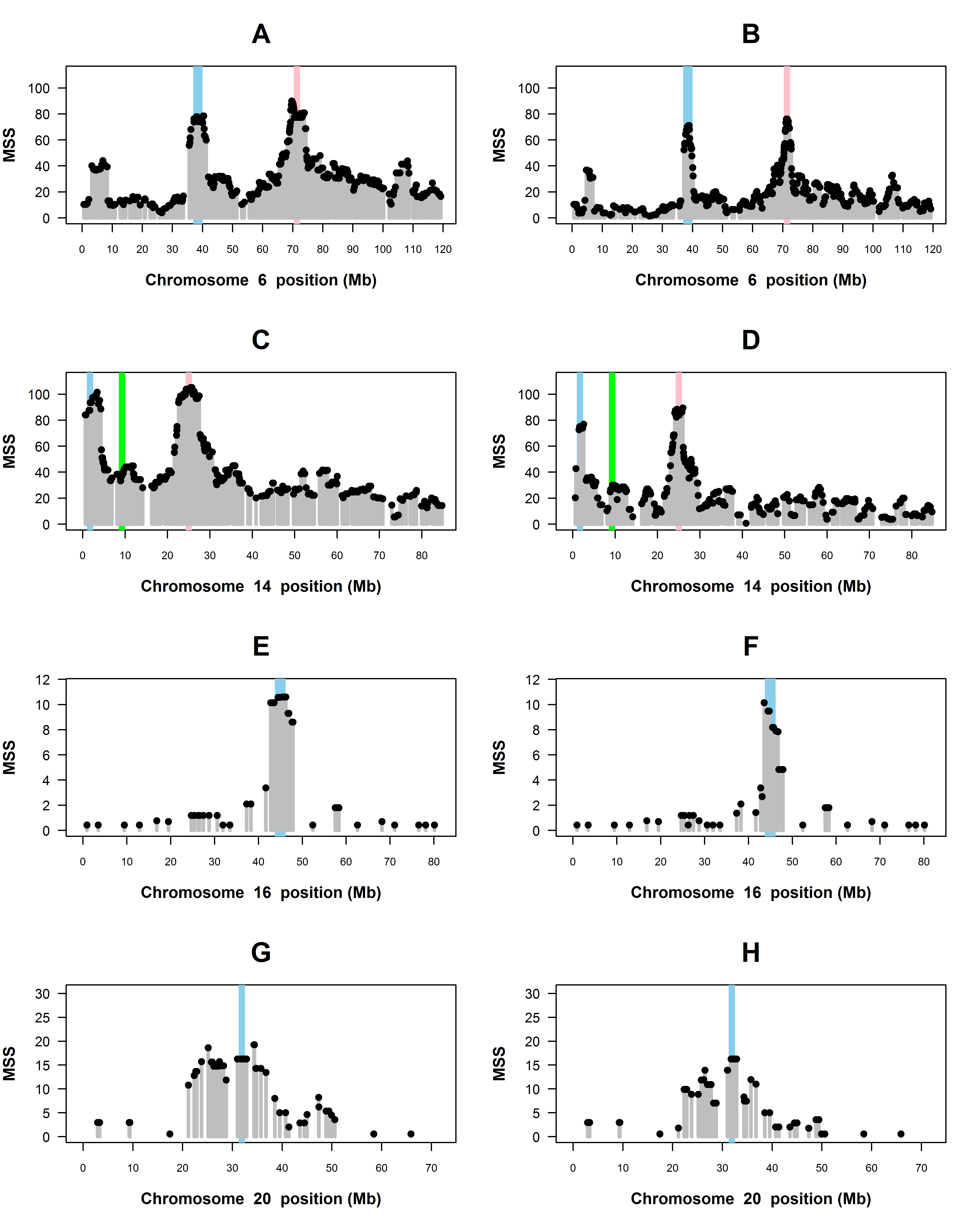

Supplement: S5 Fig — The left (A, C, E, G) and right (B, D, F, H) panels show chromosome-wise MSS using 5 Mb and 2 Mb sliding window spans, respectively. A-B: Chromosome 6 of European group highlighted at ABCG2, LAP3, NCAPG and LCORL gene located between 37.95–39.00 Mbp (blue bar) and at PDGFRA and KIT genes located between 71.37–71.42 Mbp (pink bar). C-D: Chromosome 14 of European group highlighted at DGAT1 (1.69–1.96 Mbp; blue bar), TG (9.26–9.51 Mbp; green bar) and PLAG1-CHCHD7 (25.00–25.06 Mbp; pink bar) region. E-F: Chromosome 16 of Angus highlighting span (44.45–45.88 Mbp) between NMNAT1 to RERE genes (blue bar). G-H: Chromosome 20 of Holstein highlighting GHR region at 31.89–32.07 Mb (blue bar). (PNG) [file pone.0153013.s012.png]

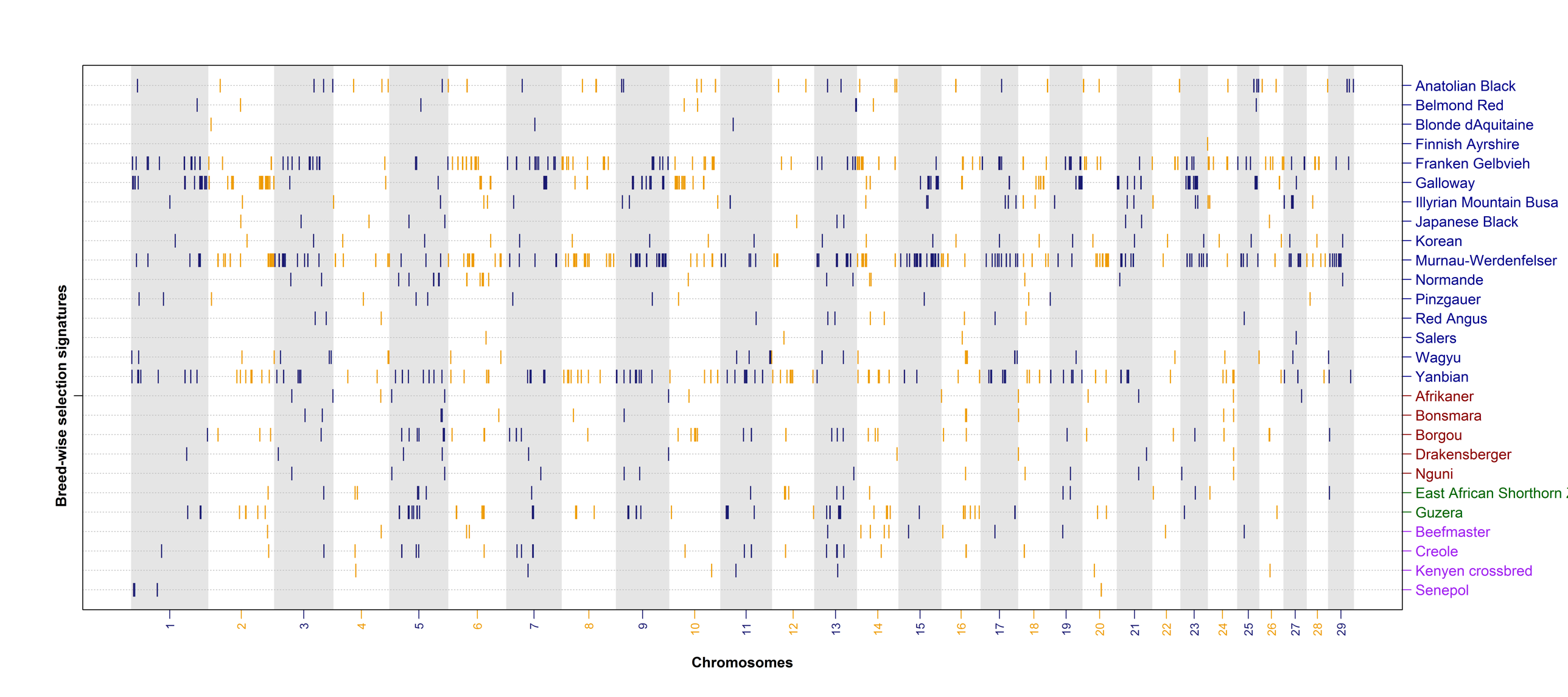

Supplement: S6 Fig — Hence, Meta-score cannot be computed for these breeds, however, these results have been used accordingly to the particular breed-type in various group-wise Meta-assemblies of European, Zebu, African and Composite breeds. (TIFF) [file pone.0153013.s013.tiff]
