# Supplementary material for: A Meta-Assembly of Selection Signatures in Cattle
Source: PLoS One. 2016 Apr 5;11(4):e0153013. doi: 10.1371/journal.pone.0153013 (PMC4821596; doi:10.1371/journal.pone.0153013)
Supplement: S7 Fig — Top panel shows Meta-selection-scores (MSS) computed for the group of all European breeds and provides a comparison of MSS and gene density (genes per Mb) distribution on each chromosome. Lower panel shows location of published selection signature regions. Symbol and colour of each dot point represent selection test (symbols in the bottom legend) and candidate breed (European breed coloured legend on the left), respectively. In lower panel, the central or top (if given) score region is shown with a dot point and spans of extended regions are shown with a horizontal solid-line. Labels on right-hand side of lower panel shows the SNP genotyping panel used in the particular study and the red, orange and pink colours of each SNP panel represent study-wise threshold of top 0.1%, 1% and 5%, respectively. (PDF) [file pone.0153013.s014.pdf]

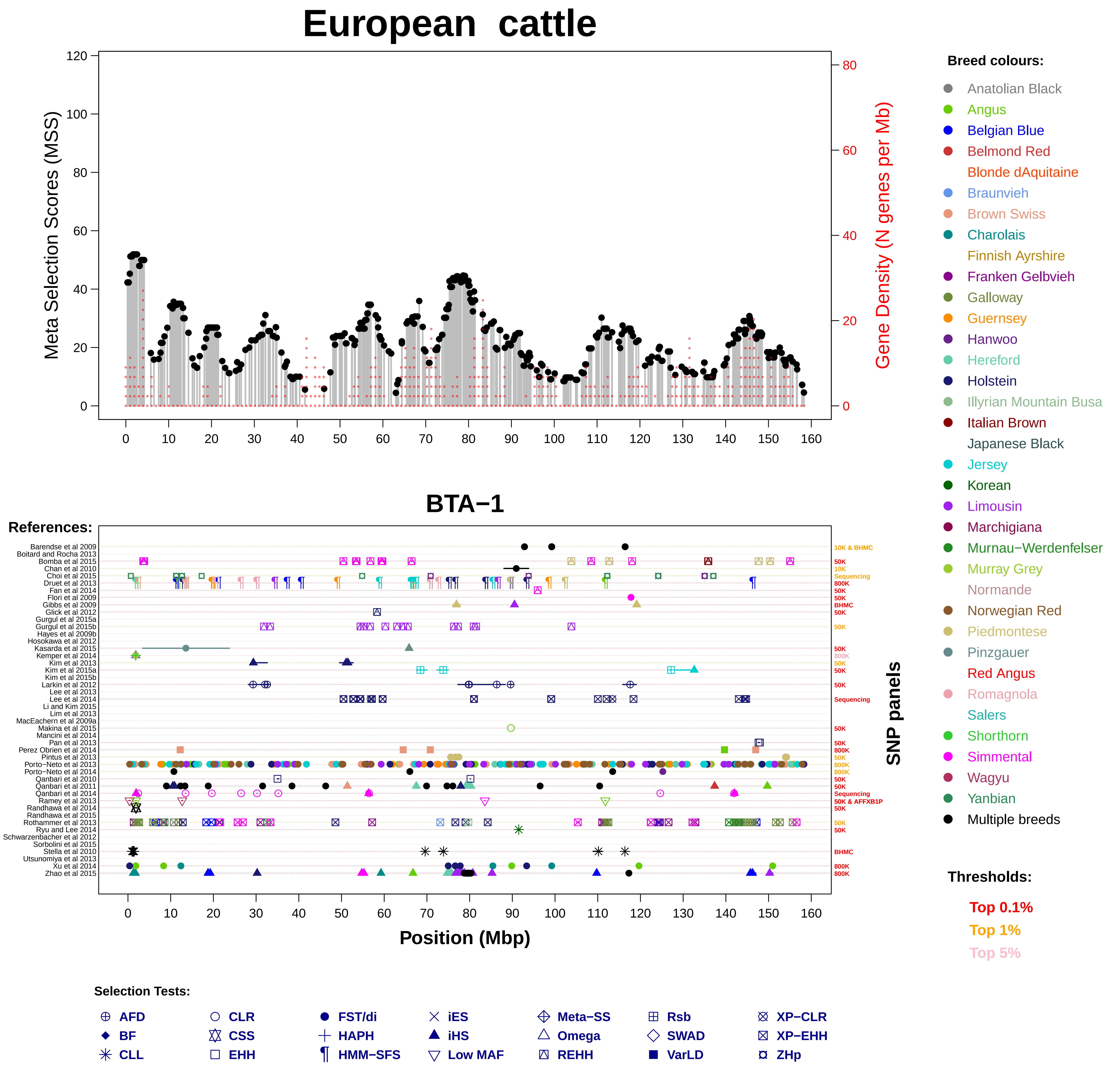
European cattle
Meta Selection Scores (MSS)
Gene Density (N genes per Mb)
BTA−1
References:
Barendse et al 2009
Boitard and Rocha 2013
Bomba et al 2015
Chan et al 2010
Choi et al 2015
Druet et al 2013
Fan et al 2014
Flori et al 2009
Gibbs et al 2009
Glick et al 2012
Gurgul et al 2015a
Gurgul et al 2015b
Hayes et al 2009b
Hosokawa et al 2012
Kasarda et al 2015
Kemper et al 2014
Kim et al 2013
Kim et al 2015a
Kim et al 2015b
Larkin et al 2012
Lee et al 2013
Lee et al 2014
Li and Kim 2015
Lim et al 2013
MacEachern et al 2009a
Makina et al 2015
Mancini et al 2014
Pan et al 2013
Perez Obrien et al 2014
Pintus et al 2013
Porto−Neto et al 2013
Porto−Neto et al 2014
Qanbari et al 2010
Qanbari et al 2011
Qanbari et al 2014
Ramey et al 2013
Randhawa et al 2014
Randhawa et al 2015
Rothammer et al 2013
Ryu and Lee 2014
Schwarzenbacher et al 2012
Sorbolini et al 2015
Stella et al 2010
Utsunomiya et al 2013
Xu et al 2014
Zhao et al 2015
SNP panels
10K & BHMC
50K
10K
Sequencing
800K
BHMC
800K
Sequencing
50K & AFFXB1P
Position (Mbp)
Breed colours:
Anatolian Black
Angus
Belgian Blue
Belmond Red
Blonde dAquitaine
Braunvieh
Brown Swiss
Charolais
Finnish Ayrshire
Franken Gelbvieh
Galloway
Guernsey
Hanwoo
Hereford
Holstein
Illyrian Mountain Busa
Italian Brown
Japanese Black
Jersey
Korean
Limousin
Marchigiana
Murnau−Werdenfelser
Murray Grey
Normande
Norwegian Red
Piedmontese
Pinzgauer
Red Angus
Romagnola
Salers
Shorthorn
Simmental
Wagyu
Yanbian
Multiple breeds
Thresholds:
Top 0.1%
Top 1%
Top 5%
Selection Tests:
AFD
BF
CLL
CLR
CSS
EHH
FST/di
HAPH
HMM−SFS
iES
iHS
Low MAF
Meta−SS
Omega
REHH
Rsb
SWAD
VarLD
XP−CLR
XP−EHH
ZHp

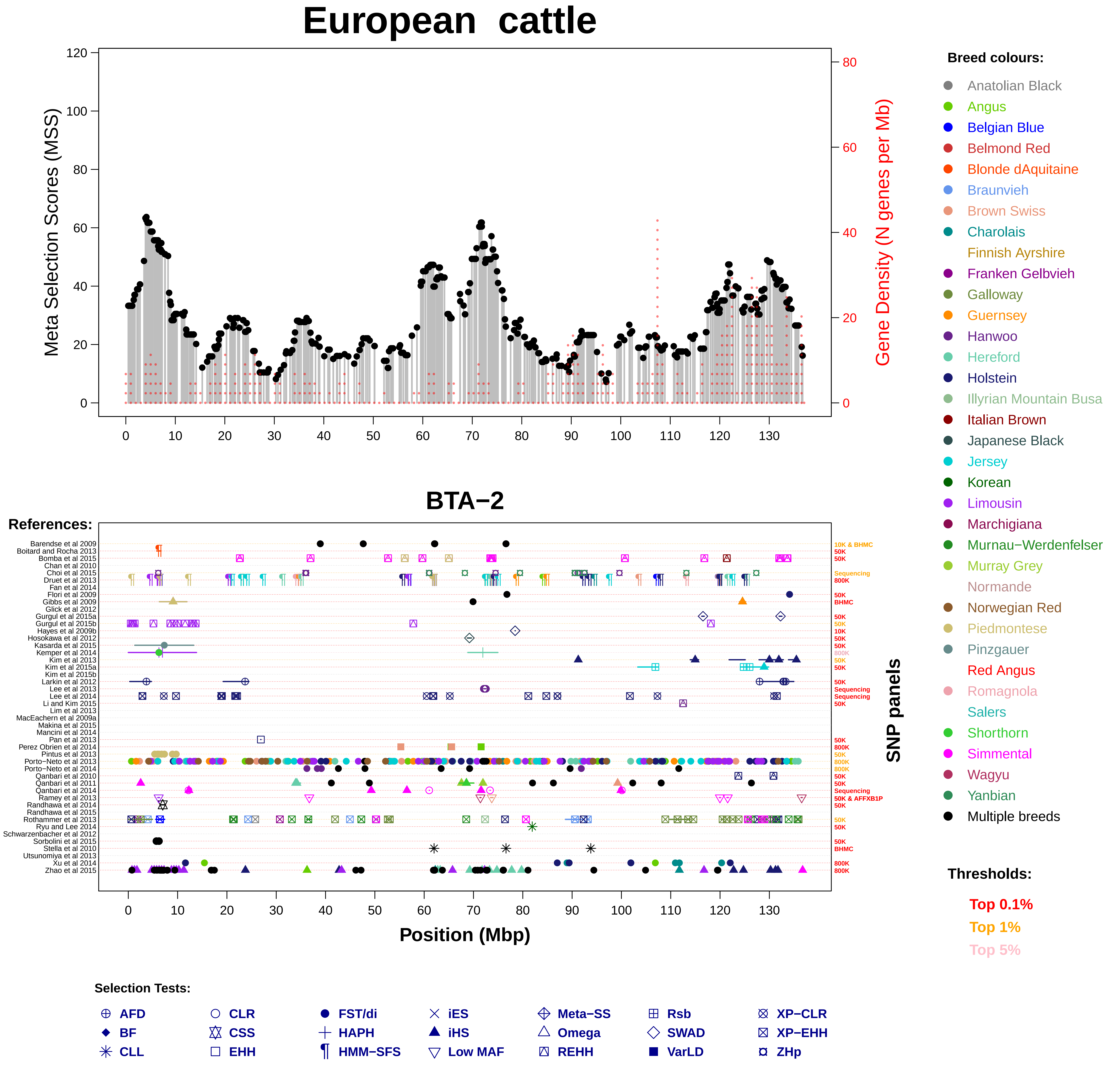
European cattle
Meta Selection Scores (MSS)
Gene Density (N genes per Mb)
BTA−2
References:
Barendse et al 2009
Boitard and Rocha 2013
Bomba et al 2015
Chan et al 2010
Choi et al 2015
Druet et al 2013
Fan et al 2014
Flori et al 2009
Gibbs et al 2009
Glick et al 2012
Gurgul et al 2015a
Gurgul et al 2015b
Hayes et al 2009b
Hosokawa et al 2012
Kasarda et al 2015
Kemper et al 2014
Kim et al 2013
Kim et al 2015a
Kim et al 2015b
Larkin et al 2012
Lee et al 2013
Lee et al 2014
Li and Kim 2015
Lim et al 2013
MacEachern et al 2009a
Makina et al 2015
Mancini et al 2014
Pan et al 2013
Perez Obrien et al 2014
Pintus et al 2013
Porto−Neto et al 2013
Porto−Neto et al 2014
Qanbari et al 2010
Qanbari et al 2011
Qanbari et al 2014
Ramey et al 2013
Randhawa et al 2014
Randhawa et al 2015
Rothammer et al 2013
Ryu and Lee 2014
Schwarzenbacher et al 2012
Sorbolini et al 2015
Stella et al 2010
Utsunomiya et al 2013
Xu et al 2014
Zhao et al 2015
SNP panels
Position (Mbp)
Breed colours:
Anatolian Black
Angus
Belgian Blue
Belmond Red
Blonde dAquitaine
Braunvieh
Brown Swiss
Charolais
Finnish Ayrshire
Franken Gelbvieh
Galloway
Guernsey
Hanwoo
Hereford
Holstein
Illyrian Mountain Busa
Italian Brown
Japanese Black
Jersey
Korean
Limousin
Marchigiana
Murnau−Werdenfelser
Murray Grey
Normande
Norwegian Red
Piedmontese
Pinzgauer
Red Angus
Romagnola
Salers
Shorthorn
Simmental
Wagyu
Yanbian
Multiple breeds
Thresholds:
Top 0.1%
Top 1%
Top 5%
Selection Tests:
AFD
BF
CLL
CLR
CSS
EHH
FST/di
HAPH
HMM−SFS
iES
iHS
Low MAF
Meta−SS
Omega
REHH
Rsb
SWAD
VarLD
XP−CLR
XP−EHH
ZHp

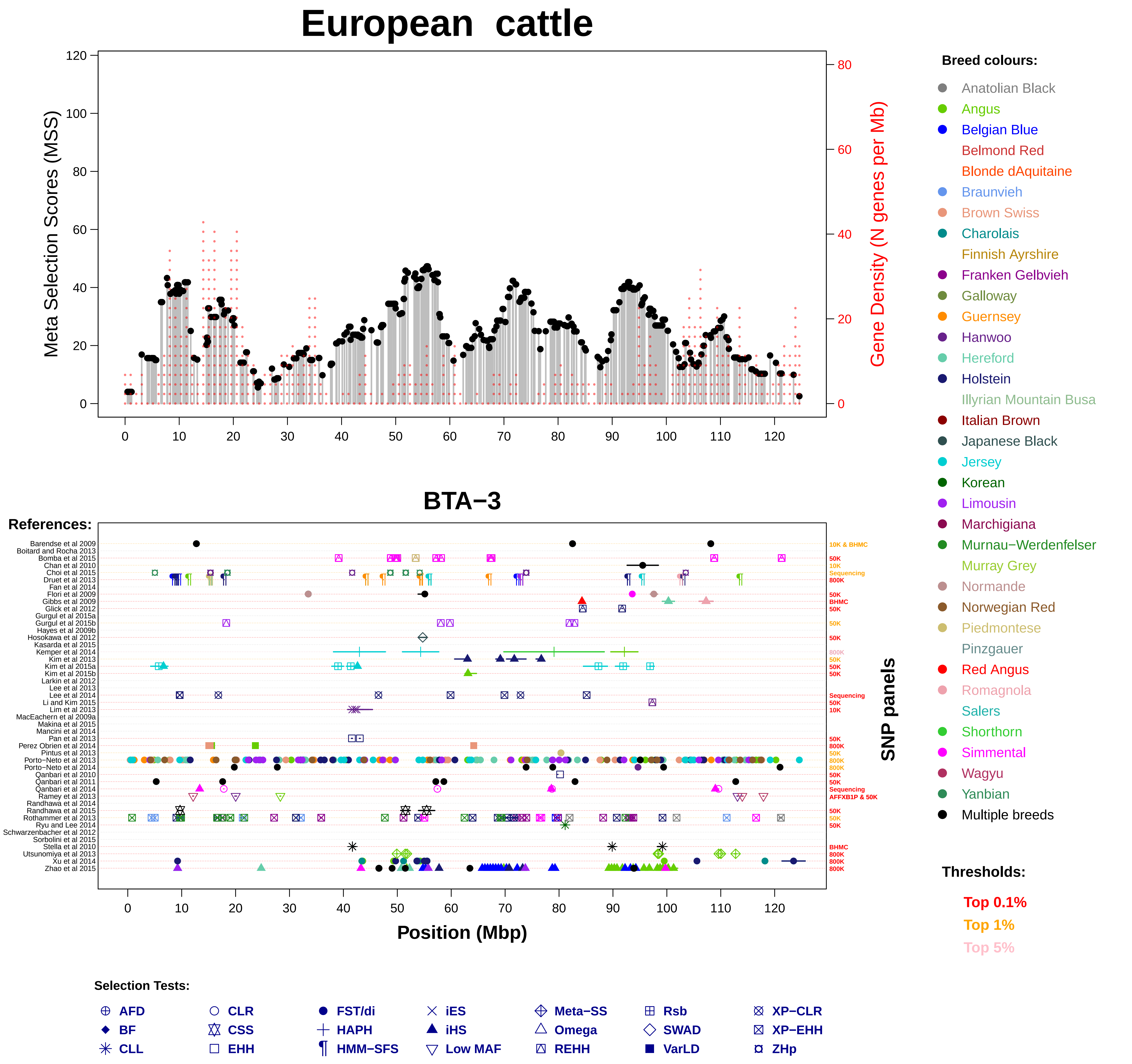
European cattle
Meta Selection Scores (MSS)
Gene Density (N genes per Mb)
BTA−3
References:
Barendse et al 2009
Boitard and Rocha 2013
Bomba et al 2015
Chan et al 2010
Choi et al 2015
Druet et al 2013
Fan et al 2014
Flori et al 2009
Gibbs et al 2009
Glick et al 2012
Gurgul et al 2015a
Gurgul et al 2015b
Hayes et al 2009b
Hosokawa et al 2012
Kasarda et al 2015
Kemper et al 2014
Kim et al 2013
Kim et al 2015a
Kim et al 2015b
Larkin et al 2012
Lee et al 2013
Lee et al 2014
Li and Kim 2015
Lim et al 2013
MacEachern et al 2009a
Makina et al 2015
Mancini et al 2014
Pan et al 2013
Perez Obrien et al 2014
Pintus et al 2013
Porto−Neto et al 2013
Porto−Neto et al 2014
Qanbari et al 2010
Qanbari et al 2011
Qanbari et al 2014
Ramey et al 2013
Randhawa et al 2014
Randhawa et al 2015
Rothammer et al 2013
Ryu and Lee 2014
Schwarzenbacher et al 2012
Sorbolini et al 2015
Stella et al 2010
Utsunomiya et al 2013
Xu et al 2014
Zhao et al 2015
10K & BHMC
50K
10K
Sequencing
800K
50K
BHMC
50K
50K
50K
800K
50K
50K
50K
Sequencing
50K
10K
50K
800K
50K
800K
800K
50K
50K
Sequencing
AFFXB1P & 50K
50K
50K
50K
BHMC
800K
800K
800K
SNP panels
Position (Mbp)
Breed colours:
Anatolian Black
Angus
Belgian Blue
Belmond Red
Blonde dAquitaine
Braunvieh
Brown Swiss
Charolais
Finnish Ayrshire
Franken Gelbvieh
Galloway
Guernsey
Hanwoo
Hereford
Holstein
Illyrian Mountain Busa
Italian Brown
Japanese Black
Jersey
Korean
Limousin
Marchigiana
Murnau−Werdenfelser
Murray Grey
Normande
Norwegian Red
Piedmontese
Pinzgauer
Red Angus
Romagnola
Salers
Shorthorn
Simmental
Wagyu
Yanbian
Multiple breeds
Thresholds:
Top 0.1%
Top 1%
Top 5%
Selection Tests:
AFD
BF
CLL
CLR
CSS
EHH
FST/di
HAPH
HMM−SFS
iES
iHS
Low MAF
Meta−SS
Omega
REHH
Rsb
SWAD
VarLD
XP−CLR
XP−EHH
ZHp

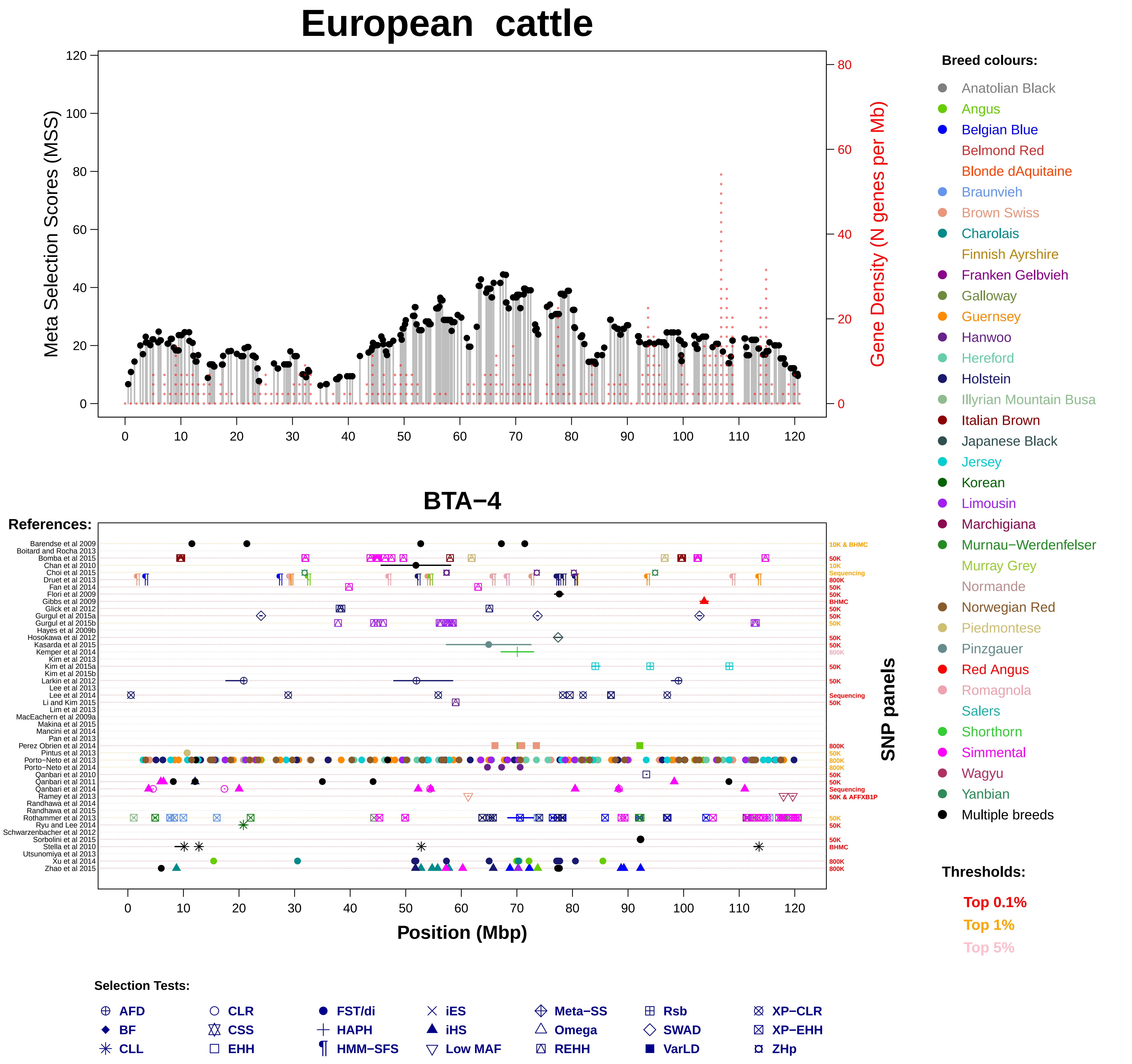

European cattle
Meta Selection Scores (MSS)
Gene Density (N genes per Mb)
BTA−4
References:
Barendse et al 2009
Boitard and Rocha 2013
Bomba et al 2015
Chan et al 2010
Choi et al 2015
Druet et al 2013
Fan et al 2014
Flori et al 2009
Gibbs et al 2009
Glick et al 2012
Gurgul et al 2015a
Gurgul et al 2015b
Hayes et al 2009b
Hosokawa et al 2012
Kasarda et al 2015
Kemper et al 2014
Kim et al 2013
Kim et al 2015a
Kim et al 2015b
Larkin et al 2012
Lee et al 2013
Lee et al 2014
Li and Kim 2015
Lim et al 2013
MacEachern et al 2009a
Makina et al 2015
Mancini et al 2014
Pan et al 2013
Perez Obrien et al 2014
Pintus et al 2013
Porto−Neto et al 2013
Porto−Neto et al 2014
Qanbari et al 2010
Qanbari et al 2011
Qanbari et al 2014
Ramey et al 2013
Randhawa et al 2014
Randhawa et al 2015
Rothammer et al 2013
Ryu and Lee 2014
Schwarzenbacher et al 2012
Sorbolini et al 2015
Stella et al 2010
Utsunomiya et al 2013
Xu et al 2014
Zhao et al 2015
10K & BHMC
50K
10K
Sequencing
800K
50K
50K
BHMC
50K
50K
50K
50K
50K
800K
50K
50K
Sequencing
50K
800K
50K
800K
50K
50K
Sequencing
50K & AFFXB1P
50K
50K
50K
BHMC
800K
800K
SNP panels
Position (Mbp)
Breed colours:
Anatolian Black
Angus
Belgian Blue
Belmond Red
Blonde dAquitaine
Braunvieh
Brown Swiss
Charolais
Finnish Ayrshire
Franken Gelbvieh
Galloway
Guernsey
Hanwoo
Hereford
Holstein
Illyrian Mountain Busa
Italian Brown
Japanese Black
Jersey
Korean
Limousin
Marchigiana
Murnau−Werdenfelser
Murray Grey
Normande
Norwegian Red
Piedmontese
Pinzgauer
Red Angus
Romagnola
Salers
Shorthorn
Simmental
Wagyu
Yanbian
Multiple breeds
Thresholds:
Top 0.1%
Top 1%
Top 5%
Selection Tests:
AFD
BF
CLL
CLR
CSS
EHH
FST/di
HAPH
HMM−SFS
iES
iHS
Low MAF
Meta−SS
Omega
REHH
Rsb
SWAD
VarLD
XP−CLR
XP−EHH
ZHp

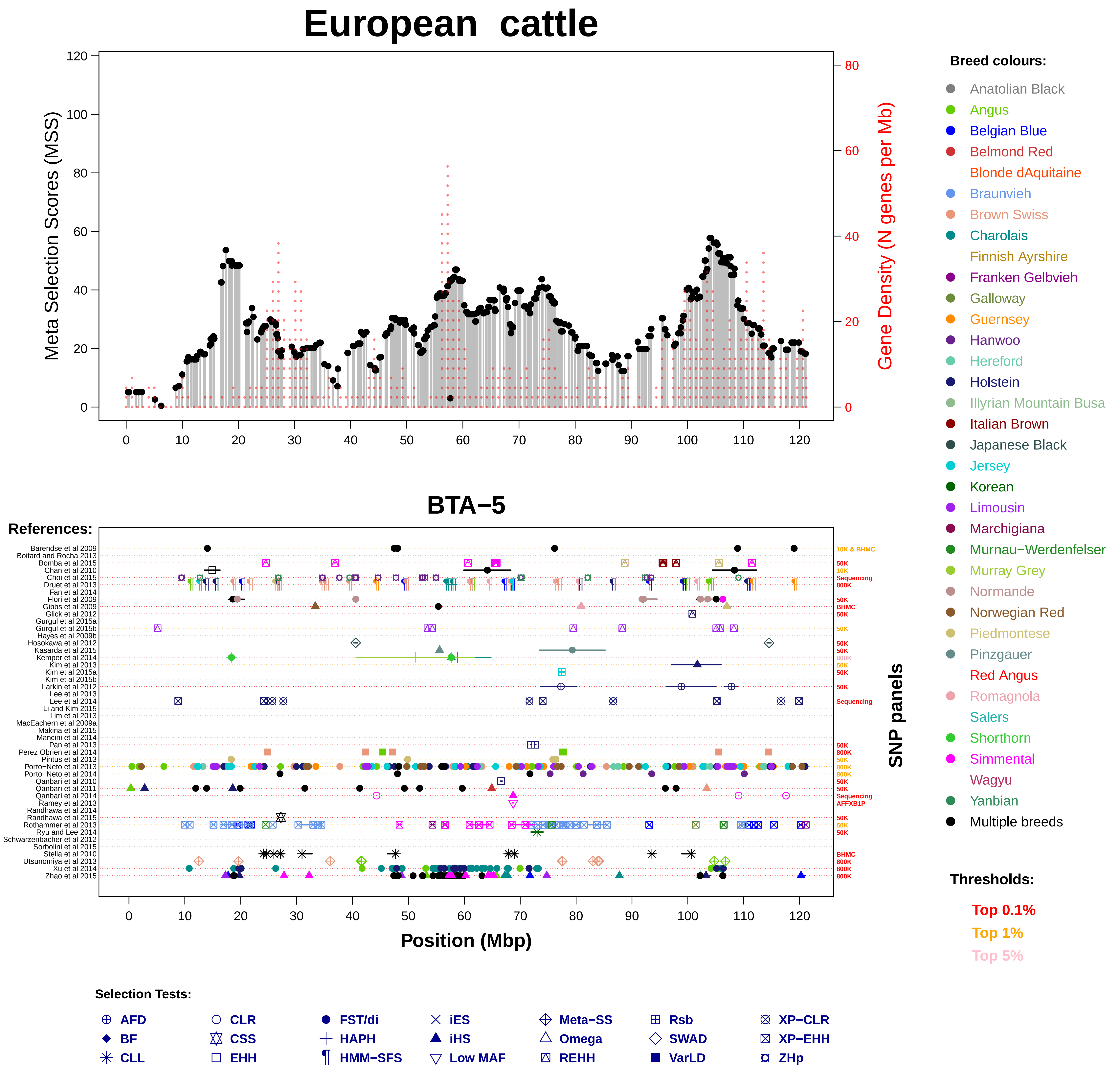

European cattle
Meta Selection Scores (MSS)
Gene Density (N genes per Mb)
BTA−5
References:
Barendse et al 2009
Boitard and Rocha 2013
Bomba et al 2015
Chan et al 2010
Choi et al 2015
Druet et al 2013
Fan et al 2014
Flori et al 2009
Gibbs et al 2009
Glick et al 2012
Gurgul et al 2015a
Gurgul et al 2015b
Hayes et al 2009b
Hosokawa et al 2012
Kasarda et al 2015
Kemper et al 2014
Kim et al 2013
Kim et al 2015a
Kim et al 2015b
Larkin et al 2012
Lee et al 2013
Lee et al 2014
Li and Kim 2015
Lim et al 2013
MacEachern et al 2009a
Makina et al 2015
Mancini et al 2014
Pan et al 2013
Perez Obrien et al 2014
Pintus et al 2013
Porto−Neto et al 2013
Porto−Neto et al 2014
Qanbari et al 2010
Qanbari et al 2011
Qanbari et al 2014
Ramey et al 2013
Randhawa et al 2014
Randhawa et al 2015
Rothammer et al 2013
Ryu and Lee 2014
Schwarzenbacher et al 2012
Sorbolini et al 2015
Stella et al 2010
Utsunomiya et al 2013
Xu et al 2014
Zhao et al 2015
10K & BHMC
50K
10K
Sequencing
800K
50K
BHMC
50K
50K
50K
50K
800K
50K
50K
50K
Sequencing
50K
800K
50K
800K
800K
50K
50K
Sequencing
AFFXB1P
50K
50K
50K
BHMC
800K
800K
800K
SNP panels
Position (Mbp)
Breed colours:
Anatolian Black
Angus
Belgian Blue
Belmond Red
Blonde dAquitaine
Braunvieh
Brown Swiss
Charolais
Finnish Ayrshire
Franken Gelbvieh
Galloway
Guernsey
Hanwoo
Hereford
Holstein
Illyrian Mountain Busa
Italian Brown
Japanese Black
Jersey
Korean
Limousin
Marchigiana
Murnau−Werdenfelser
Murray Grey
Normande
Norwegian Red
Piedmontese
Pinzgauer
Red Angus
Romagnola
Salers
Shorthorn
Simmental
Wagyu
Yanbian
Multiple breeds
Thresholds:
Top 0.1%
Top 1%
Top 5%
Selection Tests:
AFD
BF
CLL
CLR
CSS
EHH
FST/di
HAPH
HMM−SFS
iES
iHS
Low MAF
Meta−SS
Omega
REHH
Rsb
SWAD
VarLD
XP−CLR
XP−EHH
ZHp

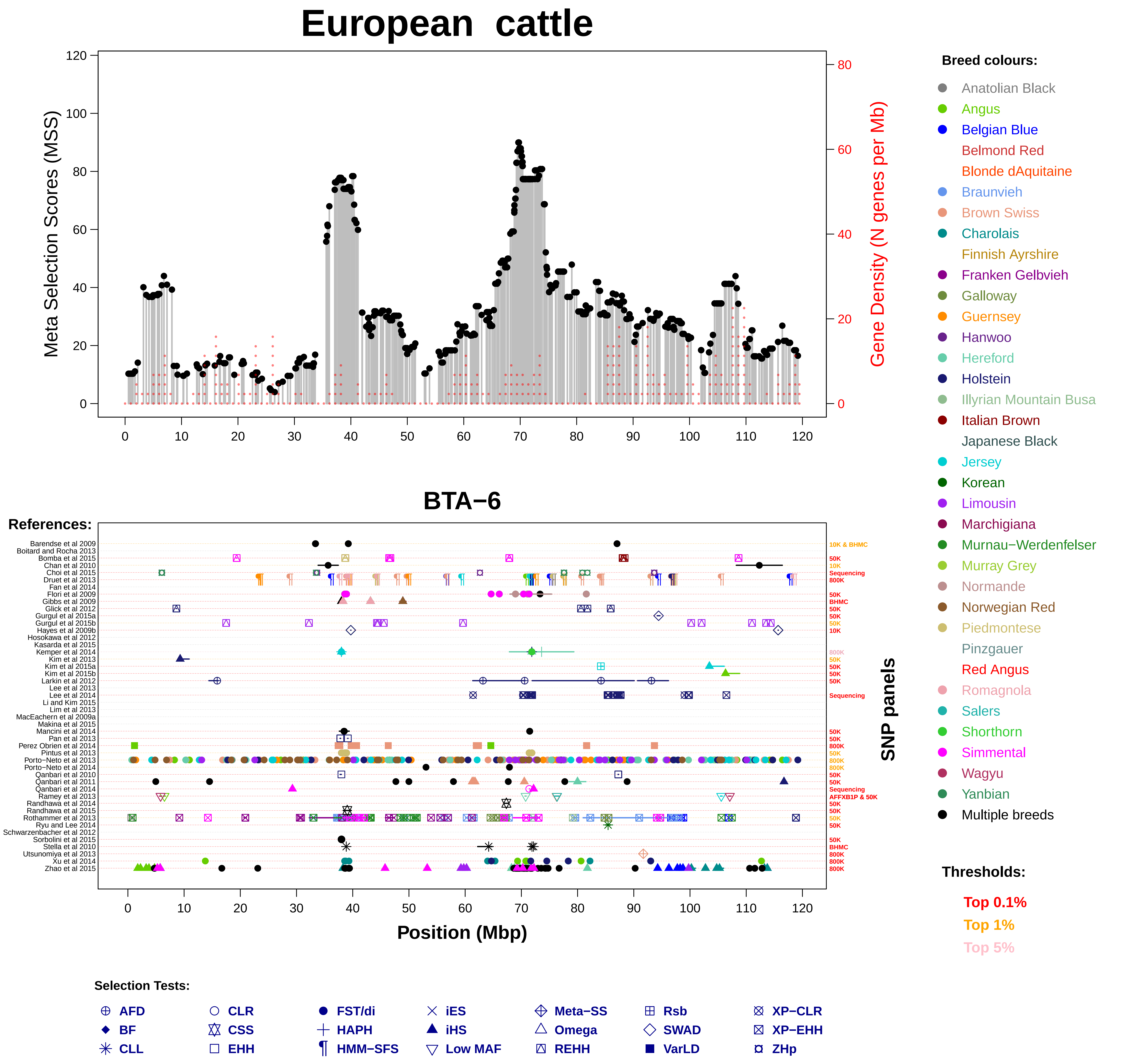

European cattle
Meta Selection Scores (MSS)
Gene Density (N genes per Mb)
BTA−6
References:
Barendse et al 2009
Boitard and Rocha 2013
Bomba et al 2015
Chan et al 2010
Choi et al 2015
Druet et al 2013
Fan et al 2014
Flori et al 2009
Gibbs et al 2009
Glick et al 2012
Gurgul et al 2015a
Gurgul et al 2015b
Hayes et al 2009b
Hosokawa et al 2012
Kasarda et al 2015
Kemper et al 2014
Kim et al 2013
Kim et al 2015a
Kim et al 2015b
Larkin et al 2012
Lee et al 2013
Lee et al 2014
Li and Kim 2015
Lim et al 2013
MacEachern et al 2009a
Makina et al 2015
Mancini et al 2014
Pan et al 2013
Perez Obrien et al 2014
Pintus et al 2013
Porto−Neto et al 2013
Porto−Neto et al 2014
Qanbari et al 2010
Qanbari et al 2011
Qanbari et al 2014
Ramey et al 2013
Randhawa et al 2014
Randhawa et al 2015
Rothammer et al 2013
Ryu and Lee 2014
Schwarzenbacher et al 2012
Sorbolini et al 2015
Stella et al 2010
Utsunomiya et al 2013
Xu et al 2014
Zhao et al 2015
SNP panels
Position (Mbp)
Breed colours:
Anatolian Black
Angus
Belgian Blue
Belmond Red
Blonde dAquitaine
Braunvieh
Brown Swiss
Charolais
Finnish Ayrshire
Franken Gelbvieh
Galloway
Guernsey
Hanwoo
Hereford
Holstein
Illyrian Mountain Busa
Italian Brown
Japanese Black
Jersey
Korean
Limousin
Marchigiana
Murnau−Werdenfelser
Murray Grey
Normande
Norwegian Red
Piedmontese
Pinzgauer
Red Angus
Romagnola
Salers
Shorthorn
Simmental
Wagyu
Yanbian
Multiple breeds
Thresholds:
Top 0.1%
Top 1%
Top 5%
Selection Tests:
AFD
BF
CLL
CLR
CSS
EHH
FST/di
HAPH
HMM−SFS
iES
iHS
Low MAF
Meta−SS
Omega
REHH
Rsb
SWAD
VarLD
XP−CLR
XP−EHH
ZHp

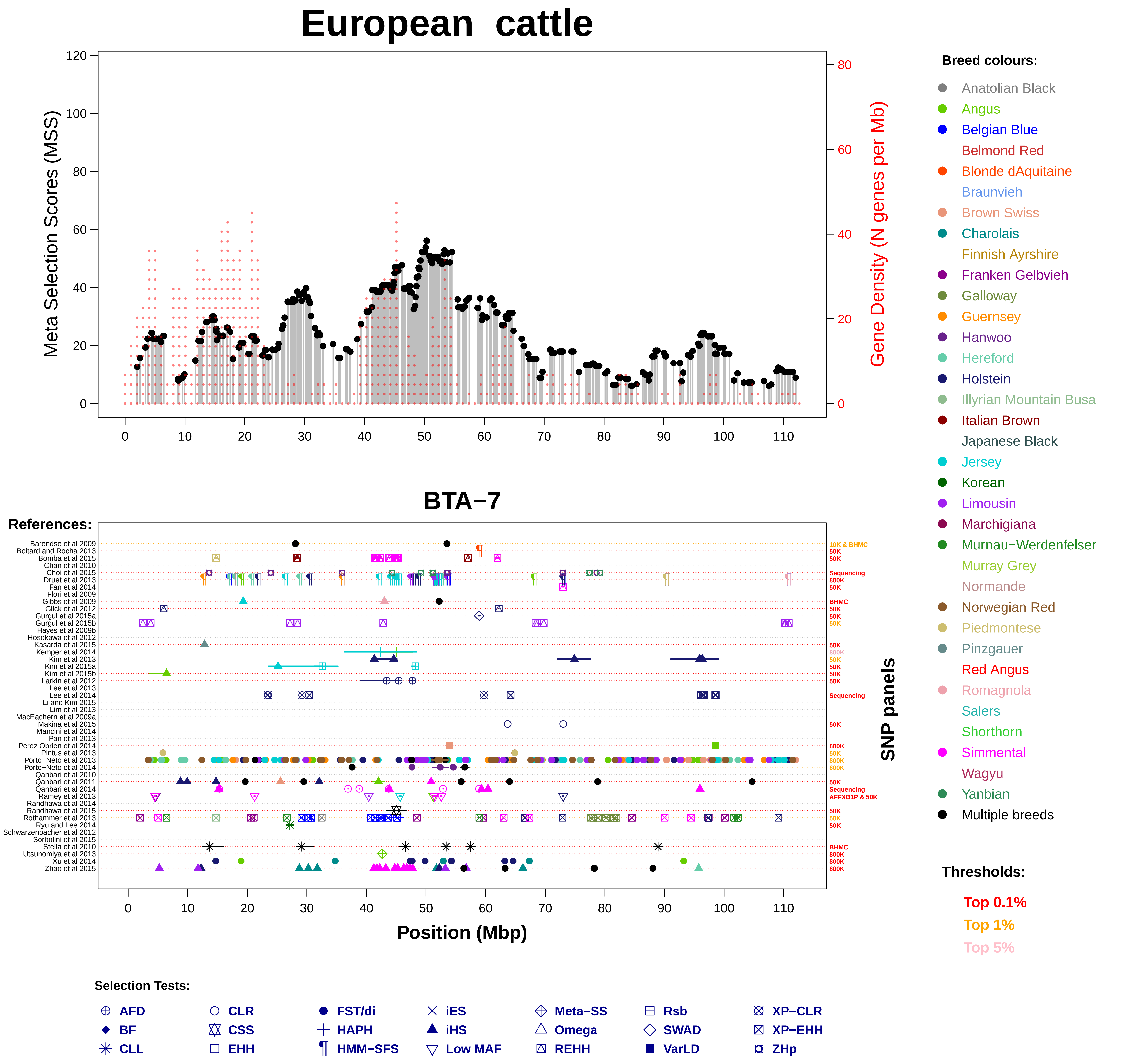
European cattle
Meta Selection Scores (MSS)
Gene Density (N genes per Mb)
BTA−7
References:
Barendse et al 2009
Boitard and Rocha 2013
Bomba et al 2015
Chan et al 2010
Choi et al 2015
Druet et al 2013
Fan et al 2014
Flori et al 2009
Gibbs et al 2009
Glick et al 2012
Gurgul et al 2015a
Gurgul et al 2015b
Hayes et al 2009b
Hosokawa et al 2012
Kasarda et al 2015
Kemper et al 2014
Kim et al 2013
Kim et al 2015a
Kim et al 2015b
Larkin et al 2012
Lee et al 2013
Lee et al 2014
Li and Kim 2015
Lim et al 2013
MacEachern et al 2009a
Makina et al 2015
Mancini et al 2014
Pan et al 2013
Perez Obrien et al 2014
Pintus et al 2013
Porto−Neto et al 2013
Porto−Neto et al 2014
Qanbari et al 2010
Qanbari et al 2011
Qanbari et al 2014
Ramey et al 2013
Randhawa et al 2014
Randhawa et al 2015
Rothammer et al 2013
Ryu and Lee 2014
Schwarzenbacher et al 2012
Sorbolini et al 2015
Stella et al 2010
Utsunomiya et al 2013
Xu et al 2014
Zhao et al 2015
SNP panels
10K & BHMC
50K
Sequencing
800K
BHMC
AFFXB1P & 50K
Position (Mbp)
Breed colours:
Anatolian Black
Angus
Belgian Blue
Belmond Red
Blonde dAquitaine
Braunvieh
Brown Swiss
Charolais
Finnish Ayrshire
Franken Gelbvieh
Galloway
Guernsey
Hanwoo
Hereford
Holstein
Illyrian Mountain Busa
Italian Brown
Japanese Black
Jersey
Korean
Limousin
Marchigiana
Murnau−Werdenfelser
Murray Grey
Normande
Norwegian Red
Piedmontese
Pinzgauer
Red Angus
Romagnola
Salers
Shorthorn
Simmental
Wagyu
Yanbian
Multiple breeds
Thresholds:
Top 0.1%
Top 1%
Top 5%
Selection Tests:
AFD
BF
CLL
CLR
CSS
EHH
FST/di
HAPH
HMM−SFS
iES
iHS
Low MAF
Meta−SS
Omega
REHH
Rsb
SWAD
VarLD
XP−CLR
XP−EHH
ZHp

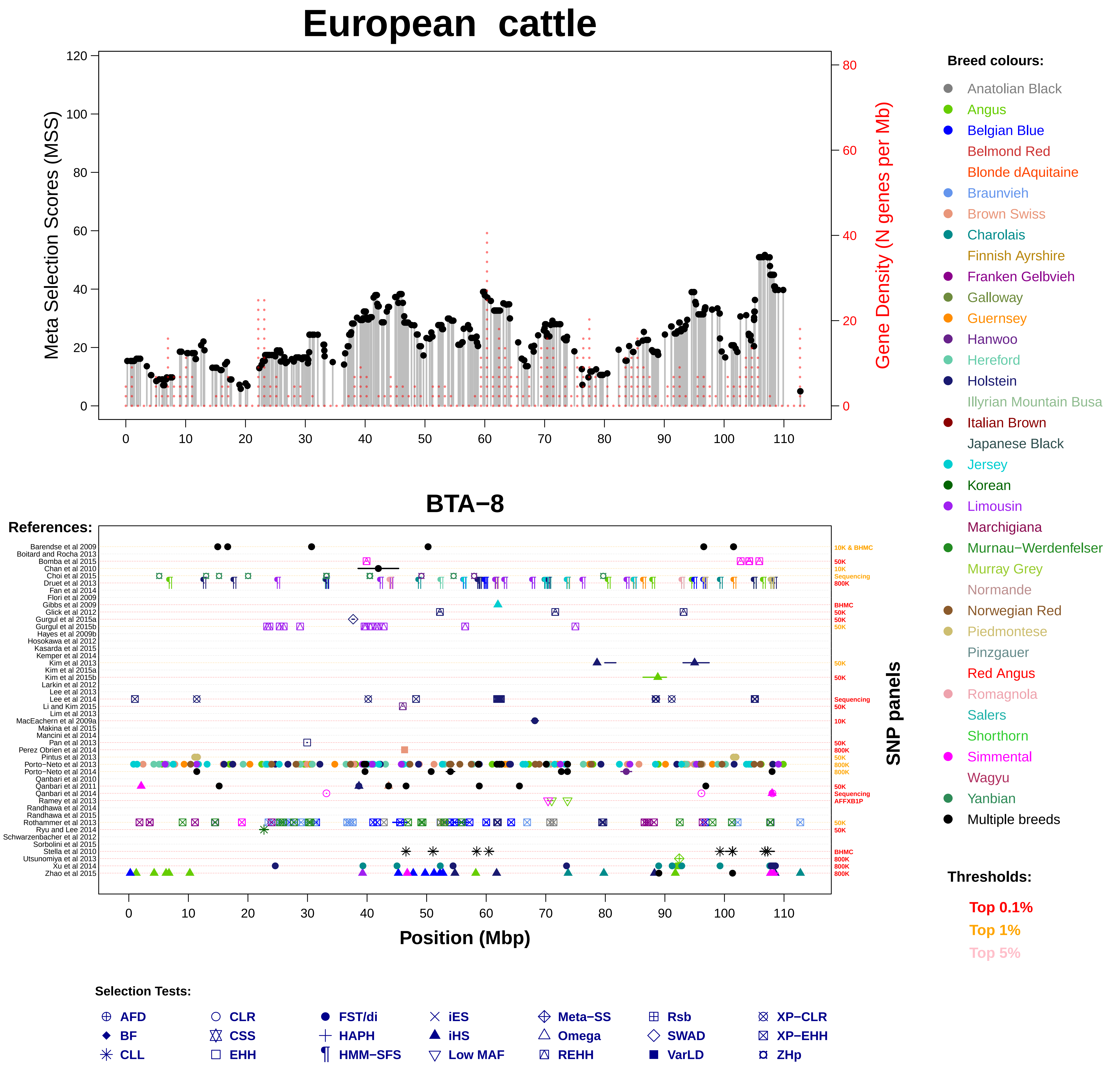

European cattle
Meta Selection Scores (MSS)
Gene Density (N genes per Mb)
BTA−8
References:
Barendse et al 2009
Boitard and Rocha 2013
Bomba et al 2015
Chan et al 2010
Choi et al 2015
Druet et al 2013
Fan et al 2014
Flori et al 2009
Gibbs et al 2009
Glick et al 2012
Gurgul et al 2015a
Gurgul et al 2015b
Hayes et al 2009b
Hosokawa et al 2012
Kasarda et al 2015
Kemper et al 2014
Kim et al 2013
Kim et al 2015a
Kim et al 2015b
Larkin et al 2012
Lee et al 2013
Lee et al 2014
Li and Kim 2015
Lim et al 2013
MacEachern et al 2009a
Makina et al 2015
Mancini et al 2014
Pan et al 2013
Perez Obrien et al 2014
Pintus et al 2013
Porto−Neto et al 2013
Porto−Neto et al 2014
Qanbari et al 2010
Qanbari et al 2011
Qanbari et al 2014
Ramey et al 2013
Randhawa et al 2014
Randhawa et al 2015
Rothammer et al 2013
Ryu and Lee 2014
Schwarzenbacher et al 2012
Sorbolini et al 2015
Stella et al 2010
Utsunomiya et al 2013
Xu et al 2014
Zhao et al 2015
10K & BHMC
50K
10K
Sequencing
800K
BHMC
AFFXB1P
SNP panels
Position (Mbp)
Breed colours:
Anatolian Black
Angus
Belgian Blue
Belmond Red
Blonde dAquitaine
Braunvieh
Brown Swiss
Charolais
Finnish Ayrshire
Franken Gelbvieh
Galloway
Guernsey
Hanwoo
Hereford
Holstein
Illyrian Mountain Busa
Italian Brown
Japanese Black
Jersey
Korean
Limousin
Marchigiana
Murnau−Werdenfelser
Murray Grey
Normande
Norwegian Red
Piedmontese
Pinzgauer
Red Angus
Romagnola
Salers
Shorthorn
Simmental
Wagyu
Yanbian
Multiple breeds
Thresholds:
Top 0.1%
Top 1%
Top 5%
Selection Tests:
AFD
BF
CLL
CLR
CSS
EHH
FST/di
HAPH
HMM−SFS
iES
iHS
Low MAF
Meta−SS
Omega
REHH
Rsb
SWAD
VarLD
XP−CLR
XP−EHH
ZHp

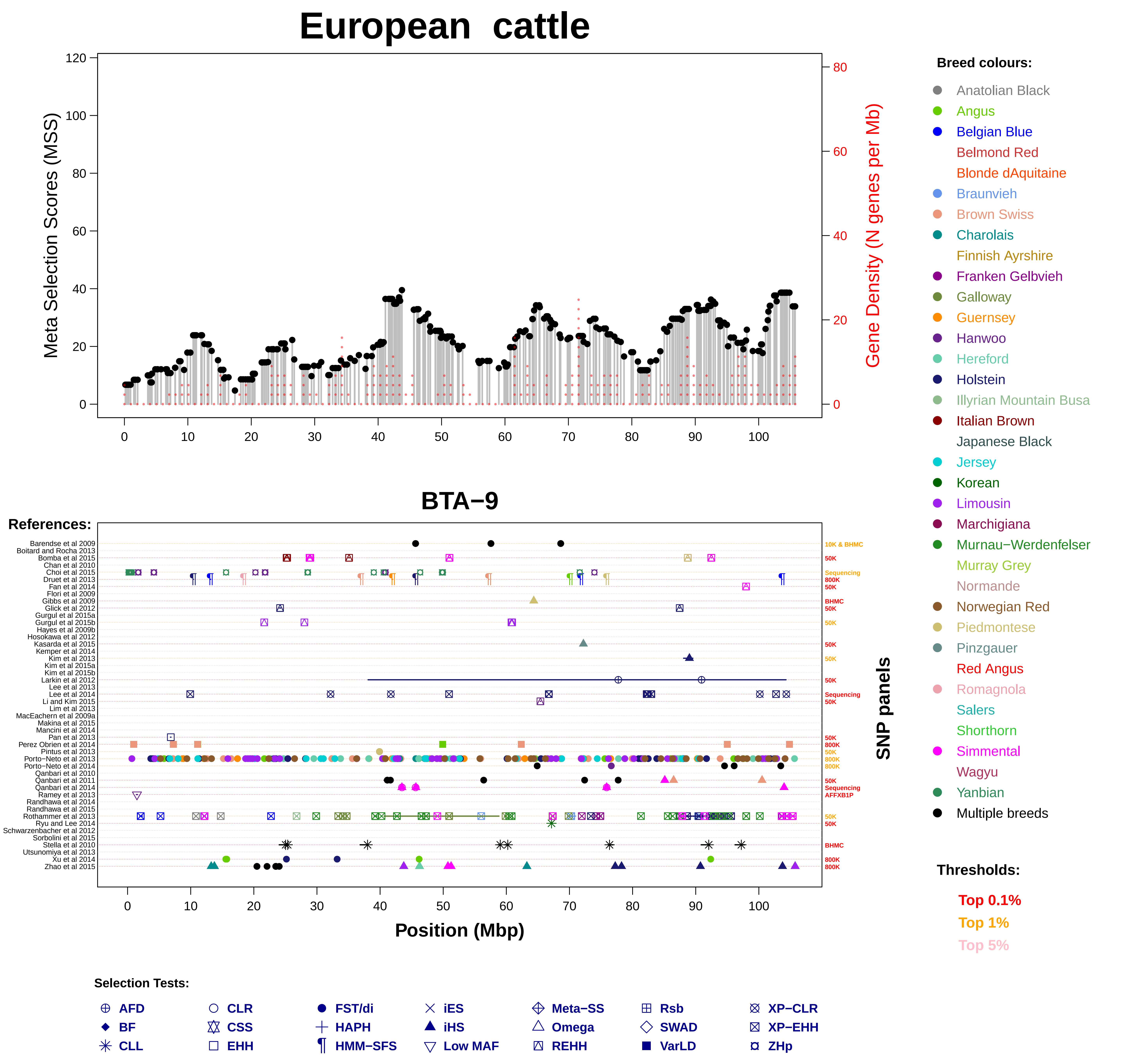
European cattle
Meta Selection Scores (MSS)
Gene Density (N genes per Mb)
BTA−9
References:
Barendse et al 2009
Boitard and Rocha 2013
Bomba et al 2015
Chan et al 2010
Choi et al 2015
Druet et al 2013
Fan et al 2014
Flori et al 2009
Gibbs et al 2009
Glick et al 2012
Gurgul et al 2015a
Gurgul et al 2015b
Hayes et al 2009b
Hosokawa et al 2012
Kasarda et al 2015
Kemper et al 2014
Kim et al 2013
Kim et al 2015a
Kim et al 2015b
Larkin et al 2012
Lee et al 2013
Lee et al 2014
Li and Kim 2015
Lim et al 2013
MacEachern et al 2009a
Makina et al 2015
Mancini et al 2014
Pan et al 2013
Perez Obrien et al 2014
Pintus et al 2013
Porto−Neto et al 2013
Porto−Neto et al 2014
Qanbari et al 2010
Qanbari et al 2011
Qanbari et al 2014
Ramey et al 2013
Randhawa et al 2014
Randhawa et al 2015
Rothammer et al 2013
Ryu and Lee 2014
Schwarzenbacher et al 2012
Sorbolini et al 2015
Stella et al 2010
Utsunomiya et al 2013
Xu et al 2014
Zhao et al 2015
10K & BHMC
50K
Sequencing
800K
50K
BHMC
50K
50K
50K
50K
50K
Sequencing
50K
50K
800K
50K
800K
800K
50K
Sequencing
AFFXB1P
50K
50K
BHMC
800K
800K
SNP panels
Position (Mbp)
Breed colours:
Anatolian Black
Angus
Belgian Blue
Belmond Red
Blonde dAquitaine
Braunvieh
Brown Swiss
Charolais
Finnish Ayrshire
Franken Gelbvieh
Galloway
Guernsey
Hanwoo
Hereford
Holstein
Illyrian Mountain Busa
Italian Brown
Japanese Black
Jersey
Korean
Limousin
Marchigiana
Murnau−Werdenfelser
Murray Grey
Normande
Norwegian Red
Piedmontese
Pinzgauer
Red Angus
Romagnola
Salers
Shorthorn
Simmental
Wagyu
Yanbian
Multiple breeds
Thresholds:
Top 0.1%
Top 1%
Top 5%
Selection Tests:
AFD
BF
CLL
CLR
CSS
EHH
FST/di
HAPH
HMM−SFS
iES
iHS
Low MAF
Meta−SS
Omega
REHH
Rsb
SWAD
VarLD
XP−CLR
XP−EHH
ZHp

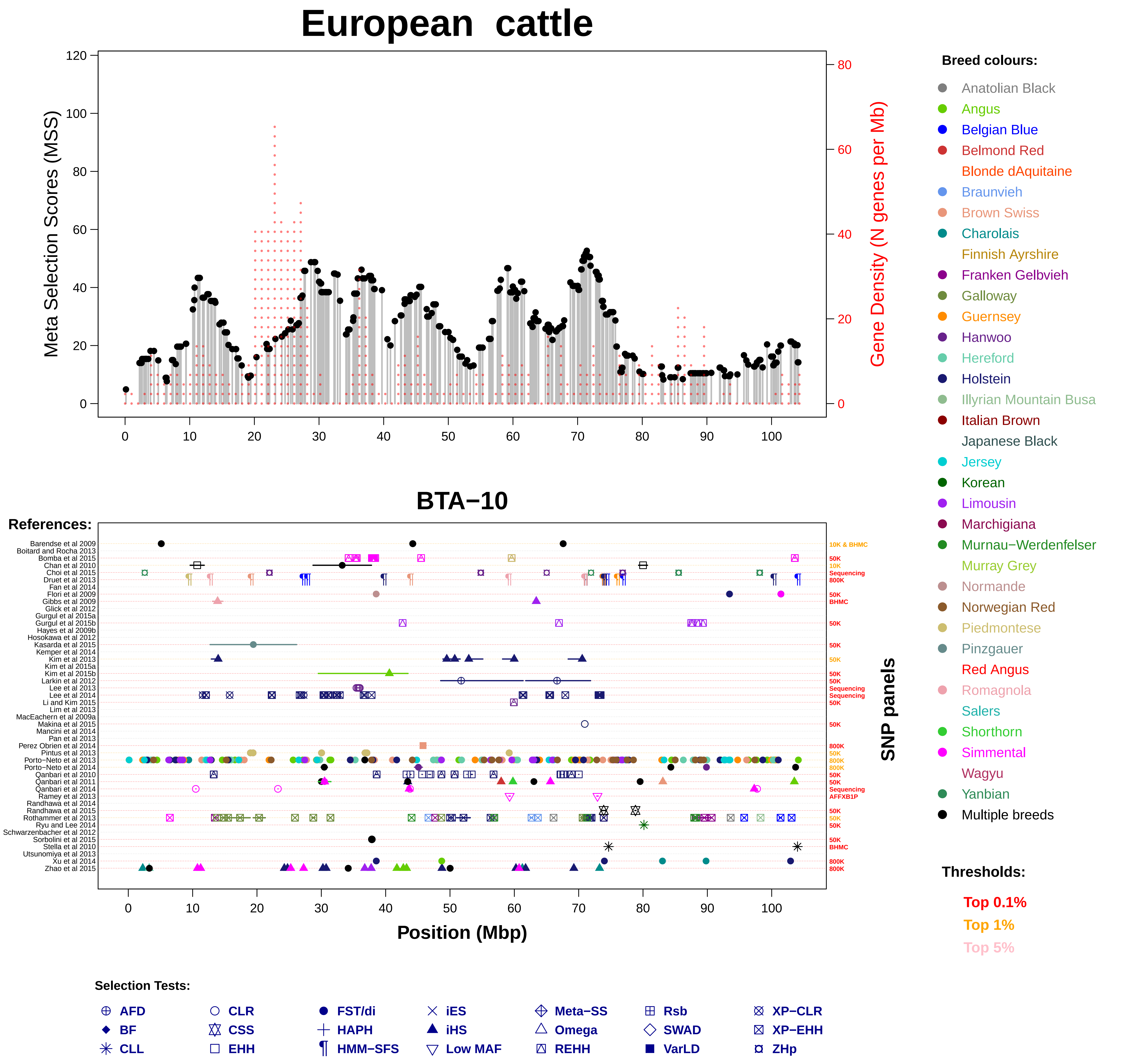

European cattle
Meta Selection Scores (MSS)
Gene Density (N genes per Mb)
BTA−10
References:
Barendse et al 2009
Boitard and Rocha 2013
Bomba et al 2015
Chan et al 2010
Choi et al 2015
Druet et al 2013
Fan et al 2014
Flori et al 2009
Gibbs et al 2009
Glick et al 2012
Gurgul et al 2015a
Gurgul et al 2015b
Hayes et al 2009b
Hosokawa et al 2012
Kasarda et al 2015
Kemper et al 2014
Kim et al 2013
Kim et al 2015a
Kim et al 2015b
Larkin et al 2012
Lee et al 2013
Lee et al 2014
Li and Kim 2015
Lim et al 2013
MacEachern et al 2009a
Makina et al 2015
Mancini et al 2014
Pan et al 2013
Perez Obrien et al 2014
Pintus et al 2013
Porto−Neto et al 2013
Porto−Neto et al 2014
Qanbari et al 2010
Qanbari et al 2011
Qanbari et al 2014
Ramey et al 2013
Randhawa et al 2014
Randhawa et al 2015
Rothammer et al 2013
Ryu and Lee 2014
Schwarzenbacher et al 2012
Sorbolini et al 2015
Stella et al 2010
Utsunomiya et al 2013
Xu et al 2014
Zhao et al 2015
10K & BHMC
50K
10K
Sequencing
800K
BHMC
AFFXB1P
SNP panels
Position (Mbp)
Breed colours:
Anatolian Black
Angus
Belgian Blue
Belmond Red
Blonde dAquitaine
Braunvieh
Brown Swiss
Charolais
Finnish Ayrshire
Franken Gelbvieh
Galloway
Guernsey
Hanwoo
Hereford
Holstein
Illyrian Mountain Busa
Italian Brown
Japanese Black
Jersey
Korean
Limousin
Marchigiana
Murnau−Werdenfelser
Murray Grey
Normande
Norwegian Red
Piedmontese
Pinzgauer
Red Angus
Romagnola
Salers
Shorthorn
Simmental
Wagyu
Yanbian
Multiple breeds
Thresholds:
Top 0.1%
Top 1%
Top 5%
Selection Tests:
AFD
BF
CLL
CLR
CSS
EHH
FST/di
HAPH
HMM−SFS
iES
iHS
Low MAF
Meta−SS
Omega
REHH
Rsb
SWAD
VarLD
XP−CLR
XP−EHH
ZHp

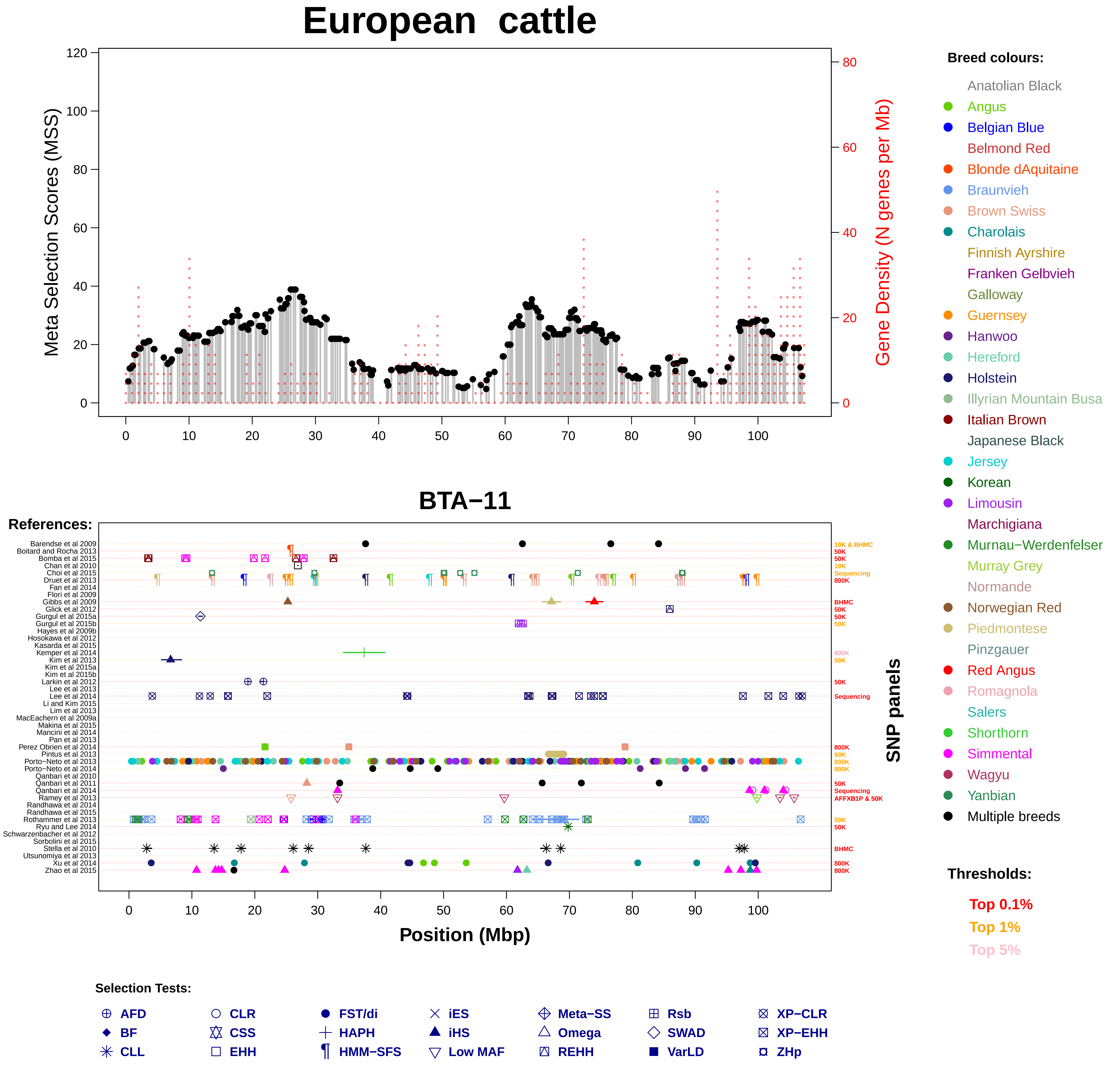

European cattle
Meta Selection Scores (MSS)
Gene Density (N genes per Mb)
BTA−11
References:
Barendse et al 2009
Boitard and Rocha 2013
Bomba et al 2015
Chan et al 2010
Choi et al 2015
Druet et al 2013
Fan et al 2014
Flori et al 2009
Gibbs et al 2009
Glick et al 2012
Gurgul et al 2015a
Gurgul et al 2015b
Hayes et al 2009b
Hosokawa et al 2012
Kasarda et al 2015
Kemper et al 2014
Kim et al 2013
Kim et al 2015a
Kim et al 2015b
Larkin et al 2012
Lee et al 2013
Lee et al 2014
Li and Kim 2015
Lim et al 2013
MacEachern et al 2009a
Makina et al 2015
Mancini et al 2014
Pan et al 2013
Perez Obrien et al 2014
Pintus et al 2013
Porto−Neto et al 2013
Porto−Neto et al 2014
Qanbari et al 2010
Qanbari et al 2011
Qanbari et al 2014
Ramey et al 2013
Randhawa et al 2014
Randhawa et al 2015
Rothammer et al 2013
Ryu and Lee 2014
Schwarzenbacher et al 2012
Sorbolini et al 2015
Stella et al 2010
Utsunomiya et al 2013
Xu et al 2014
Zhao et al 2015
10K & BHMC
50K
50K
10K
Sequencing
800K
BHMC
50K
50K
50K
800K
50K
50K
Sequencing
800K
50K
800K
800K
50K
Sequencing
AFFXB1P & 50K
50K
50K
BHMC
800K
800K
SNP panels
Position (Mbp)
Breed colours:
Anatolian Black
Angus
Belgian Blue
Belmond Red
Blonde dAquitaine
Braunvieh
Brown Swiss
Charolais
Finnish Ayrshire
Franken Gelbvieh
Galloway
Guernsey
Hanwoo
Hereford
Holstein
Illyrian Mountain Busa
Italian Brown
Japanese Black
Jersey
Korean
Limousin
Marchigiana
Murnau−Werdenfelser
Murray Grey
Normande
Norwegian Red
Piedmontese
Pinzgauer
Red Angus
Romagnola
Salers
Shorthorn
Simmental
Wagyu
Yanbian
Multiple breeds
Thresholds:
Top 0.1%
Top 1%
Top 5%
Selection Tests:
AFD
BF
CLL
CLR
CSS
EHH
FST/di
HAPH
HMM−SFS
iES
iHS
Low MAF
Meta−SS
Omega
REHH
Rsb
SWAD
VarLD
XP−CLR
XP−EHH
ZHp

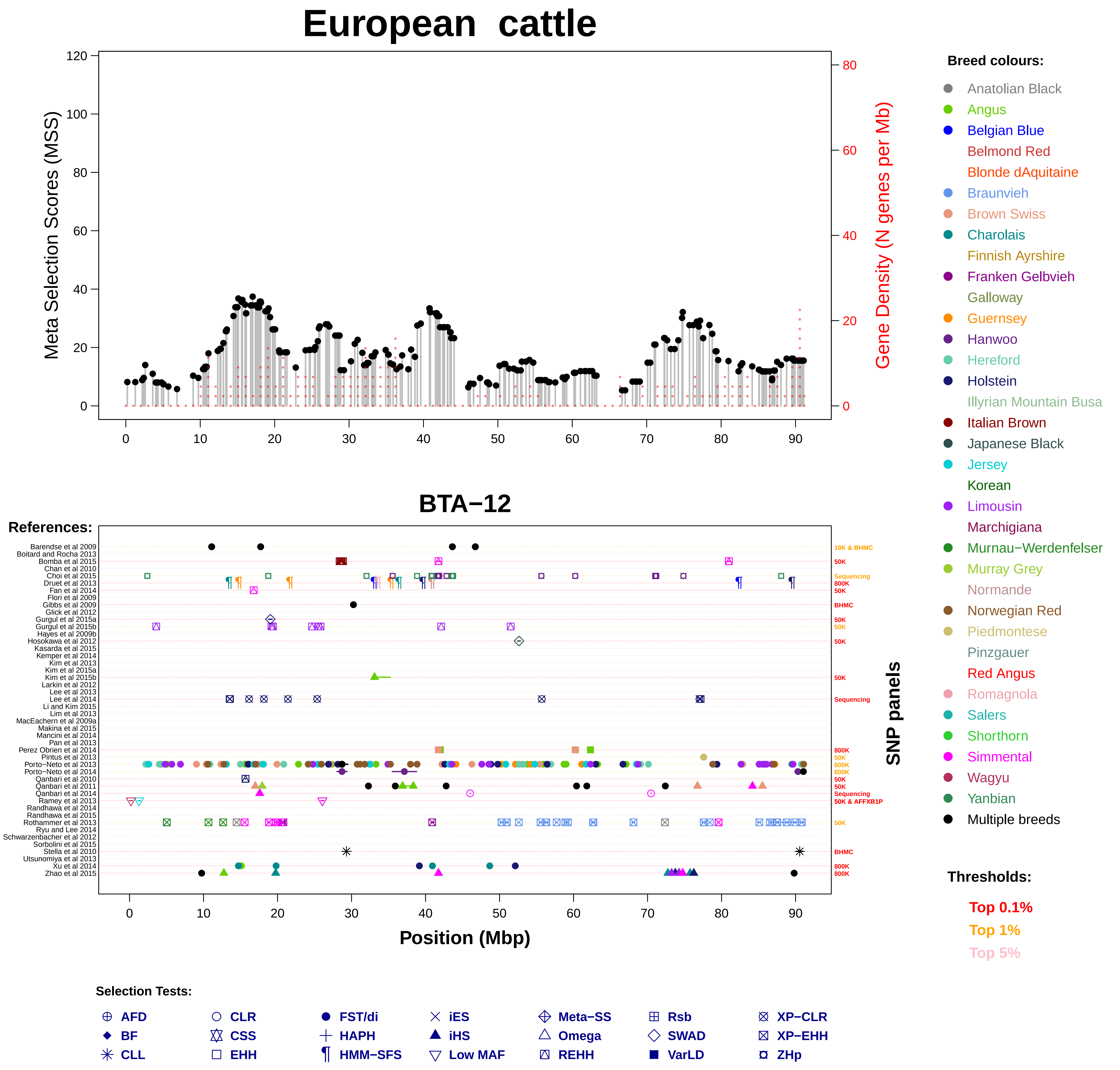
European cattle
Meta Selection Scores (MSS)
Gene Density (N genes per Mb)
BTA−12
References:
Barendse et al 2009
Boitard and Rocha 2013
Bomba et al 2015
Chan et al 2010
Choi et al 2015
Druet et al 2013
Fan et al 2014
Flori et al 2009
Gibbs et al 2009
Glick et al 2012
Gurgul et al 2015a
Gurgul et al 2015b
Hayes et al 2009b
Hosokawa et al 2012
Kasarda et al 2015
Kemper et al 2014
Kim et al 2013
Kim et al 2015a
Kim et al 2015b
Larkin et al 2012
Lee et al 2013
Lee et al 2014
Li and Kim 2015
Lim et al 2013
MacEachern et al 2009a
Makina et al 2015
Mancini et al 2014
Pan et al 2013
Perez Obrien et al 2014
Pintus et al 2013
Porto−Neto et al 2013
Porto−Neto et al 2014
Qanbari et al 2010
Qanbari et al 2011
Qanbari et al 2014
Ramey et al 2013
Randhawa et al 2014
Randhawa et al 2015
Rothammer et al 2013
Ryu and Lee 2014
Schwarzenbacher et al 2012
Sorbolini et al 2015
Stella et al 2010
Utsunomiya et al 2013
Xu et al 2014
Zhao et al 2015
SNP panels
10K & BHMC
50K
Sequencing
800K
50K
BHMC
50K
50K
50K
50K
Sequencing
800K
50K
800K
800K
50K
50K
Sequencing
50K & AFFXB1P
50K
BHMC
800K
800K
Position (Mbp)
Breed colours:
Anatolian Black
Angus
Belgian Blue
Belmond Red
Blonde dAquitaine
Braunvieh
Brown Swiss
Charolais
Finnish Ayrshire
Franken Gelbvieh
Galloway
Guernsey
Hanwoo
Hereford
Holstein
Illyrian Mountain Busa
Italian Brown
Japanese Black
Jersey
Korean
Limousin
Marchigiana
Murnau−Werdenfelser
Murray Grey
Normande
Norwegian Red
Piedmontese
Pinzgauer
Red Angus
Romagnola
Salers
Shorthorn
Simmental
Wagyu
Yanbian
Multiple breeds
Thresholds:
Top 0.1%
Top 1%
Top 5%
Selection Tests:
AFD
BF
CLL
CLR
CSS
EHH
FST/di
HAPH
HMM−SFS
iES
iHS
Low MAF
Meta−SS
Omega
REHH
Rsb
SWAD
VarLD
XP−CLR
XP−EHH
ZHp

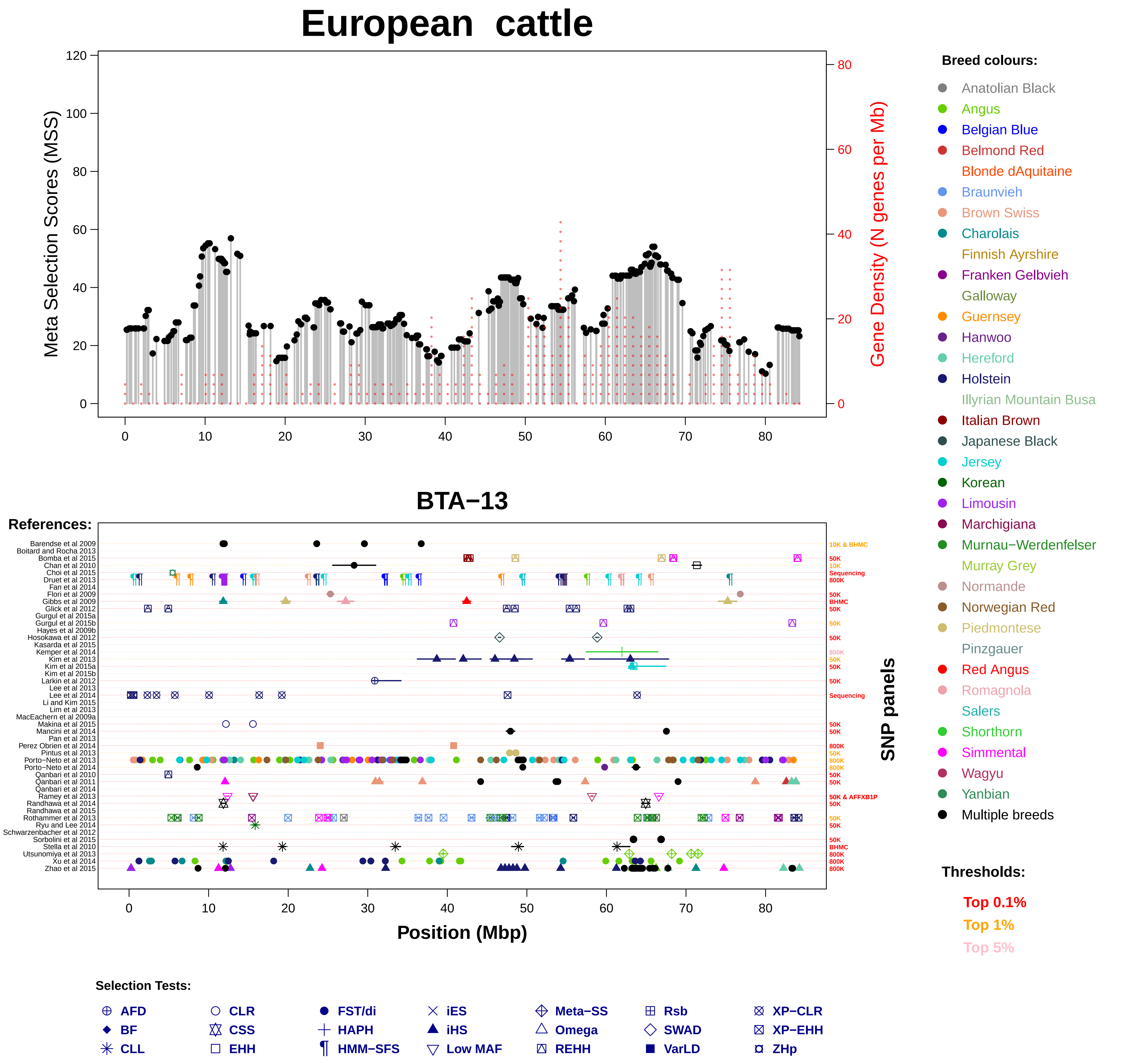
European cattle
Meta Selection Scores (MSS)
Gene Density (N genes per Mb)
BTA−13
References:
Barendse et al 2009
Boitard and Rocha 2013
Bomba et al 2015
Chan et al 2010
Choi et al 2015
Druet et al 2013
Fan et al 2014
Flori et al 2009
Gibbs et al 2009
Glick et al 2012
Gurgul et al 2015a
Gurgul et al 2015b
Hayes et al 2009b
Hosokawa et al 2012
Kasarda et al 2015
Kemper et al 2014
Kim et al 2013
Kim et al 2015a
Kim et al 2015b
Larkin et al 2012
Lee et al 2013
Lee et al 2014
Li and Kim 2015
Lim et al 2013
MacEachern et al 2009a
Makina et al 2015
Mancini et al 2014
Pan et al 2013
Perez Obrien et al 2014
Pintus et al 2013
Porto−Neto et al 2013
Porto−Neto et al 2014
Qanbari et al 2010
Qanbari et al 2011
Qanbari et al 2014
Ramey et al 2013
Randhawa et al 2014
Randhawa et al 2015
Rothammer et al 2013
Ryu and Lee 2014
Schwarzenbacher et al 2012
Sorbolini et al 2015
Stella et al 2010
Utsunomiya et al 2013
Xu et al 2014
Zhao et al 2015
10K & BHMC
50K
10K
Sequencing
800K
50K
BHMC
50K
50K
50K
800K
50K
50K
50K
Sequencing
50K
50K
800K
50K
800K
800K
50K
50K
50K & AFFXB1P
50K
50K
50K
50K
BHMC
800K
800K
800K
SNP panels
Position (Mbp)
Breed colours:
Anatolian Black
Angus
Belgian Blue
Belmond Red
Blonde dAquitaine
Braunvieh
Brown Swiss
Charolais
Finnish Ayrshire
Franken Gelbvieh
Galloway
Guernsey
Hanwoo
Hereford
Holstein
Illyrian Mountain Busa
Italian Brown
Japanese Black
Jersey
Korean
Limousin
Marchigiana
Murnau−Werdenfelser
Murray Grey
Normande
Norwegian Red
Piedmontese
Pinzgauer
Red Angus
Romagnola
Salers
Shorthorn
Simmental
Wagyu
Yanbian
Multiple breeds
Thresholds:
Top 0.1%
Top 1%
Top 5%
Selection Tests:
AFD
BF
CLL
CLR
CSS
EHH
FST/di
HAPH
HMM−SFS
iES
iHS
Low MAF
Meta−SS
Omega
REHH
Rsb
SWAD
VarLD
XP−CLR
XP−EHH
ZHp

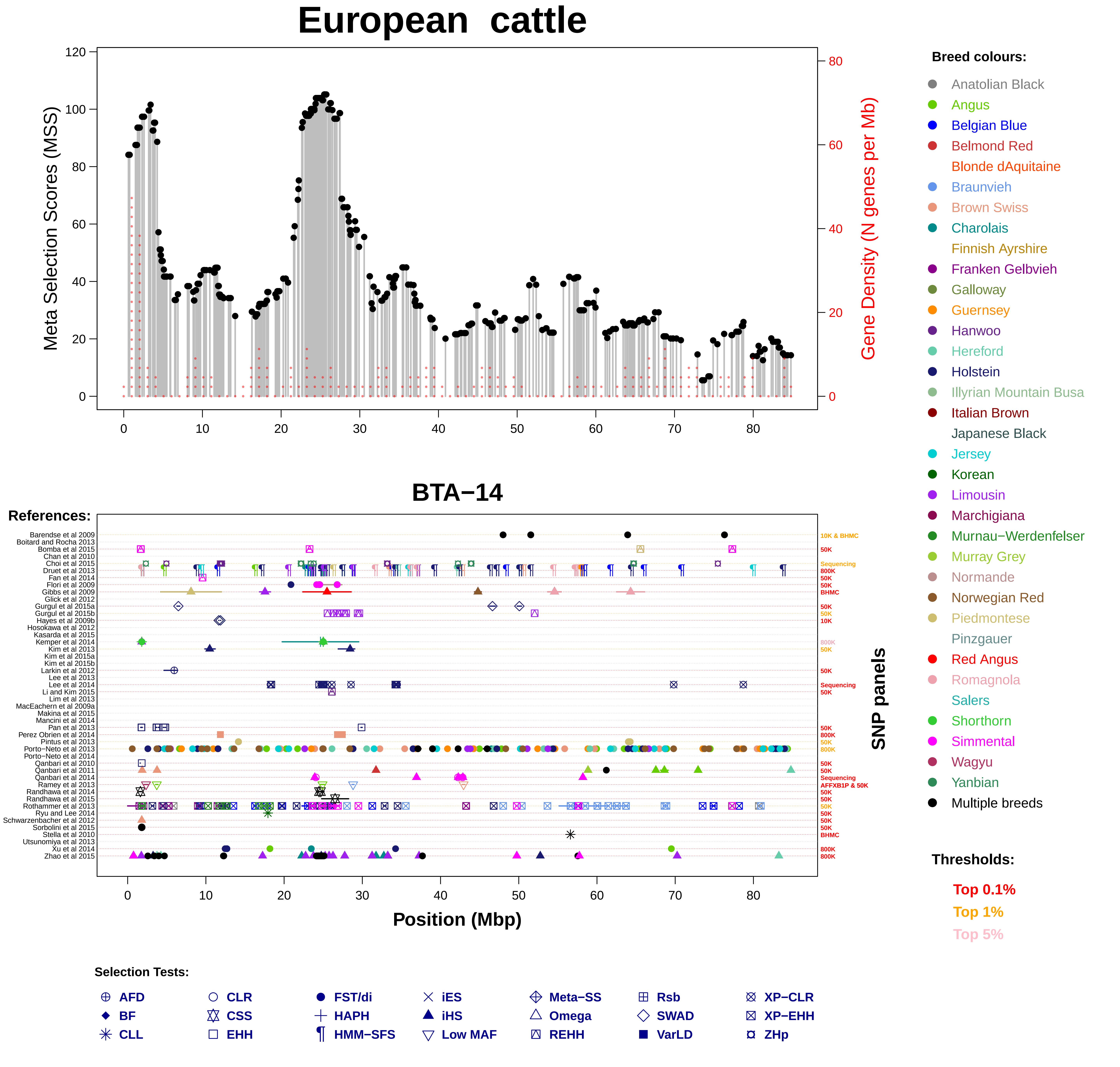

European cattle
Meta Selection Scores (MSS)
Gene Density (N genes per Mb)
BTA−14
References:
SNP panels
Position (Mbp)
Breed colours:
Anatolian Black
Angus
Belgian Blue
Belmond Red
Blonde dAquitaine
Braunvieh
Brown Swiss
Charolais
Finnish Ayrshire
Franken Gelbvieh
Galloway
Guernsey
Hanwoo
Hereford
Holstein
Illyrian Mountain Busa
Italian Brown
Japanese Black
Jersey
Korean
Limousin
Marchigiana
Murnau−Werdenfelser
Murray Grey
Normande
Norwegian Red
Piedmontese
Pinzgauer
Red Angus
Romagnola
Salers
Shorthorn
Simmental
Wagyu
Yanbian
Multiple breeds
Thresholds:
Top 0.1%
Top 1%
Top 5%
Selection Tests:
AFD
BF
CLL
CLR
CSS
EHH
FST/di
HAPH
HMM−SFS
iES
iHS
Low MAF
Meta−SS
Omega
REHH
Rsb
SWAD
VarLD
XP−CLR
XP−EHH
ZHp

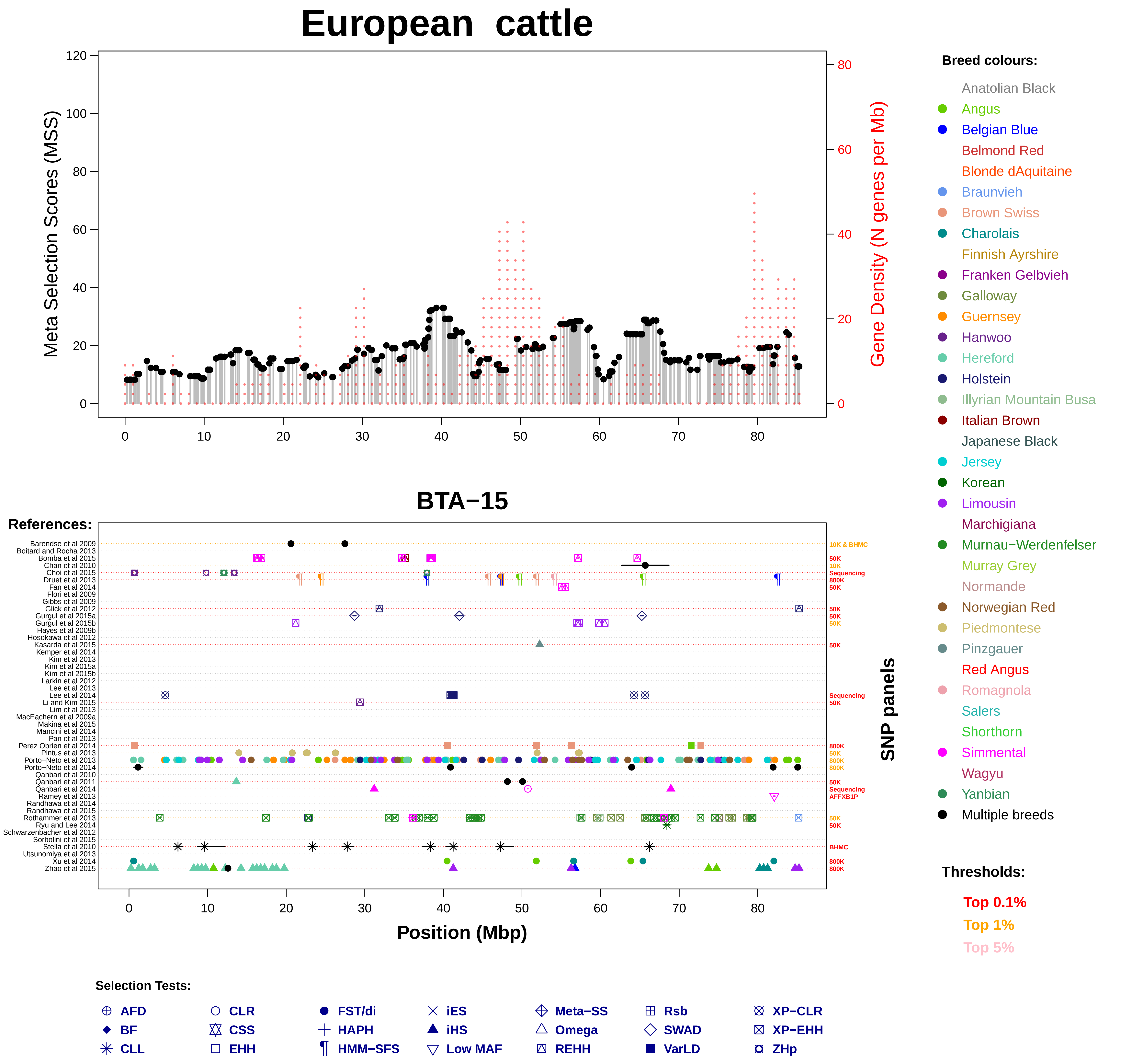
European cattle
Meta Selection Scores (MSS)
Gene Density (N genes per Mb)
BTA−15
References:
Barendse et al 2009
Boitard and Rocha 2013
Bomba et al 2015
Chan et al 2010
Choi et al 2015
Druet et al 2013
Fan et al 2014
Flori et al 2009
Gibbs et al 2009
Glick et al 2012
Gurgul et al 2015a
Gurgul et al 2015b
Hayes et al 2009b
Hosokawa et al 2012
Kasarda et al 2015
Kemper et al 2014
Kim et al 2013
Kim et al 2015a
Kim et al 2015b
Larkin et al 2012
Lee et al 2013
Lee et al 2014
Li and Kim 2015
Lim et al 2013
MacEachern et al 2009a
Makina et al 2015
Mancini et al 2014
Pan et al 2013
Perez Obrien et al 2014
Pintus et al 2013
Porto−Neto et al 2013
Porto−Neto et al 2014
Qanbari et al 2010
Qanbari et al 2011
Qanbari et al 2014
Ramey et al 2013
Randhawa et al 2014
Randhawa et al 2015
Rothammer et al 2013
Ryu and Lee 2014
Schwarzenbacher et al 2012
Sorbolini et al 2015
Stella et al 2010
Utsunomiya et al 2013
Xu et al 2014
Zhao et al 2015
SNP panels
10K & BHMC
50K
10K
Sequencing
800K
50K
50K
50K
50K
50K
Sequencing
50K
800K
50K
800K
800K
50K
Sequencing
AFFXB1P
50K
50K
BHMC
800K
800K
Position (Mbp)
Breed colours:
Anatolian Black
Angus
Belgian Blue
Belmond Red
Blonde dAquitaine
Braunvieh
Brown Swiss
Charolais
Finnish Ayrshire
Franken Gelbvieh
Galloway
Guernsey
Hanwoo
Hereford
Holstein
Illyrian Mountain Busa
Italian Brown
Japanese Black
Jersey
Korean
Limousin
Marchigiana
Murnau−Werdenfelser
Murray Grey
Normande
Norwegian Red
Piedmontese
Pinzgauer
Red Angus
Romagnola
Salers
Shorthorn
Simmental
Wagyu
Yanbian
Multiple breeds
Thresholds:
Top 0.1%
Top 1%
Top 5%
Selection Tests:
AFD
BF
CLL
CLR
CSS
EHH
FST/di
HAPH
HMM−SFS
iES
iHS
Low MAF
Meta−SS
Omega
REHH
Rsb
SWAD
VarLD
XP−CLR
XP−EHH
ZHp

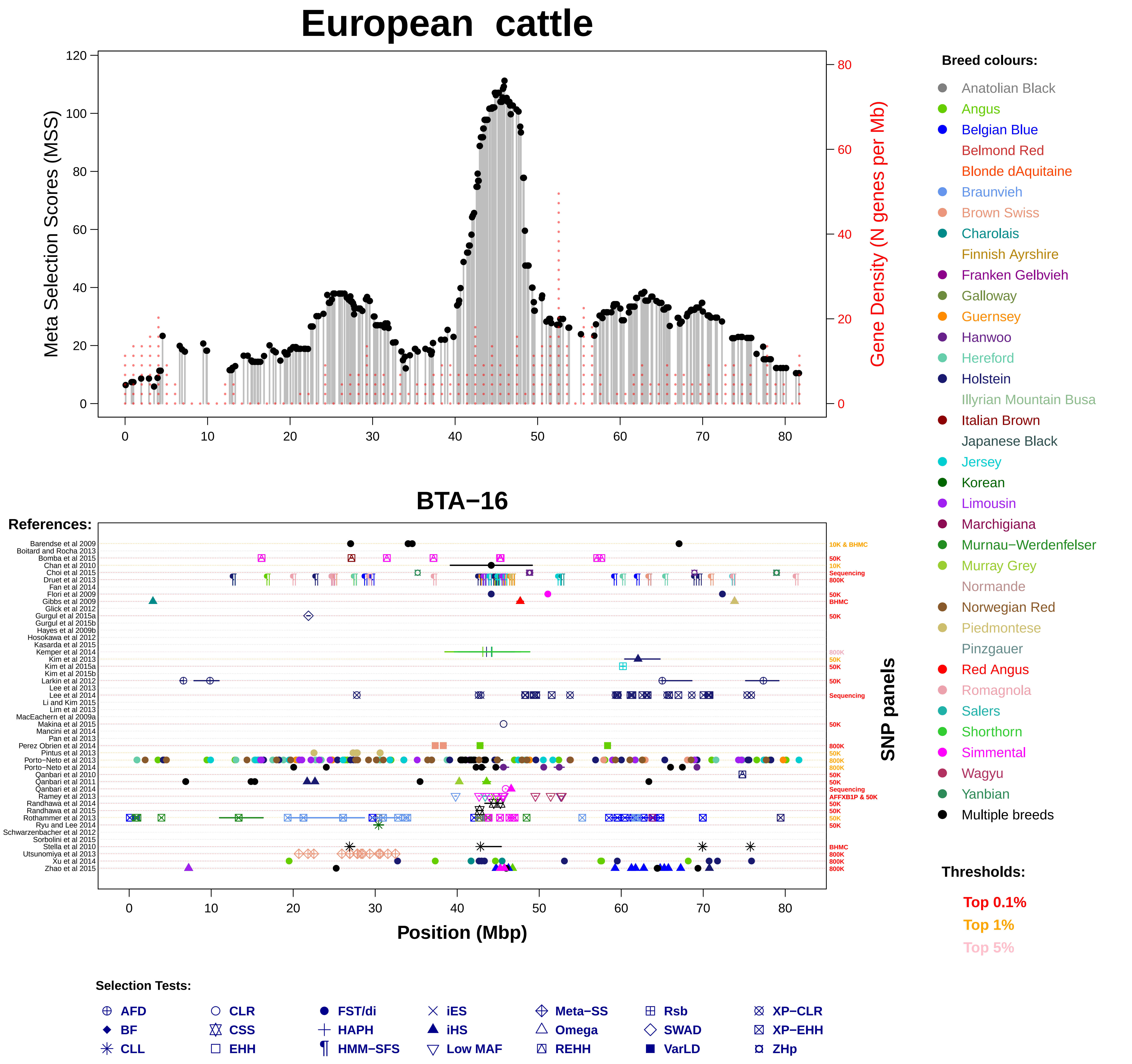
European cattle
Meta Selection Scores (MSS)
Gene Density (N genes per Mb)
BTA−16
References:
Barendse et al 2009
Boitard and Rocha 2013
Bomba et al 2015
Chan et al 2010
Choi et al 2015
Druet et al 2013
Fan et al 2014
Flori et al 2009
Gibbs et al 2009
Glick et al 2012
Gurgul et al 2015a
Gurgul et al 2015b
Hayes et al 2009b
Hosokawa et al 2012
Kasarda et al 2015
Kemper et al 2014
Kim et al 2013
Kim et al 2015a
Kim et al 2015b
Larkin et al 2012
Lee et al 2013
Lee et al 2014
Li and Kim 2015
Lim et al 2013
MacEachern et al 2009a
Makina et al 2015
Mancini et al 2014
Pan et al 2013
Perez Obrien et al 2014
Pintus et al 2013
Porto−Neto et al 2013
Porto−Neto et al 2014
Qanbari et al 2010
Qanbari et al 2011
Qanbari et al 2014
Ramey et al 2013
Randhawa et al 2014
Randhawa et al 2015
Rothammer et al 2013
Ryu and Lee 2014
Schwarzenbacher et al 2012
Sorbolini et al 2015
Stella et al 2010
Utsunomiya et al 2013
Xu et al 2014
Zhao et al 2015
SNP panels
Position (Mbp)
Breed colours:
Anatolian Black
Angus
Belgian Blue
Belmond Red
Blonde dAquitaine
Braunvieh
Brown Swiss
Charolais
Finnish Ayrshire
Franken Gelbvieh
Galloway
Guernsey
Hanwoo
Hereford
Holstein
Illyrian Mountain Busa
Italian Brown
Japanese Black
Jersey
Korean
Limousin
Marchigiana
Murnau−Werdenfelser
Murray Grey
Normande
Norwegian Red
Piedmontese
Pinzgauer
Red Angus
Romagnola
Salers
Shorthorn
Simmental
Wagyu
Yanbian
Multiple breeds
Thresholds:
Top 0.1%
Top 1%
Top 5%
Selection Tests:
AFD
BF
CLL
CLR
CSS
EHH
FST/di
HAPH
HMM−SFS
iES
iHS
Low MAF
Meta−SS
Omega
REHH
Rsb
SWAD
VarLD
XP−CLR
XP−EHH
ZHp

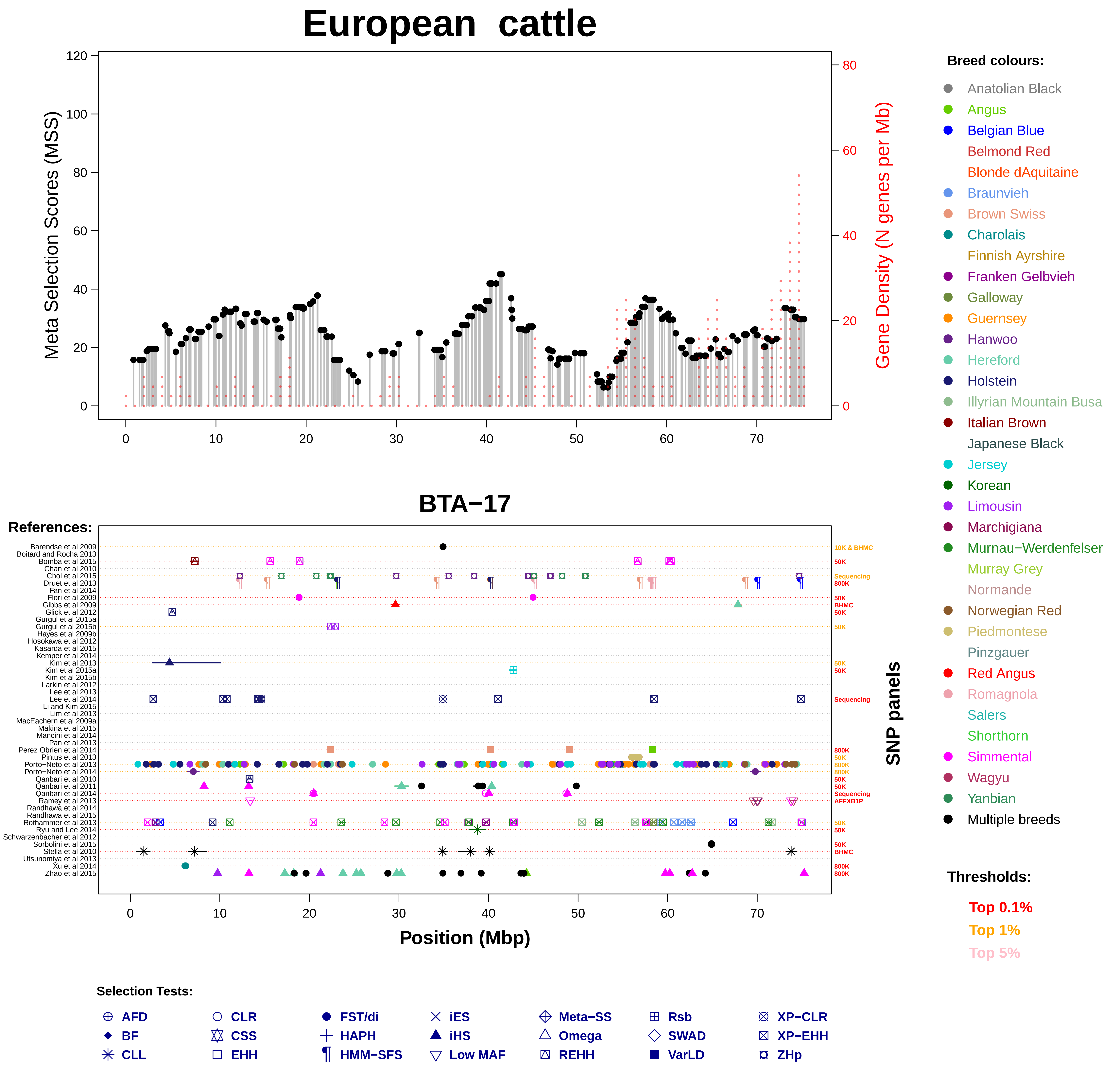

European cattle
Meta Selection Scores (MSS)
Gene Density (N genes per Mb)
BTA−17
References:
Barendse et al 2009
Boitard and Rocha 2013
Bomba et al 2015
Chan et al 2010
Choi et al 2015
Druet et al 2013
Fan et al 2014
Flori et al 2009
Gibbs et al 2009
Glick et al 2012
Gurgul et al 2015a
Gurgul et al 2015b
Hayes et al 2009b
Hosokawa et al 2012
Kasarda et al 2015
Kemper et al 2014
Kim et al 2013
Kim et al 2015a
Kim et al 2015b
Larkin et al 2012
Lee et al 2013
Lee et al 2014
Li and Kim 2015
Lim et al 2013
MacEachern et al 2009a
Makina et al 2015
Mancini et al 2014
Pan et al 2013
Perez Obrien et al 2014
Pintus et al 2013
Porto−Neto et al 2013
Porto−Neto et al 2014
Qanbari et al 2010
Qanbari et al 2011
Qanbari et al 2014
Ramey et al 2013
Randhawa et al 2014
Randhawa et al 2015
Rothammer et al 2013
Ryu and Lee 2014
Schwarzenbacher et al 2012
Sorbolini et al 2015
Stella et al 2010
Utsunomiya et al 2013
Xu et al 2014
Zhao et al 2015
Position (Mbp)
SNP panels
10K & BHMC
50K
Sequencing
800K
BHMC
AFFXB1P
Breed colours:
Anatolian Black
Angus
Belgian Blue
Belmond Red
Blonde dAquitaine
Braunvieh
Brown Swiss
Charolais
Finnish Ayrshire
Franken Gelbvieh
Galloway
Guernsey
Hanwoo
Hereford
Holstein
Illyrian Mountain Busa
Italian Brown
Japanese Black
Jersey
Korean
Limousin
Marchigiana
Murnau−Werdenfelser
Murray Grey
Normande
Norwegian Red
Piedmontese
Pinzgauer
Red Angus
Romagnola
Salers
Shorthorn
Simmental
Wagyu
Yanbian
Multiple breeds
Thresholds:
Top 0.1%
Top 1%
Top 5%
Selection Tests:
AFD
BF
CLL
CLR
CSS
EHH
FST/di
HAPH
HMM−SFS
iES
iHS
Low MAF
Meta−SS
Omega
REHH
Rsb
SWAD
VarLD
XP−CLR
XP−EHH
ZHp

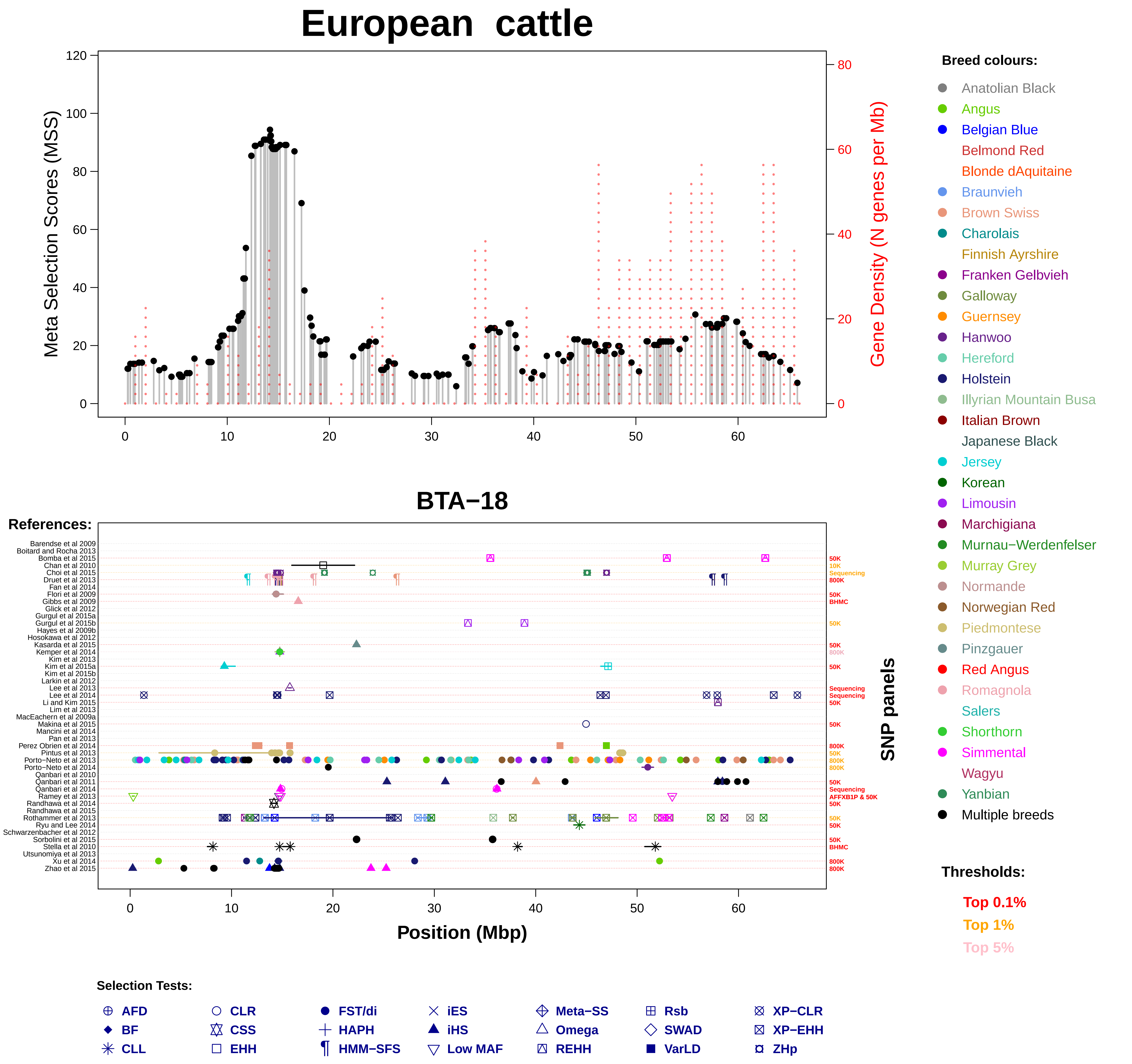

European cattle
Meta Selection Scores (MSS)
Gene Density (N genes per Mb)
BTA−18
References:
Barendse et al 2009
Boitard and Rocha 2013
Bomba et al 2015
Chan et al 2010
Choi et al 2015
Druet et al 2013
Fan et al 2014
Flori et al 2009
Gibbs et al 2009
Glick et al 2012
Gurgul et al 2015a
Gurgul et al 2015b
Hayes et al 2009b
Hosokawa et al 2012
Kasarda et al 2015
Kemper et al 2014
Kim et al 2013
Kim et al 2015a
Kim et al 2015b
Larkin et al 2012
Lee et al 2013
Lee et al 2014
Li and Kim 2015
Lim et al 2013
MacEachern et al 2009a
Makina et al 2015
Mancini et al 2014
Pan et al 2013
Perez Obrien et al 2014
Pintus et al 2013
Porto−Neto et al 2013
Porto−Neto et al 2014
Qanbari et al 2010
Qanbari et al 2011
Qanbari et al 2014
Ramey et al 2013
Randhawa et al 2014
Randhawa et al 2015
Rothammer et al 2013
Ryu and Lee 2014
Schwarzenbacher et al 2012
Sorbolini et al 2015
Stella et al 2010
Utsunomiya et al 2013
Xu et al 2014
Zhao et al 2015
SNP panels
Position (Mbp)
Breed colours:
Anatolian Black
Angus
Belgian Blue
Belmond Red
Blonde dAquitaine
Braunvieh
Brown Swiss
Charolais
Finnish Ayrshire
Franken Gelbvieh
Galloway
Guernsey
Hanwoo
Hereford
Holstein
Illyrian Mountain Busa
Italian Brown
Japanese Black
Jersey
Korean
Limousin
Marchigiana
Murnau−Werdenfelser
Murray Grey
Normande
Norwegian Red
Piedmontese
Pinzgauer
Red Angus
Romagnola
Salers
Shorthorn
Simmental
Wagyu
Yanbian
Multiple breeds
Thresholds:
Top 0.1%
Top 1%
Top 5%
Selection Tests:
AFD
BF
CLL
CLR
CSS
EHH
FST/di
HAPH
HMM−SFS
iES
iHS
Low MAF
Meta−SS
Omega
REHH
Rsb
SWAD
VarLD
XP−CLR
XP−EHH
ZHp

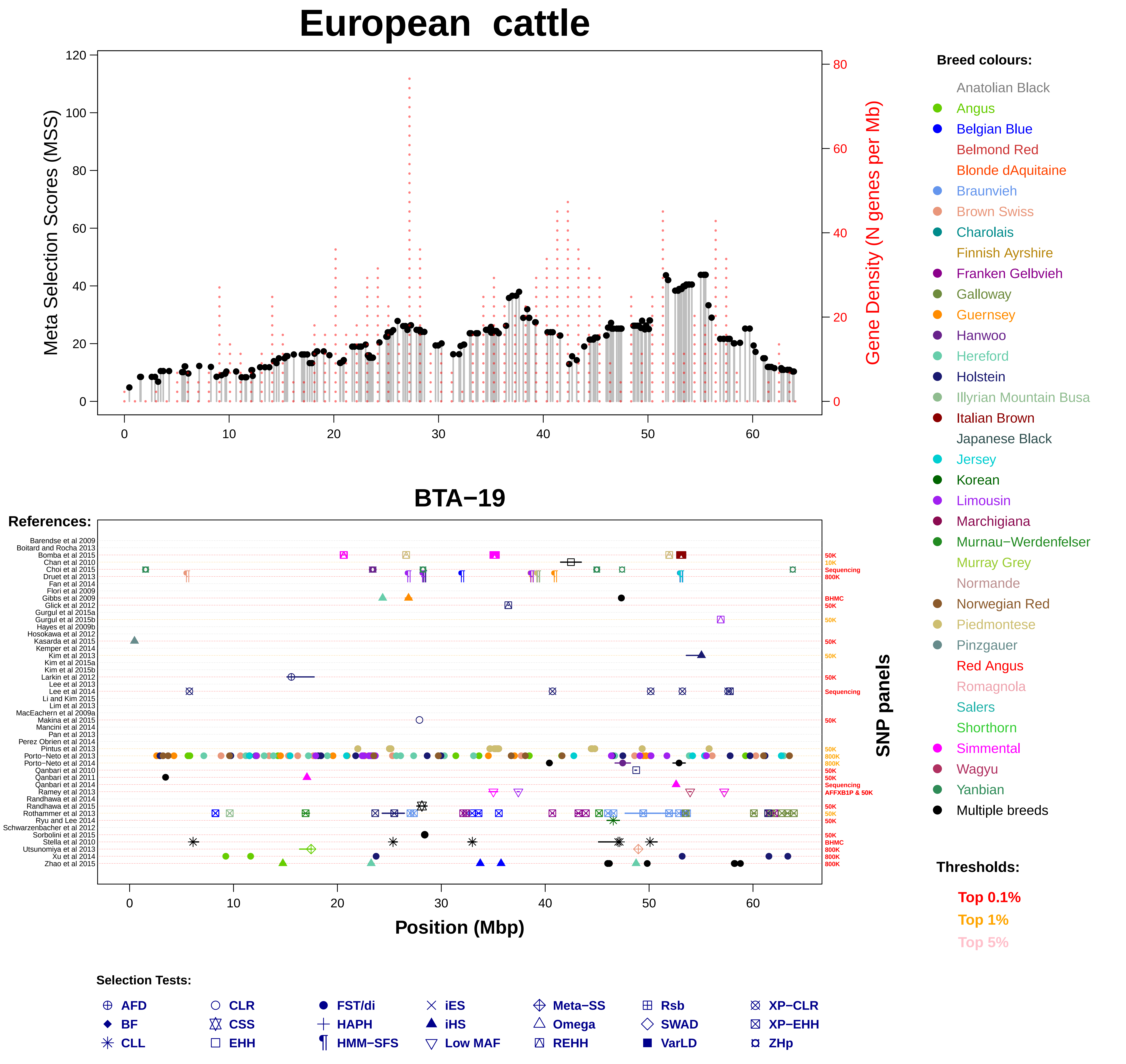

European cattle
Meta Selection Scores (MSS)
Gene Density (N genes per Mb)
BTA−19
References:
Barendse et al 2009
Boitard and Rocha 2013
Bomba et al 2015
Chan et al 2010
Choi et al 2015
Druet et al 2013
Fan et al 2014
Flori et al 2009
Gibbs et al 2009
Glick et al 2012
Gurgul et al 2015a
Gurgul et al 2015b
Hayes et al 2009b
Hosokawa et al 2012
Kasarda et al 2015
Kemper et al 2014
Kim et al 2013
Kim et al 2015a
Kim et al 2015b
Larkin et al 2012
Lee et al 2013
Lee et al 2014
Li and Kim 2015
Lim et al 2013
MacEachern et al 2009a
Makina et al 2015
Mancini et al 2014
Pan et al 2013
Perez Obrien et al 2014
Pintus et al 2013
Porto−Neto et al 2013
Porto−Neto et al 2014
Qanbari et al 2010
Qanbari et al 2011
Qanbari et al 2014
Ramey et al 2013
Randhawa et al 2014
Randhawa et al 2015
Rothammer et al 2013
Ryu and Lee 2014
Schwarzenbacher et al 2012
Sorbolini et al 2015
Stella et al 2010
Utsunomiya et al 2013
Xu et al 2014
Zhao et al 2015
SNP panels
50K
10K
Sequencing
800K
BHMC
AFFXB1P & 50K
Position (Mbp)
Breed colours:
Anatolian Black
Angus
Belgian Blue
Belmond Red
Blonde dAquitaine
Braunvieh
Brown Swiss
Charolais
Finnish Ayrshire
Franken Gelbvieh
Galloway
Guernsey
Hanwoo
Hereford
Holstein
Illyrian Mountain Busa
Italian Brown
Japanese Black
Jersey
Korean
Limousin
Marchigiana
Murnau−Werdenfelser
Murray Grey
Normande
Norwegian Red
Piedmontese
Pinzgauer
Red Angus
Romagnola
Salers
Shorthorn
Simmental
Wagyu
Yanbian
Multiple breeds
Thresholds:
Top 0.1%
Top 1%
Top 5%
Selection Tests:
AFD
BF
CLL
CLR
CSS
EHH
FST/di
HAPH
HMM−SFS
iES
iHS
Low MAF
Meta−SS
Omega
REHH
Rsb
SWAD
VarLD
XP−CLR
XP−EHH
ZHp

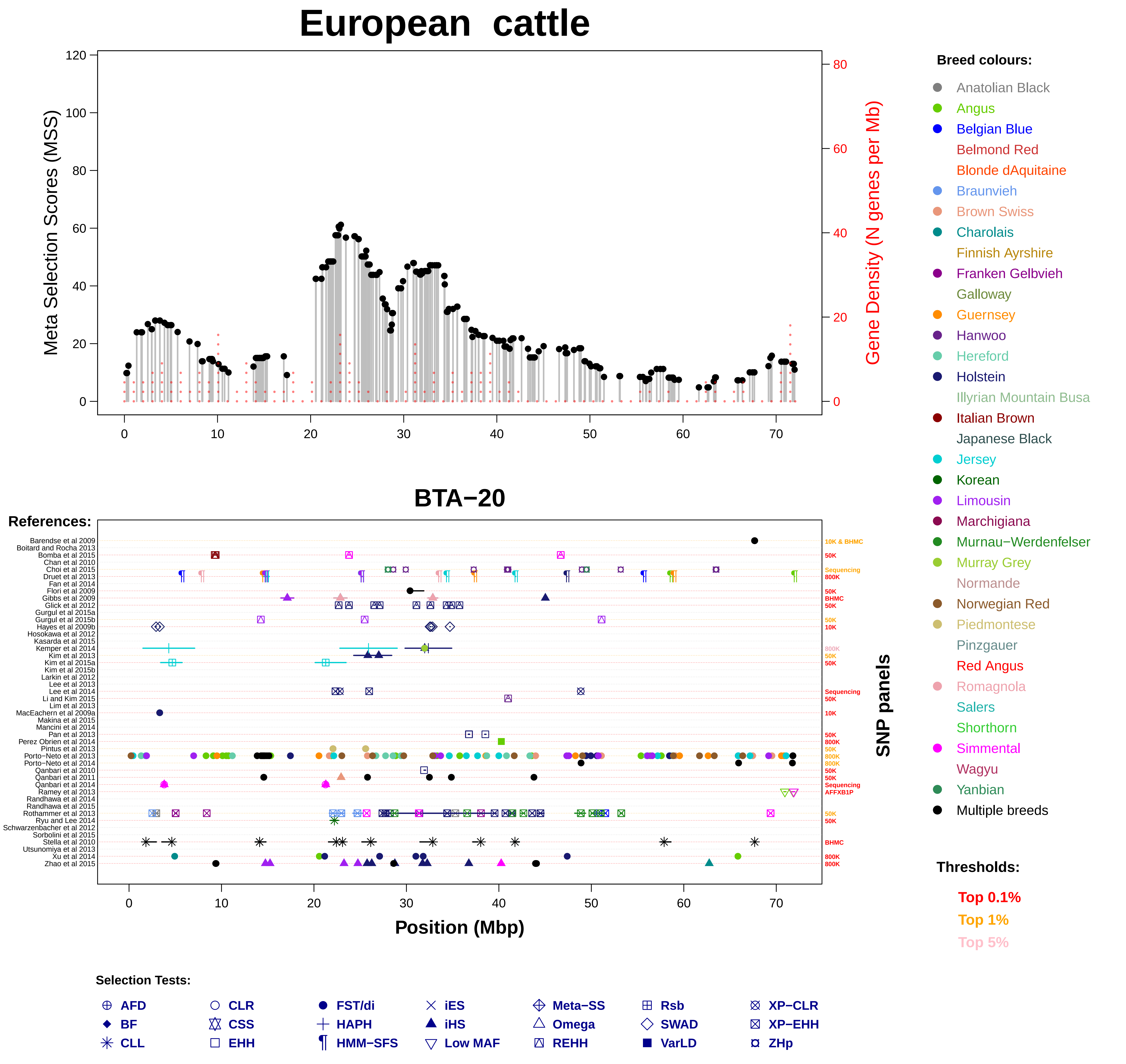
European cattle
Meta Selection Scores (MSS)
Gene Density (N genes per Mb)
BTA−20
References:
SNP panels
Position (Mbp)
Breed colours:
Anatolian Black
Angus
Belgian Blue
Belmond Red
Blonde dAquitaine
Braunvieh
Brown Swiss
Charolais
Finnish Ayrshire
Franken Gelbvieh
Galloway
Guernsey
Hanwoo
Hereford
Holstein
Illyrian Mountain Busa
Italian Brown
Japanese Black
Jersey
Korean
Limousin
Marchigiana
Murnau−Werdenfelser
Murray Grey
Normande
Norwegian Red
Piedmontese
Pinzgauer
Red Angus
Romagnola
Salers
Shorthorn
Simmental
Wagyu
Yanbian
Multiple breeds
Thresholds:
Top 0.1%
Top 1%
Top 5%
Selection Tests:
AFD
BF
CLL
CLR
CSS
EHH
FST/di
HAPH
HMM−SFS
iES
iHS
Low MAF
Meta−SS
Omega
REHH
Rsb
SWAD
VarLD
XP−CLR
XP−EHH
ZHp
Barendse et al 2009
Boitard and Rocha 2013
Bomba et al 2015
Chan et al 2010
Choi et al 2015
Druet et al 2013
Fan et al 2014
Flori et al 2009
Gibbs et al 2009
Glick et al 2012
Gurgul et al 2015a
Gurgul et al 2015b
Hayes et al 2009b
Hosokawa et al 2012
Kasarda et al 2015
Kemper et al 2014
Kim et al 2013
Kim et al 2015a
Kim et al 2015b
Larkin et al 2012
Lee et al 2013
Lee et al 2014
Li and Kim 2015
Lim et al 2013
MacEachern et al 2009a
Makina et al 2015
Mancini et al 2014
Pan et al 2013
Perez Obrien et al 2014
Pintus et al 2013
Porto−Neto et al 2013
Porto−Neto et al 2014
Qanbari et al 2010
Qanbari et al 2011
Qanbari et al 2014
Ramey et al 2013
Randhawa et al 2014
Randhawa et al 2015
Rothammer et al 2013
Ryu and Lee 2014
Schwarzenbacher et al 2012
Sorbolini et al 2015
Stella et al 2010
Utsunomiya et al 2013
Xu et al 2014
Zhao et al 2015
10K & BHMC
50K
Sequencing
800K
50K
BHMC
50K
50K
10K
800K
50K
50K
Sequencing
50K
10K
50K
800K
50K
800K
800K
50K
50K
Sequencing
AFFXB1P
50K
50K
BHMC
800K
800K

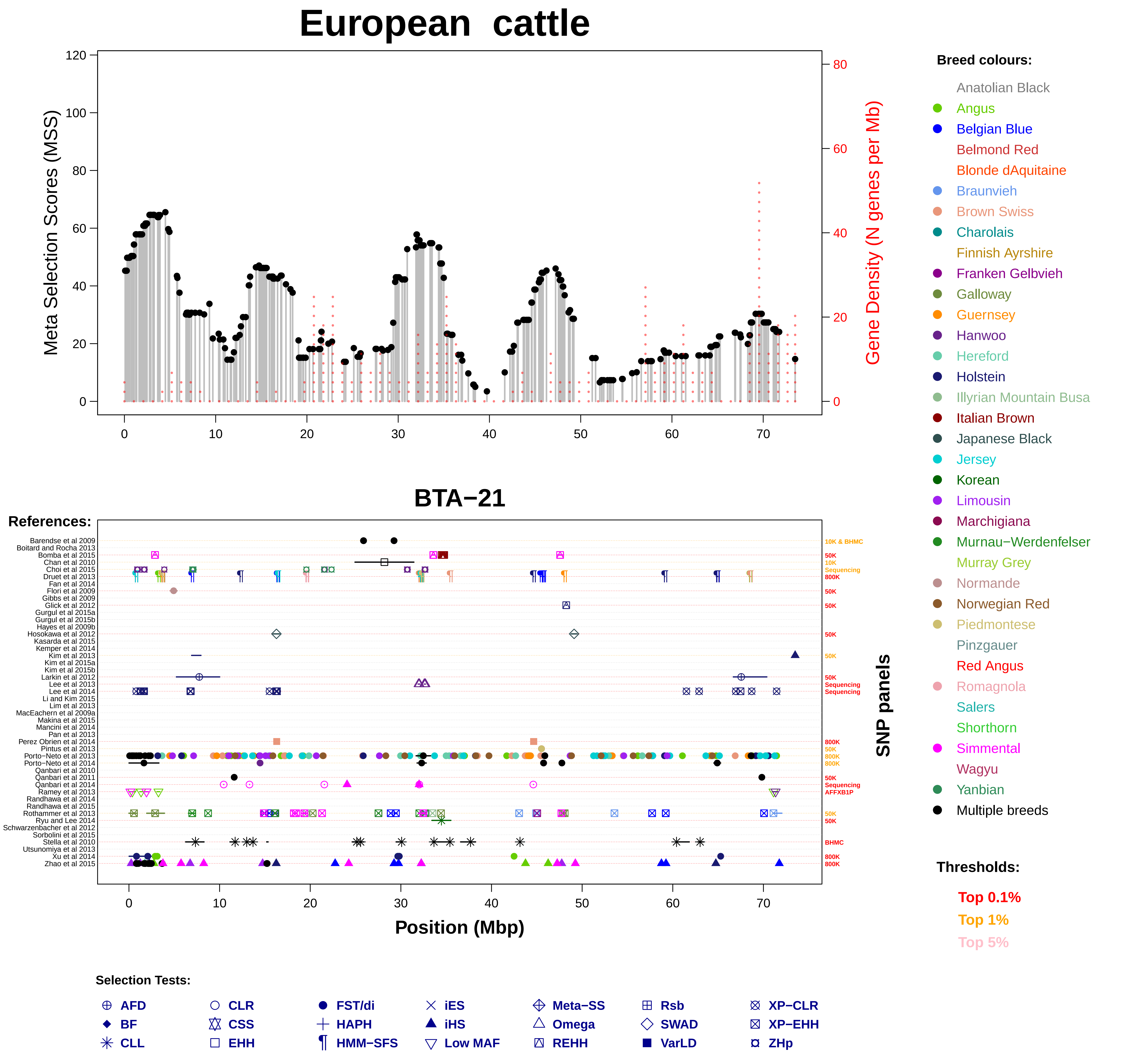
European cattle
BTA−21
Meta Selection Scores (MSS)
Gene Density (N genes per Mb)
Position (Mbp)
References:
SNP panels
Breed colours:
Anatolian Black
Angus
Belgian Blue
Belmond Red
Blonde dAquitaine
Braunvieh
Brown Swiss
Charolais
Finnish Ayrshire
Franken Gelbvieh
Galloway
Guernsey
Hanwoo
Hereford
Holstein
Illyrian Mountain Busa
Italian Brown
Japanese Black
Jersey
Korean
Limousin
Marchigiana
Murnau−Werdenfelser
Murray Grey
Normande
Norwegian Red
Piedmontese
Pinzgauer
Red Angus
Romagnola
Salers
Shorthorn
Simmental
Wagyu
Yanbian
Multiple breeds
Thresholds:
Top 0.1%
Top 1%
Top 5%
Barendse et al 2009
Boitard and Rocha 2013
Bomba et al 2015
Chan et al 2010
Choi et al 2015
Druet et al 2013
Fan et al 2014
Flori et al 2009
Gibbs et al 2009
Glick et al 2012
Gurgul et al 2015a
Gurgul et al 2015b
Hayes et al 2009b
Hosokawa et al 2012
Kasarda et al 2015
Kemper et al 2014
Kim et al 2013
Kim et al 2015a
Kim et al 2015b
Larkin et al 2012
Lee et al 2013
Lee et al 2014
Li and Kim 2015
Lim et al 2013
MacEachern et al 2009a
Makina et al 2015
Mancini et al 2014
Pan et al 2013
Perez Obrien et al 2014
Pintus et al 2013
Porto−Neto et al 2013
Porto−Neto et al 2014
Qanbari et al 2010
Qanbari et al 2011
Qanbari et al 2014
Ramey et al 2013
Randhawa et al 2014
Randhawa et al 2015
Rothammer et al 2013
Ryu and Lee 2014
Schwarzenbacher et al 2012
Sorbolini et al 2015
Stella et al 2010
Utsunomiya et al 2013
Xu et al 2014
Zhao et al 2015
10K & BHMC
50K
10K
Sequencing
800K
50K
50K
50K
50K
50K
Sequencing
Sequencing
800K
50K
800K
800K
50K
Sequencing
AFFXB1P
50K
50K
BHMC
800K
800K
Selection Tests:
AFD
BF
CLL
CLR
CSS
EHH
FST/di
HAPH
HMM−SFS
iES
iHS
Low MAF
Meta−SS
Omega
REHH
Rsb
SWAD
VarLD
XP−CLR
XP−EHH
ZHp

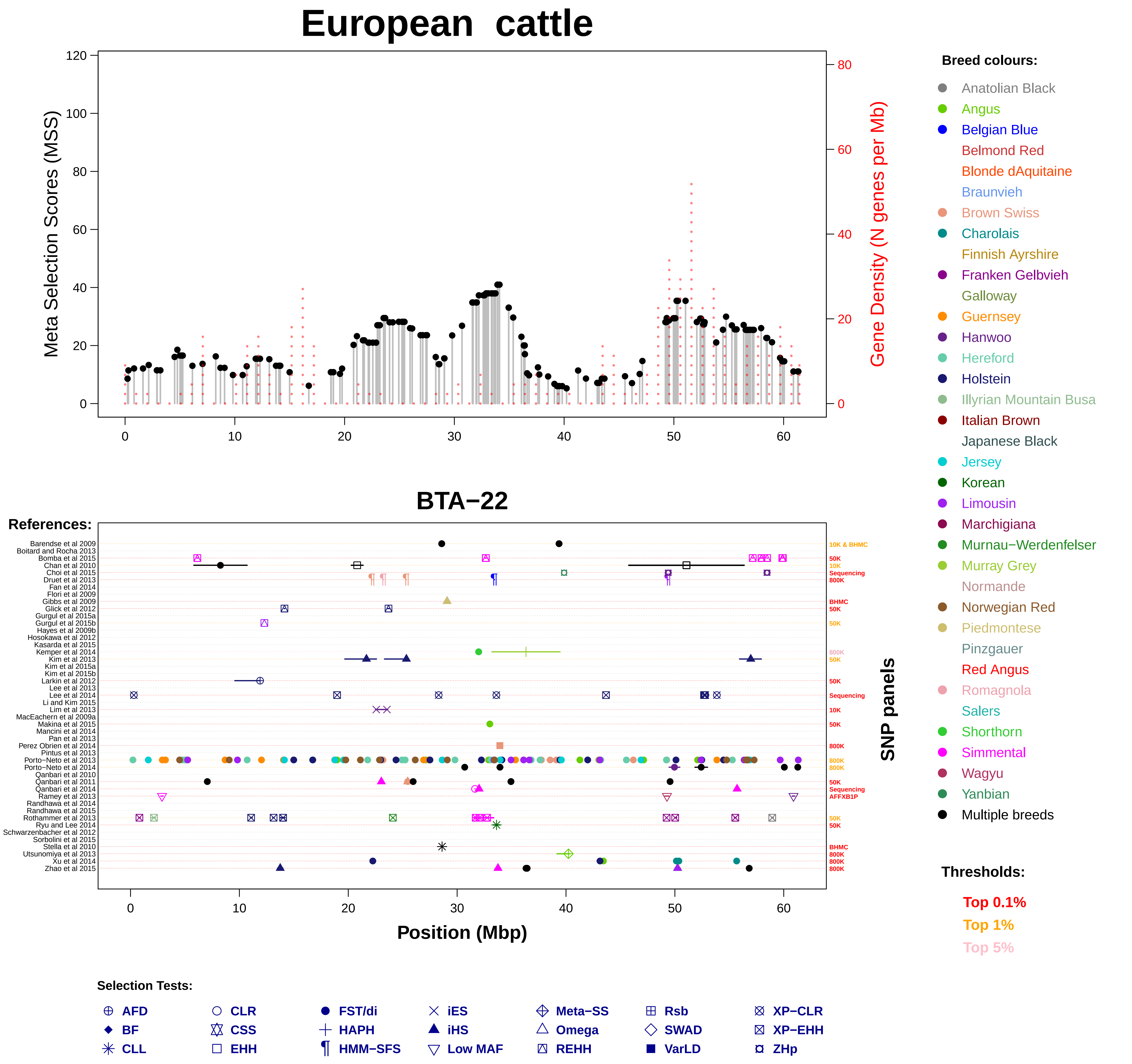
European cattle
Meta Selection Scores (MSS)
Gene Density (N genes per Mb)
BTA−22
References:
Barendse et al 2009
Boitard and Rocha 2013
Bomba et al 2015
Chan et al 2010
Choi et al 2015
Druet et al 2013
Fan et al 2014
Flori et al 2009
Gibbs et al 2009
Glick et al 2012
Gurgul et al 2015a
Gurgul et al 2015b
Hayes et al 2009b
Hosokawa et al 2012
Kasarda et al 2015
Kemper et al 2014
Kim et al 2013
Kim et al 2015a
Kim et al 2015b
Larkin et al 2012
Lee et al 2013
Lee et al 2014
Li and Kim 2015
Lim et al 2013
MacEachern et al 2009a
Makina et al 2015
Mancini et al 2014
Pan et al 2013
Perez Obrien et al 2014
Pintus et al 2013
Porto−Neto et al 2013
Porto−Neto et al 2014
Qanbari et al 2010
Qanbari et al 2011
Qanbari et al 2014
Ramey et al 2013
Randhawa et al 2014
Randhawa et al 2015
Rothammer et al 2013
Ryu and Lee 2014
Schwarzenbacher et al 2012
Sorbolini et al 2015
Stella et al 2010
Utsunomiya et al 2013
Xu et al 2014
Zhao et al 2015
SNP panels
10K & BHMC
50K
10K
Sequencing
800K
BHMC
50K
50K
800K
50K
50K
Sequencing
10K
50K
800K
800K
800K
50K
Sequencing
AFFXB1P
50K
50K
BHMC
800K
800K
800K
Position (Mbp)
Breed colours:
Anatolian Black
Angus
Belgian Blue
Belmond Red
Blonde dAquitaine
Braunvieh
Brown Swiss
Charolais
Finnish Ayrshire
Franken Gelbvieh
Galloway
Guernsey
Hanwoo
Hereford
Holstein
Illyrian Mountain Busa
Italian Brown
Japanese Black
Jersey
Korean
Limousin
Marchigiana
Murnau−Werdenfelser
Murray Grey
Normande
Norwegian Red
Piedmontese
Pinzgauer
Red Angus
Romagnola
Salers
Shorthorn
Simmental
Wagyu
Yanbian
Multiple breeds
Thresholds:
Top 0.1%
Top 1%
Top 5%
Selection Tests:
AFD
BF
CLL
CLR
CSS
EHH
FST/di
HAPH
HMM−SFS
iES
iHS
Low MAF
Meta−SS
Omega
REHH
Rsb
SWAD
VarLD
XP−CLR
XP−EHH
ZHp

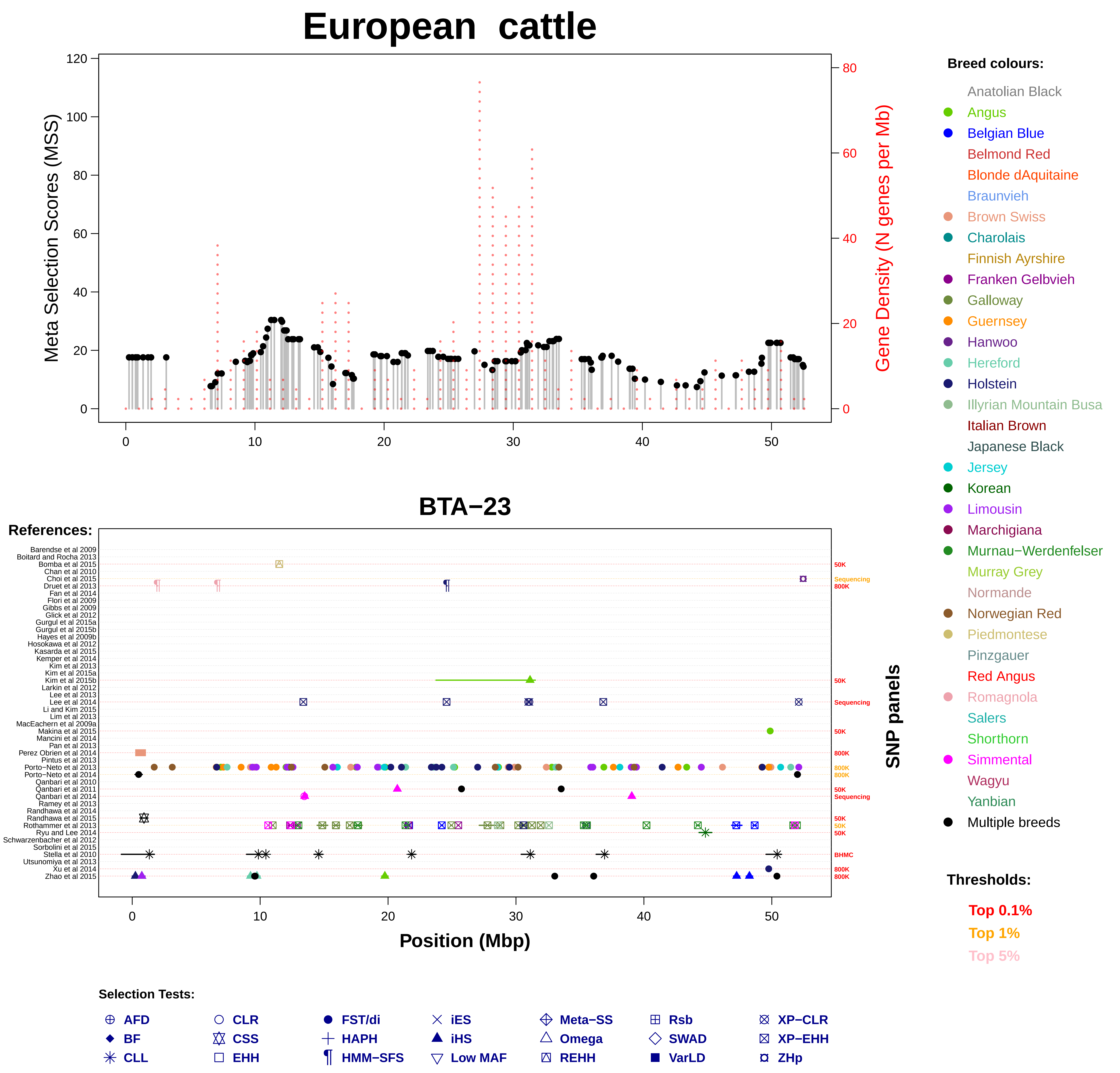
European cattle
Meta Selection Scores (MSS)
Gene Density (N genes per Mb)
BTA−23
References:
Barendse et al 2009
Boitard and Rocha 2013
Bomba et al 2015
Chan et al 2010
Choi et al 2015
Druet et al 2013
Fan et al 2014
Flori et al 2009
Gibbs et al 2009
Glick et al 2012
Gurgul et al 2015a
Gurgul et al 2015b
Hayes et al 2009b
Hosokawa et al 2012
Kasarda et al 2015
Kemper et al 2014
Kim et al 2013
Kim et al 2015a
Kim et al 2015b
Larkin et al 2012
Lee et al 2013
Lee et al 2014
Li and Kim 2015
Lim et al 2013
MacEachern et al 2009a
Makina et al 2015
Mancini et al 2014
Pan et al 2013
Perez Obrien et al 2014
Pintus et al 2013
Porto−Neto et al 2013
Porto−Neto et al 2014
Qanbari et al 2010
Qanbari et al 2011
Qanbari et al 2014
Ramey et al 2013
Randhawa et al 2014
Randhawa et al 2015
Rothammer et al 2013
Ryu and Lee 2014
Schwarzenbacher et al 2012
Sorbolini et al 2015
Stella et al 2010
Utsunomiya et al 2013
Xu et al 2014
Zhao et al 2015
SNP panels
Position (Mbp)
Breed colours:
Anatolian Black
Angus
Belgian Blue
Belmond Red
Blonde dAquitaine
Braunvieh
Brown Swiss
Charolais
Finnish Ayrshire
Franken Gelbvieh
Galloway
Guernsey
Hanwoo
Hereford
Holstein
Illyrian Mountain Busa
Italian Brown
Japanese Black
Jersey
Korean
Limousin
Marchigiana
Murnau−Werdenfelser
Murray Grey
Normande
Norwegian Red
Piedmontese
Pinzgauer
Red Angus
Romagnola
Salers
Shorthorn
Simmental
Wagyu
Yanbian
Multiple breeds
Thresholds:
Top 0.1%
Top 1%
Top 5%
Selection Tests:
AFD
BF
CLL
CLR
CSS
EHH
FST/di
HAPH
HMM−SFS
iES
iHS
Low MAF
Meta−SS
Omega
REHH
Rsb
SWAD
VarLD
XP−CLR
XP−EHH
ZHp

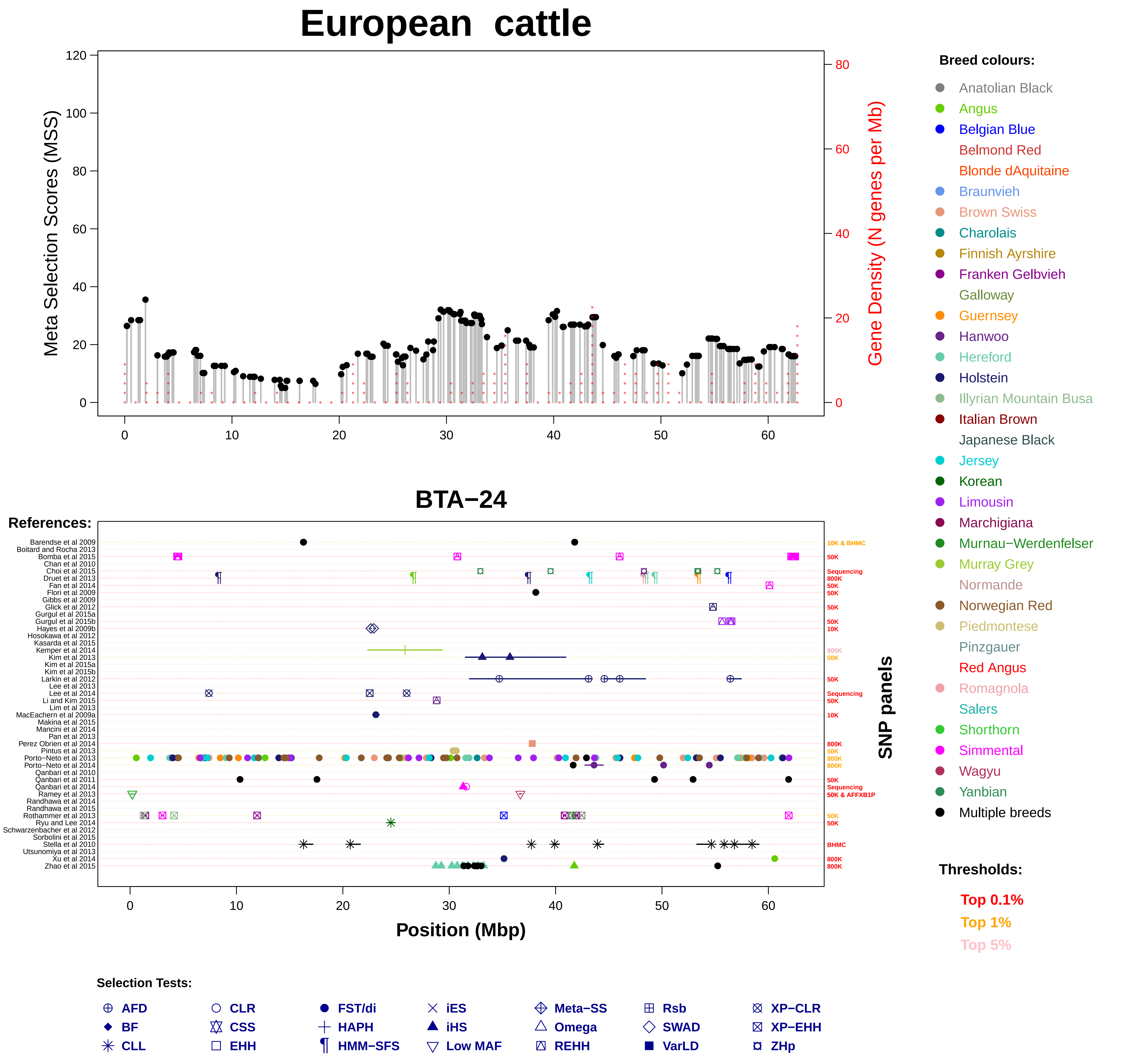
European cattle
Meta Selection Scores (MSS)
Gene Density (N genes per Mb)
BTA−24
References:
Barendse et al 2009
Boitard and Rocha 2013
Bomba et al 2015
Chan et al 2010
Choi et al 2015
Druet et al 2013
Fan et al 2014
Flori et al 2009
Gibbs et al 2009
Glick et al 2012
Gurgul et al 2015a
Gurgul et al 2015b
Hayes et al 2009b
Hosokawa et al 2012
Kasarda et al 2015
Kemper et al 2014
Kim et al 2013
Kim et al 2015a
Kim et al 2015b
Larkin et al 2012
Lee et al 2013
Lee et al 2014
Li and Kim 2015
Lim et al 2013
MacEachern et al 2009a
Makina et al 2015
Mancini et al 2014
Pan et al 2013
Perez Obrien et al 2014
Pintus et al 2013
Porto−Neto et al 2013
Porto−Neto et al 2014
Qanbari et al 2010
Qanbari et al 2011
Qanbari et al 2014
Ramey et al 2013
Randhawa et al 2014
Randhawa et al 2015
Rothammer et al 2013
Ryu and Lee 2014
Schwarzenbacher et al 2012
Sorbolini et al 2015
Stella et al 2010
Utsunomiya et al 2013
Xu et al 2014
Zhao et al 2015
10K & BHMC
50K
Sequencing
800K
10K
BHMC
50K & AFFXB1P
SNP panels
Position (Mbp)
Breed colours:
Anatolian Black
Angus
Belgian Blue
Belmond Red
Blonde dAquitaine
Braunvieh
Brown Swiss
Charolais
Finnish Ayrshire
Franken Gelbvieh
Galloway
Guernsey
Hanwoo
Hereford
Holstein
Illyrian Mountain Busa
Italian Brown
Japanese Black
Jersey
Korean
Limousin
Marchigiana
Murnau−Werdenfelser
Murray Grey
Normande
Norwegian Red
Piedmontese
Pinzgauer
Red Angus
Romagnola
Salers
Shorthorn
Simmental
Wagyu
Yanbian
Multiple breeds
Thresholds:
Top 0.1%
Top 1%
Top 5%
Selection Tests:
AFD
BF
CLL
CLR
CSS
EHH
FST/di
HAPH
HMM−SFS
iES
iHS
Low MAF
Meta−SS
Omega
REHH
Rsb
SWAD
VarLD
XP−CLR
XP−EHH
ZHp

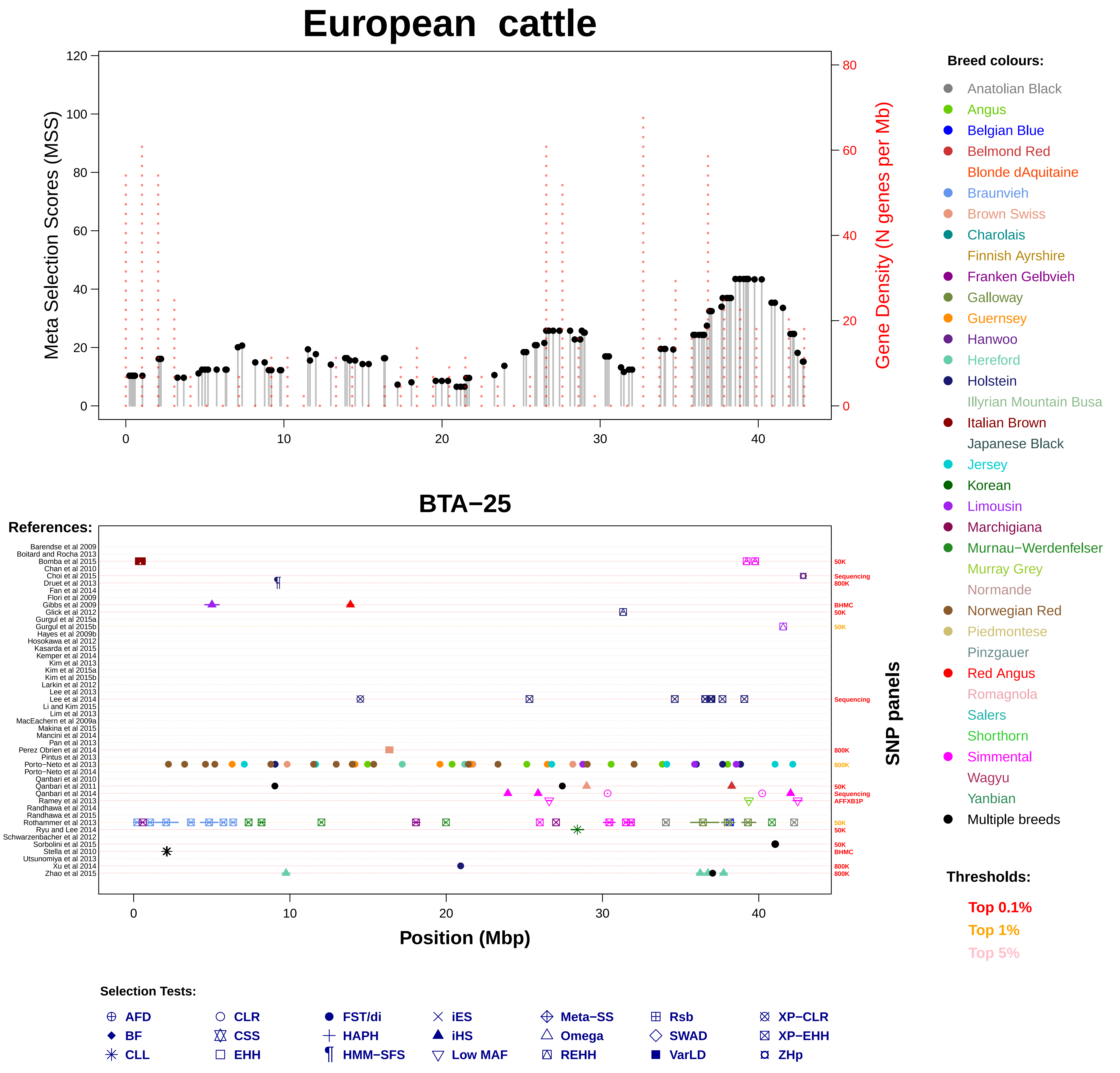
European cattle
Meta Selection Scores (MSS)
Gene Density (N genes per Mb)
0
20
40
60
80
100
120
0
20
40
60
80
0
10
20
30
40
BTA−25
References:
Barendse et al 2009
Boitard and Rocha 2013
Bomba et al 2015
Chan et al 2010
Choi et al 2015
Druet et al 2013
Fan et al 2014
Flori et al 2009
Gibbs et al 2009
Glick et al 2012
Gurgul et al 2015a
Gurgul et al 2015b
Hayes et al 2009b
Hosokawa et al 2012
Kasarda et al 2015
Kemper et al 2014
Kim et al 2013
Kim et al 2015a
Kim et al 2015b
Larkin et al 2012
Lee et al 2013
Lee et al 2014
Li and Kim 2015
Lim et al 2013
MacEachern et al 2009a
Makina et al 2015
Mancini et al 2014
Pan et al 2013
Perez Obrien et al 2014
Pintus et al 2013
Porto−Neto et al 2013
Porto−Neto et al 2014
Qanbari et al 2010
Qanbari et al 2011
Qanbari et al 2014
Ramey et al 2013
Randhawa et al 2014
Randhawa et al 2015
Rothammer et al 2013
Ryu and Lee 2014
Schwarzenbacher et al 2012
Sorbolini et al 2015
Stella et al 2010
Utsunomiya et al 2013
Xu et al 2014
Zhao et al 2015
50K
Sequencing
800K
BHMC
50K
50K
Sequencing
800K
800K
50K
Sequencing
AFFXB1P
50K
50K
50K
BHMC
800K
800K
SNP panels
Position (Mbp)
Breed colours:
Anatolian Black
Angus
Belgian Blue
Belmond Red
Blonde dAquitaine
Braunvieh
Brown Swiss
Charolais
Finnish Ayrshire
Franken Gelbvieh
Galloway
Guernsey
Hanwoo
Hereford
Holstein
Illyrian Mountain Busa
Italian Brown
Japanese Black
Jersey
Korean
Limousin
Marchigiana
Murnau−Werdenfelser
Murray Grey
Normande
Norwegian Red
Piedmontese
Pinzgauer
Red Angus
Romagnola
Salers
Shorthorn
Simmental
Wagyu
Yanbian
Multiple breeds
Thresholds:
Top 0.1%
Top 1%
Top 5%
Selection Tests:
AFD
BF
CLL
CLR
CSS
EHH
FST/di
HAPH
HMM−SFS
iES
iHS
Low MAF
Meta−SS
Omega
REHH
Rsb
SWAD
VarLD
XP−CLR
XP−EHH
ZHp

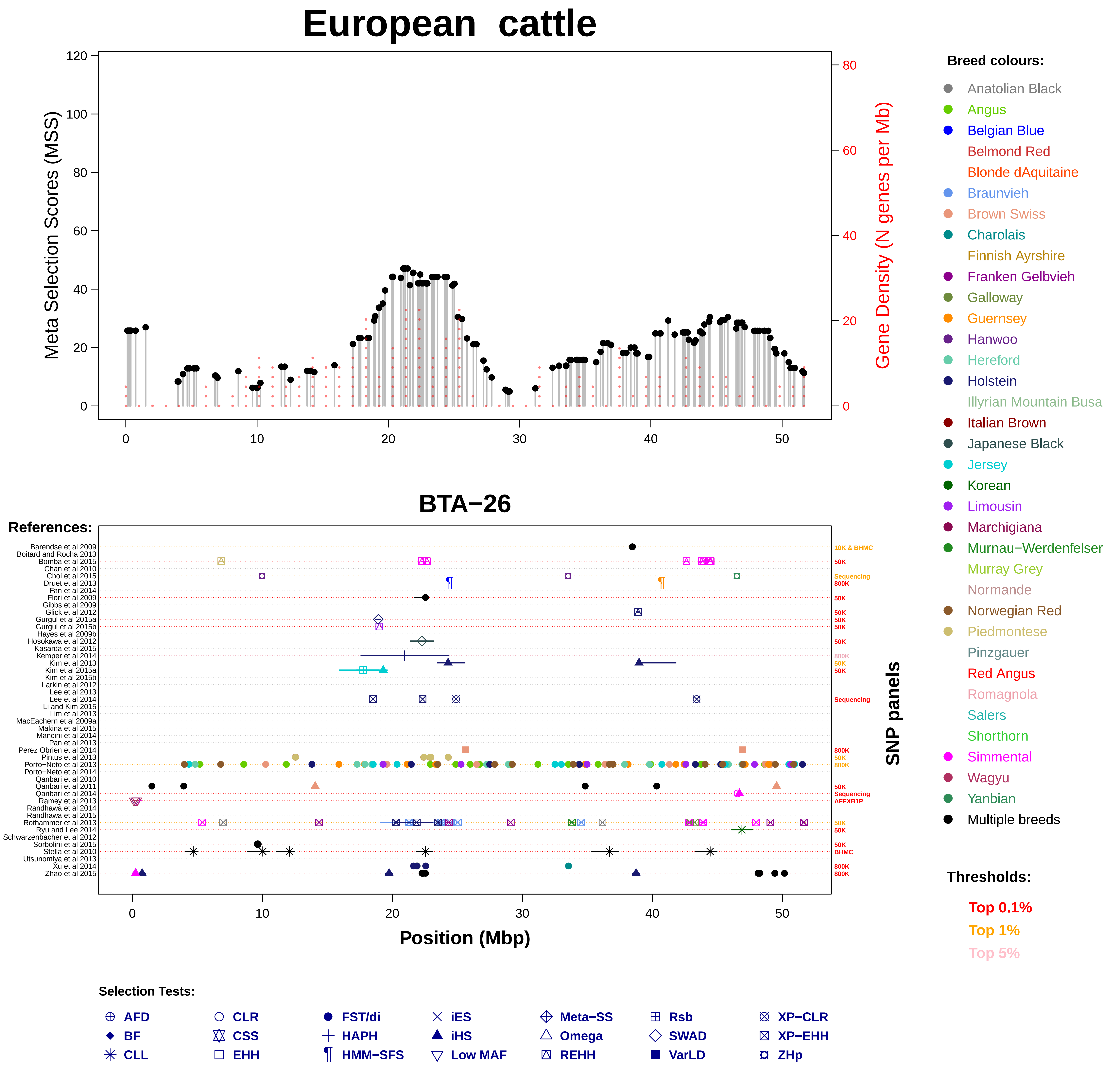
European cattle
Meta Selection Scores (MSS)
Gene Density (N genes per Mb)
BTA−26
References:
Barendse et al 2009
Boitard and Rocha 2013
Bomba et al 2015
Chan et al 2010
Choi et al 2015
Druet et al 2013
Fan et al 2014
Flori et al 2009
Gibbs et al 2009
Glick et al 2012
Gurgul et al 2015a
Gurgul et al 2015b
Hayes et al 2009b
Hosokawa et al 2012
Kasarda et al 2015
Kemper et al 2014
Kim et al 2013
Kim et al 2015a
Kim et al 2015b
Larkin et al 2012
Lee et al 2013
Lee et al 2014
Li and Kim 2015
Lim et al 2013
MacEachern et al 2009a
Makina et al 2015
Mancini et al 2014
Pan et al 2013
Perez Obrien et al 2014
Pintus et al 2013
Porto−Neto et al 2013
Porto−Neto et al 2014
Qanbari et al 2010
Qanbari et al 2011
Qanbari et al 2014
Ramey et al 2013
Randhawa et al 2014
Randhawa et al 2015
Rothammer et al 2013
Ryu and Lee 2014
Schwarzenbacher et al 2012
Sorbolini et al 2015
Stella et al 2010
Utsunomiya et al 2013
Xu et al 2014
Zhao et al 2015
10K & BHMC
50K
Sequencing
800K
AFFXB1P
BHMC
SNP panels
Position (Mbp)
Breed colours:
Anatolian Black
Angus
Belgian Blue
Belmond Red
Blonde dAquitaine
Braunvieh
Brown Swiss
Charolais
Finnish Ayrshire
Franken Gelbvieh
Galloway
Guernsey
Hanwoo
Hereford
Holstein
Illyrian Mountain Busa
Italian Brown
Japanese Black
Jersey
Korean
Limousin
Marchigiana
Murnau−Werdenfelser
Murray Grey
Normande
Norwegian Red
Piedmontese
Pinzgauer
Red Angus
Romagnola
Salers
Shorthorn
Simmental
Wagyu
Yanbian
Multiple breeds
Thresholds:
Top 0.1%
Top 1%
Top 5%
Selection Tests:
AFD
BF
CLL
CLR
CSS
EHH
FST/di
HAPH
HMM−SFS
iES
iHS
Low MAF
Meta−SS
Omega
REHH
Rsb
SWAD
VarLD
XP−CLR
XP−EHH
ZHp

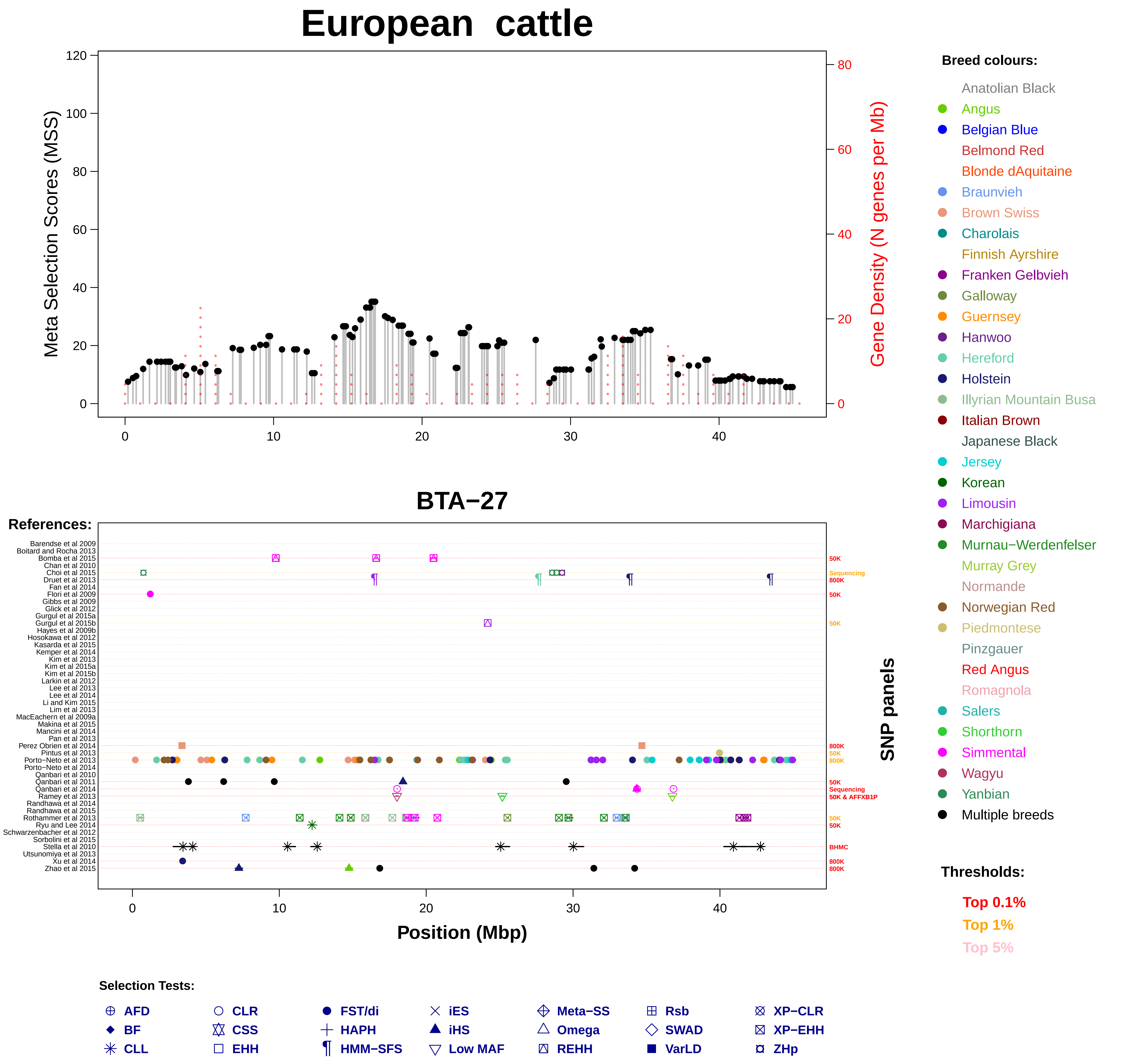

European cattle
Meta Selection Scores (MSS)
Gene Density (N genes per Mb)
BTA−27
References:
Barendse et al 2009
Boitard and Rocha 2013
Bomba et al 2015
Chan et al 2010
Choi et al 2015
Druet et al 2013
Fan et al 2014
Flori et al 2009
Gibbs et al 2009
Glick et al 2012
Gurgul et al 2015a
Gurgul et al 2015b
Hayes et al 2009b
Hosokawa et al 2012
Kasarda et al 2015
Kemper et al 2014
Kim et al 2013
Kim et al 2015a
Kim et al 2015b
Larkin et al 2012
Lee et al 2013
Lee et al 2014
Li and Kim 2015
Lim et al 2013
MacEachern et al 2009a
Makina et al 2015
Mancini et al 2014
Pan et al 2013
Perez Obrien et al 2014
Pintus et al 2013
Porto−Neto et al 2013
Porto−Neto et al 2014
Qanbari et al 2010
Qanbari et al 2011
Qanbari et al 2014
Ramey et al 2013
Randhawa et al 2014
Randhawa et al 2015
Rothammer et al 2013
Ryu and Lee 2014
Schwarzenbacher et al 2012
Sorbolini et al 2015
Stella et al 2010
Utsunomiya et al 2013
Xu et al 2014
Zhao et al 2015
50K
Sequencing
800K
50K
50K
800K
50K
800K
50K
Sequencing
50K & AFFXB1P
50K
50K
BHMC
800K
800K
SNP panels
Position (Mbp)
Breed colours:
Anatolian Black
Angus
Belgian Blue
Belmond Red
Blonde dAquitaine
Braunvieh
Brown Swiss
Charolais
Finnish Ayrshire
Franken Gelbvieh
Galloway
Guernsey
Hanwoo
Hereford
Holstein
Illyrian Mountain Busa
Italian Brown
Japanese Black
Jersey
Korean
Limousin
Marchigiana
Murnau−Werdenfelser
Murray Grey
Normande
Norwegian Red
Piedmontese
Pinzgauer
Red Angus
Romagnola
Salers
Shorthorn
Simmental
Wagyu
Yanbian
Multiple breeds
Thresholds:
Top 0.1%
Top 1%
Top 5%
Selection Tests:
AFD
BF
CLL
CLR
CSS
EHH
FST/di
HAPH
HMM−SFS
iES
iHS
Low MAF
Meta−SS
Omega
REHH
Rsb
SWAD
VarLD
XP−CLR
XP−EHH
ZHp

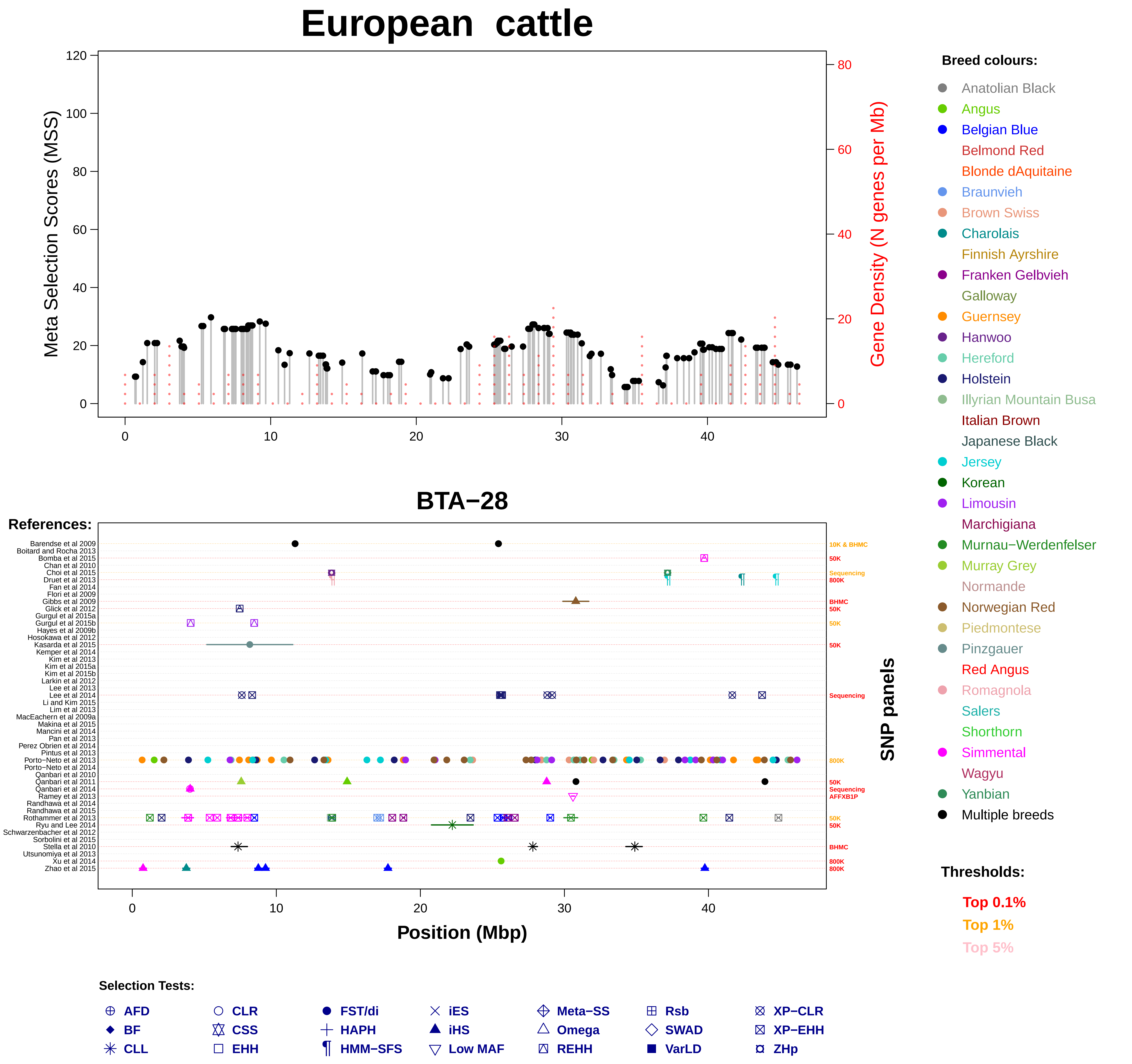

European cattle
Meta Selection Scores (MSS)
Gene Density (N genes per Mb)
BTA−28
References:
Barendse et al 2009
Boitard and Rocha 2013
Bomba et al 2015
Chan et al 2010
Choi et al 2015
Druet et al 2013
Fan et al 2014
Flori et al 2009
Gibbs et al 2009
Glick et al 2012
Gurgul et al 2015a
Gurgul et al 2015b
Hayes et al 2009b
Hosokawa et al 2012
Kasarda et al 2015
Kemper et al 2014
Kim et al 2013
Kim et al 2015a
Kim et al 2015b
Larkin et al 2012
Lee et al 2013
Lee et al 2014
Li and Kim 2015
Lim et al 2013
MacEachern et al 2009a
Makina et al 2015
Mancini et al 2014
Pan et al 2013
Perez Obrien et al 2014
Pintus et al 2013
Porto−Neto et al 2013
Porto−Neto et al 2014
Qanbari et al 2010
Qanbari et al 2011
Qanbari et al 2014
Ramey et al 2013
Randhawa et al 2014
Randhawa et al 2015
Rothammer et al 2013
Ryu and Lee 2014
Schwarzenbacher et al 2012
Sorbolini et al 2015
Stella et al 2010
Utsunomiya et al 2013
Xu et al 2014
Zhao et al 2015
10K & BHMC
50K
Sequencing
800K
BHMC
50K
50K
50K
Sequencing
800K
50K
Sequencing
AFFXB1P
50K
50K
BHMC
800K
800K
SNP panels
Position (Mbp)
Breed colours:
Anatolian Black
Angus
Belgian Blue
Belmond Red
Blonde dAquitaine
Braunvieh
Brown Swiss
Charolais
Finnish Ayrshire
Franken Gelbvieh
Galloway
Guernsey
Hanwoo
Hereford
Holstein
Illyrian Mountain Busa
Italian Brown
Japanese Black
Jersey
Korean
Limousin
Marchigiana
Murnau−Werdenfelser
Murray Grey
Normande
Norwegian Red
Piedmontese
Pinzgauer
Red Angus
Romagnola
Salers
Shorthorn
Simmental
Wagyu
Yanbian
Multiple breeds
Thresholds:
Top 0.1%
Top 1%
Top 5%
Selection Tests:
AFD
BF
CLL
CLR
CSS
EHH
FST/di
HAPH
HMM−SFS
iES
iHS
Low MAF
Meta−SS
Omega
REHH
Rsb
SWAD
VarLD
XP−CLR
XP−EHH
ZHp

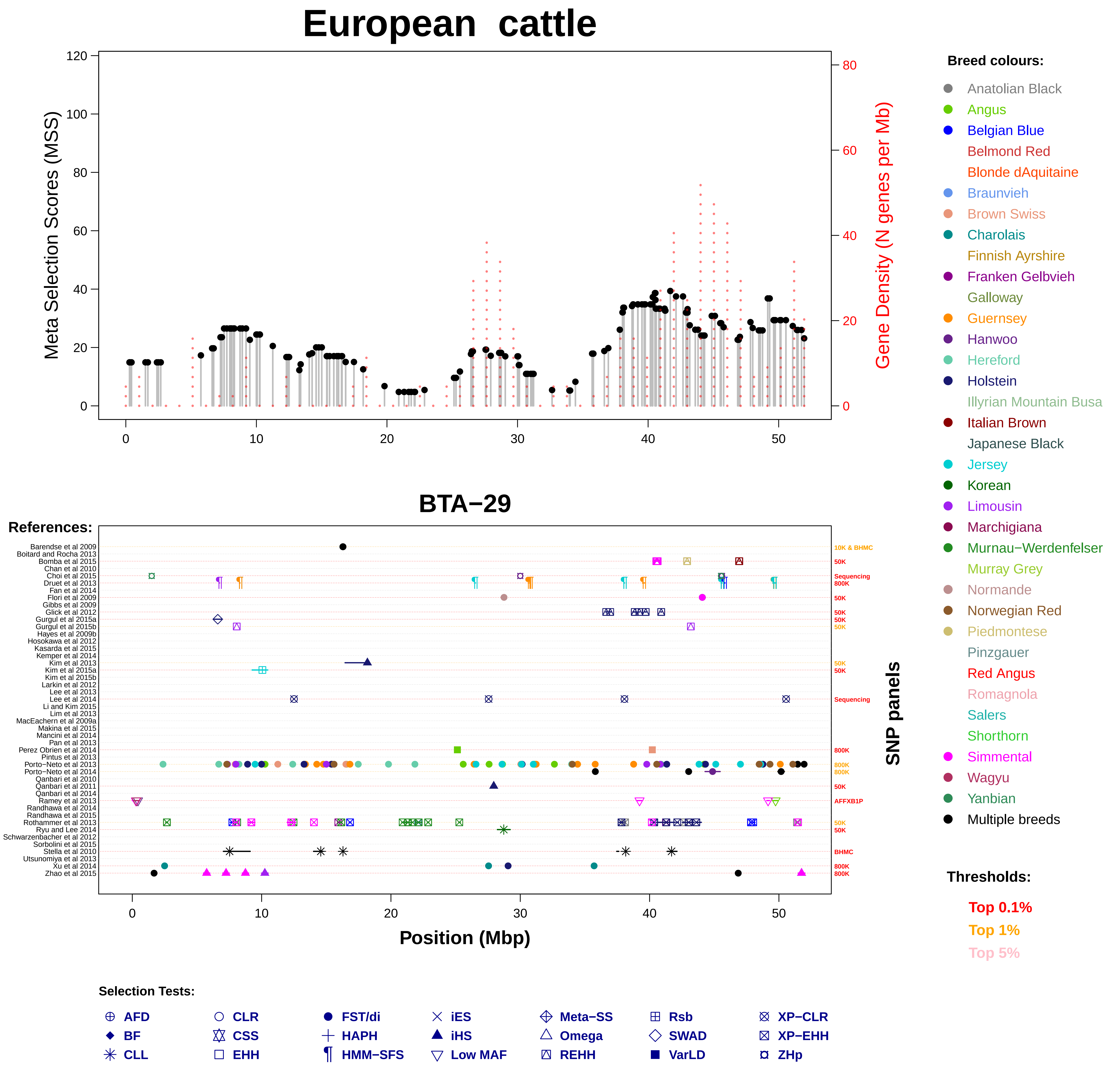
European cattle
Meta Selection Scores (MSS)
Gene Density (N genes per Mb)
BTA−29
References:
Barendse et al 2009
Boitard and Rocha 2013
Bomba et al 2015
Chan et al 2010
Choi et al 2015
Druet et al 2013
Fan et al 2014
Flori et al 2009
Gibbs et al 2009
Glick et al 2012
Gurgul et al 2015a
Gurgul et al 2015b
Hayes et al 2009b
Hosokawa et al 2012
Kasarda et al 2015
Kemper et al 2014
Kim et al 2013
Kim et al 2015a
Kim et al 2015b
Larkin et al 2012
Lee et al 2013
Lee et al 2014
Li and Kim 2015
Lim et al 2013
MacEachern et al 2009a
Makina et al 2015
Mancini et al 2014
Pan et al 2013
Perez Obrien et al 2014
Pintus et al 2013
Porto−Neto et al 2013
Porto−Neto et al 2014
Qanbari et al 2010
Qanbari et al 2011
Qanbari et al 2014
Ramey et al 2013
Randhawa et al 2014
Randhawa et al 2015
Rothammer et al 2013
Ryu and Lee 2014
Schwarzenbacher et al 2012
Sorbolini et al 2015
Stella et al 2010
Utsunomiya et al 2013
Xu et al 2014
Zhao et al 2015
10K & BHMC
50K
Sequencing
800K
AFFXB1P
BHMC
SNP panels
Position (Mbp)
Breed colours:
Anatolian Black
Angus
Belgian Blue
Belmond Red
Blonde dAquitaine
Braunvieh
Brown Swiss
Charolais
Finnish Ayrshire
Franken Gelbvieh
Galloway
Guernsey
Hanwoo
Hereford
Holstein
Illyrian Mountain Busa
Italian Brown
Japanese Black
Jersey
Korean
Limousin
Marchigiana
Murnau−Werdenfelser
Murray Grey
Normande
Norwegian Red
Piedmontese
Pinzgauer
Red Angus
Romagnola
Salers
Shorthorn
Simmental
Wagyu
Yanbian
Multiple breeds
Thresholds:
Top 0.1%
Top 1%
Top 5%
Selection Tests:
AFD
BF
CLL
CLR
CSS
EHH
FST/di
HAPH
HMM−SFS
iES
iHS
Low MAF
Meta−SS
Omega
REHH
Rsb
SWAD
VarLD
XP−CLR
XP−EHH
ZHp
